# Synthesis of Nitrogen‐Containing Heterocyclic Scaffolds and Applications in Scaffold Hopping: Development of an Aripiprazole Analog

**DOI:** 10.1002/cmdc.70505

**Published:** 2026-10-25

**Authors:** Temitayo Omowumi Alegbejo Price, Pieterjan Winant, Paulo Eliandro da Silva Júnior, Shaiani Maria Gil de Melo, Pedro Henrique Gobira, Julia Possamai Gimenes, Pedro Tenório Teixeira Fernandes Leite, Vinícius Gonçalves Maltarollo, Gustavo Henrique Goulart Trossini, A. Ganesan, Sâmia Regiane Lourenço Joca, Wim Dehaen, Flavio da Silva Emery

**Affiliations:** ^1^ Faculty of Pharmaceutical Sciences of Ribeirão Preto Department of Pharmaceutical Sciences University of Sao Paulo Ribeirão Preto Brazil; ^2^ Department of Chemistry KU Leuven Leuven Belgium; ^3^ School of Pharmacy University of East Anglia Norwich Norfolk; ^4^ Translational Neuropsychiatry Unit Department of Clinical Medicine Aarhus University Aarhus Denmark; ^5^ Departamento de Produtos Farmacêuticos Faculdade de Farmácia Universidade Federal de Minas Gerais Belo Horizonte Brazil; ^6^ Faculty of Pharmaceutical Sciences University of Sao Paulo São Paulo Brazil; ^7^ Institute of Advanced Studies of the University of São Paulo University of São Paulo São Paulo SP Brazil; ^8^ Department of Biomedicine Aarhus University Aarhus Denmark

**Keywords:** aripiprazole, fragments, fused heterocycles, nitrogen, scaffold hopping

## Abstract

Heteroaromatic ring systems play a crucial role in drug development because they modulate physicochemical properties and expand the range of molecular interactions compared to non‐heterocyclic aromatic rings. They are also widely represented among approved drugs. Among heteroatoms, nitrogen is the most prevalent in the diverse scaffold space of heterocyclic drugs, including those generated by fragment‐based drug discovery approach. In this work, we show the synthesis of a set of novel or underexplored nitrogen heterocyclic fragments, obtained in three to five steps in moderate yields. In total, 12 fused [6,6] saturated/unsaturated bicycles were synthesized, which fulfill the general rule of fragment‐like compounds. These heterocycles increased the fragment space of the CRAFT library and showed potential application in scaffold‐hopping strategies for drug design. As a proof of concept, 2,6‐naphthyridin‐3‐ol **2** was used to replace the 7‐hydroxy‐3,4‐dihydroquinolin‐2(1*H*)‐one core of aripiprazole **41**, generating 3‐(4‐(4‐(2,3‐dichlorophenyl)piperazin‐1‐yl)butoxy)‐2,6‐naphthyridine **40,** that effectively blocked cocaine‐induced hyperactivity in rodents at 20 mg/kg without inducing catalepsy, displaying a pharmacological profile similar to aripiprazole and demonstrating promising antipsychotic potential.

## Graphical abstract

A series of 12 novel fused [6,6] saturated and unsaturated nitrogen bicycles is synthesized in three to five steps and expands the CRAFT library's fragment space. A 2,6‐naphthyridin‐3‐ol core replaces the dihydroquinolinone scaffold of aripiprazole, a scaffold‐hopping strategy. The resulting analog blocks cocaine‐induced hyperactivity in rodents at 20 mg/kg without catalepsy, a catalepsy‐free antipsychotic profile.

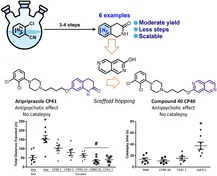

## 1 Introduction

A large number of drugs available in the market contain heterocycles, with nitrogen being the predominant heteroatom. An analysis of the US FDA database for small‐molecule drugs revealed that over 80% of approved drugs in the past decade contain nitrogen heterocyclic scaffolds [1]. Prior to 2013, this proportion was approximately 60% [2], representing a striking relative increase of about 36% and further underscoring the importance of such scaffolds in drug discovery. Nitrogen‐containing heterocycles exhibit remarkable structural diversity, making this chemical space a valuable source not only for medicinal chemistry but also for a wide range of scientific fields [1, 3]. However, medicinal chemists still rely on the most frequent heterocycles (pyridine, piperidine, pyrrolidine, piperazine, and pyrimidine) [1] in drug discovery campaigns, mainly due to their synthetic accessibility and sociability for vectorial functionalization. As a result, the number of new heterocyclic ring systems per year in drugs is relatively low. However, the search for new scaffolds has intensified in recent years. A 2022 publication reported 378 ring systems used in approved drugs and 450 unique ring systems in clinical trials, of which 280 (62%) had not previously been used in any marketed drug [4]. Altogether, this corresponds to about 658 distinct ring systems across drugs and clinical candidates. When comparing the number of new ring systems present in drugs approved up to 2014 with those approved in 2022, approximately 27 additional ring systems were introduced, an average of around 3 to 4 new scaffolds per year [4, 5].

Building on this, in 2009, Pitt and colleagues identified a set of 22 synthetically tractable scaffolds that warrant further exploration by medicinal chemists [6]. To date, nine of these scaffolds have been successfully investigated. Compounds **2** and **3** are representative examples from this set. Silva Júnior and co‐workers developed a novel synthetic route to the 1,4‐dihydro‐2,6‐naphthyridin‐3(2*H*)‐one **1** (Scheme 1) [7], which is synthesized in 5 steps, including a late‐stage oxidation to a pseudoaromatic tautomer 2,6‐naphthyridin‐3‐ol **2** to the fully aromatic ring system. Interestingly, **1** represents a particularly attractive heterocycle for medicinal chemistry owing to its favorable molecular properties, including logP, logD, and its ability to “escape from flatland” [8], in comparison to **2** (Figure 1).

**FIGURE 1:**
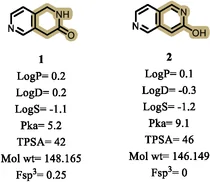
The structure of compounds **1** and **2** and some of their physicochemical properties.

In fact, **2** and **3** in Figure 2 represent the pseudoaromatic tautomers with amide functionality and ortho‐quinoidal fusion [9] (Figure 2). These compounds and related analogs (Figure 2) remain underexplored in the literature, with several of the derivatives described herein representing previously unreported fragments. In this work, we report the synthesis of a series of partially saturated heterocycles, along with a new fully aromatic ring system. The utility of these scaffolds is further demonstrated through a scaffold‐hopping strategy, enabling the development of an antipsychotic compound.

**FIGURE 2:**
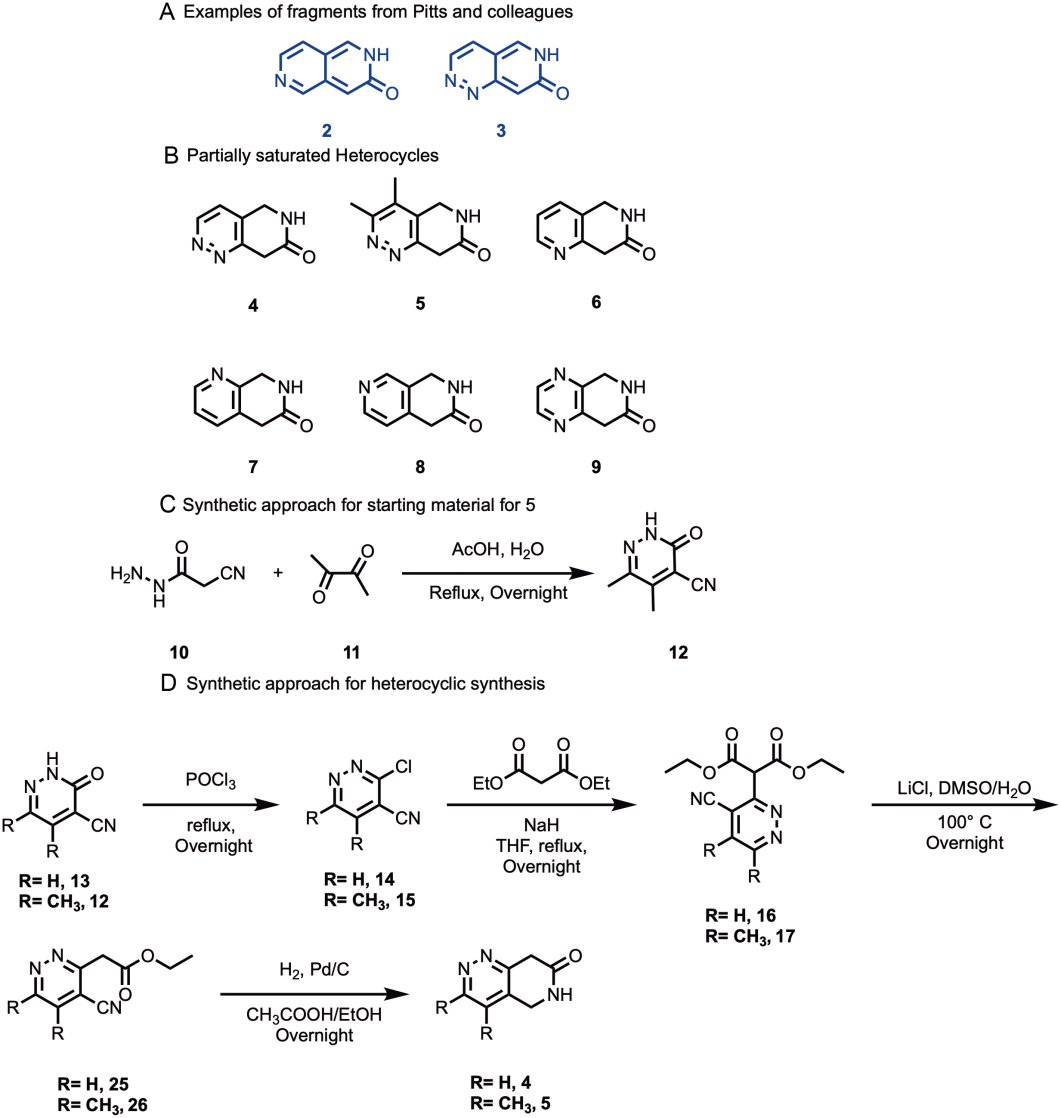
The proposed compounds and the general scheme for their synthesis.

## 2 Results and Discussion

### 2.1 Synthesis and Molecular Properties

Using the 5,8‐dihydropyrido[4,3‐*c*]pyridazin‐7(6*H*)‐one **4** as a model, we planned the synthetic approach based on previously published results, which involved the reductive heterocyclization of ester‐substituted cyano‐azines. In order to obtain **4**, we started chlorinating the commercially available 3‐oxo‐2,3‐dihydropyridazine‐4‐carbonitrile **13** to obtain the chlorinated intermediate **14** by using POCl_3_ in good yield (50%) (Figure 2) [10].

The chlorinated intermediate was then submitted to a nucleophilic substitution with diethylmalonate, which yielded the dicarboxylated heterocycle **16** (Figure 3), which was decarboxylated via Krapcho’s method [11] using LiCl to afford **25** (Figure 4), which was followed by reductive heterocyclization to the 5,8‐dihydropyrido[4,3‐*c*]pyridazin‐7(6*H*)‐one **3** (55%) (Figure 1). The dimethylated analog was obtained by using 5,6‐dimethyl‐3‐oxo‐2,3‐dihydropyridazine‐4‐carbonitrile **12** as starting material, which was synthesized by the reaction of the correspondent hydrazide **10** with dimethyl glyoxal **11** in water using catalytic acetic acid to obtain the desired heterocycle in moderate yield (58%) (Figure 2) [12]. Using the same process described earlier for **3**, it was possible to obtain **4** in 62% yield (Figure 2).

**FIGURE 3:**
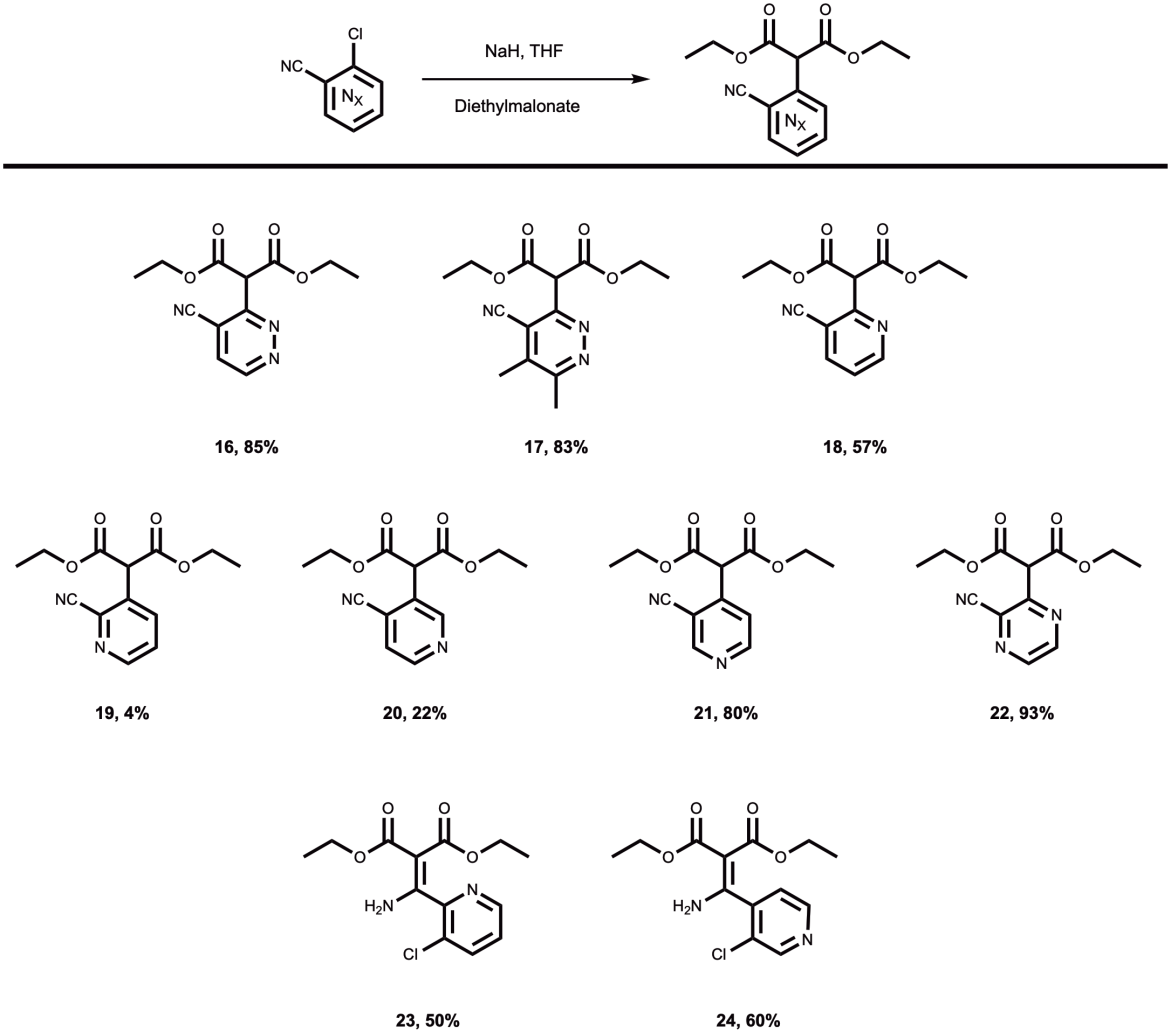
Alkylated pyridines and a pyrazine obtained after nucleophilic substitution and side products **23** and **24**.

**FIGURE 4:**
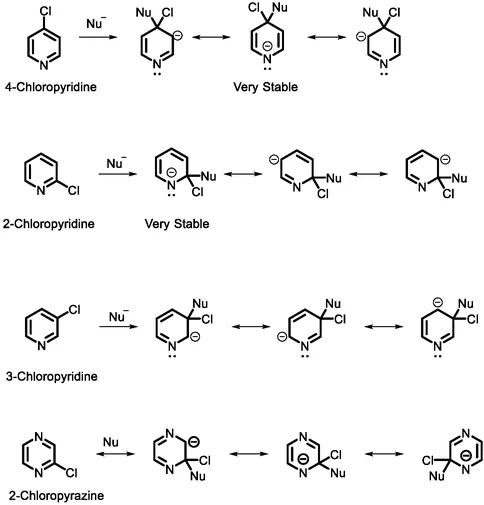
Relative stability of the intermediate carbanion’s of chloropyridines and chloropyrazine.

We expanded the synthesis to other azines, including diverse dihydronapthyridinones and a dihydropyrazinone, by using the same synthetic approaches described above. However, unlike the pyridazines, alkylation of the chloro‐cyanoazines required heating and long time to consume the starting material [13]. Interestingly, the reaction gave good yields when the cyanoazines have chlorine substitution at the more electro‐deficient position C‐4 or C‐2 to produce compounds **18** and **21** in very good yields of 57% and 80%, respectively. The chloro‐cyanopyrazine gave the best yield of 93% to afford **22** (Figure 3).

However, when chlorine is located at C‐3, as in 2‐ and 4‐cyanoazines, the desired alkylated products **19** and **20** are formed only in low yields. Under these conditions, the reaction preferentially proceeds via nucleophilic attack at the nitrile group, leading predominantly to enamine derivatives **23** and **24** (Figure 3), which are obtained in moderate yields (50%–60%). The difference in reactivity of the studied azines is driven by both inductive and mesomeric effects, which makes 3‐chloropyridine less reactive than 2‐ or 4‐chloropyridine and also increase reactivity of the pyrazine comparing to the other starting material (Figure 4).

The chlorinated intermediates were then submitted to a nucleophilic substitution with diethylmalonate, which yielded the dicarboxylated heterocycle **18–22,** that was used in two different ways (Figure 5). In the first (Pathway A), it was decarboxylated via Krapcho’s method [11] using LiCl to afford **27–30**, which was followed by reductive heterocyclization to **1**, **6, 8,** and **9** (Figure 2). Pathway B followed the reductive heterocyclization to obtain the 5‐ethyl carboxylate derivative **31–34** (Figure 5). Interestingly, in both cases, the reduction of the nitrile, under pressure, required 2 equivalents of Pd/C instead of the usual catalytic amount, which suggests potential strong coordination of the heterocycle with palladium, poisoning the catalyst. The method for obtaining the fused heterocycles was successfully reproduced on multigram scale with similar efficiency.

**FIGURE 5:**
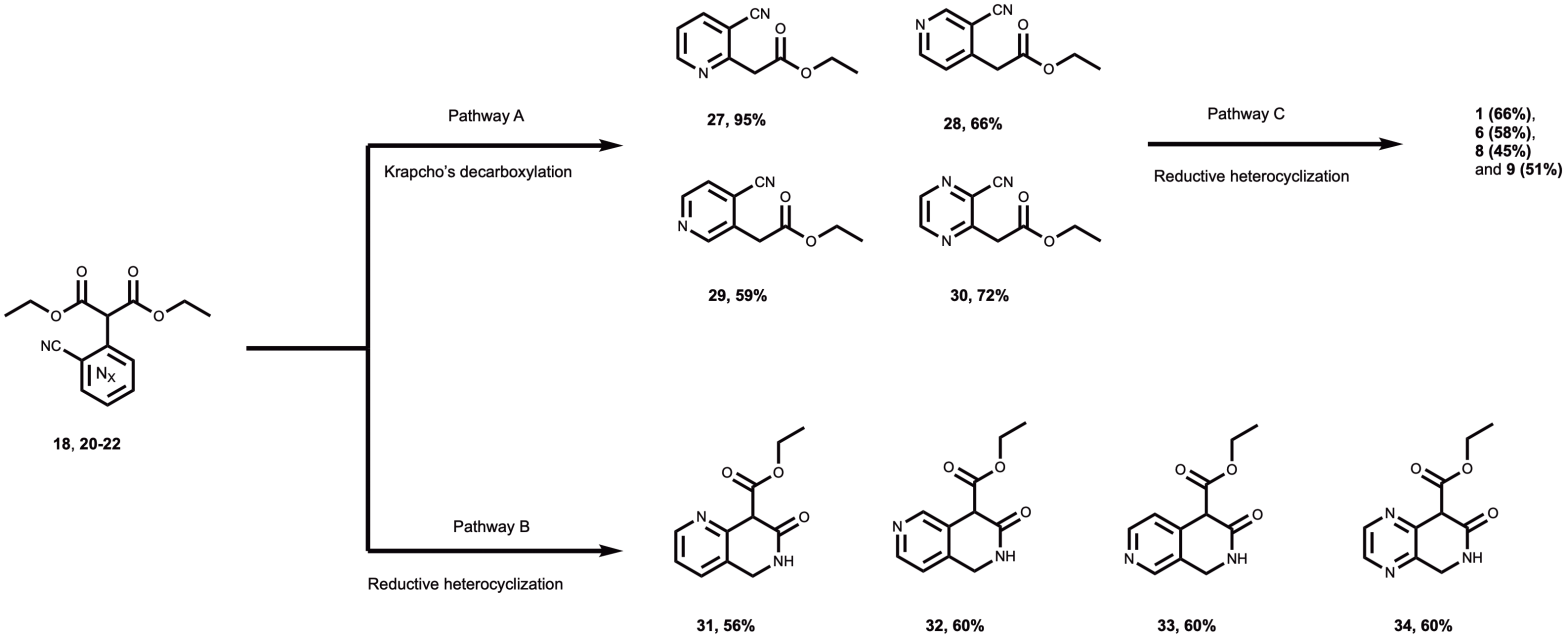
Divergent synthesis of parent lactams **1**, **6**, **8**, and **9** (Pathways A and C) and ethoxycarbonyl lactams **31** **–34** (Pathway B) from dicarboxylated adducts **18** and **20–** **22**.

The proposed mechanism for the Pd/C‐catalyzed reductive cyclization proceeds through a sequential reduction and ring‐closing cascade. The reaction initiates with the heterogeneous hydrogenation of the nitrile group on the palladium surface, passing through a transient imine intermediate to generate the requisite primary amine in situ. The primary amine immediately undergoes intramolecular nucleophilic attack at the adjacent ester carbonyl, releasing ethanol and driving the thermodynamically favorable closure of the cyclic lactam core.

After successfully diversifying the heterocyclic library, we explored the dehydrogenation of **6** to obtain the fully aromatic naphthyridine **35**. This transformation was carried out following the method reported by Silva Junior et al [7]. Using activated charcoal in xylene at 120 °C under oxygen. The target product **35** was obtained in 65% yield on a small scale (Scheme 1). Similarly, fully oxidized product **2** from **1** was obtained, demonstrating the versatility of the developed synthetic route. Attempts to modify the reaction conditions were unsuccessful. Replacing pure oxygen with atmospheric air did not yield the desired product. Likewise, substituting xylene with toluene as the reaction solvent failed to produce the target compound.

**SCHEME 1:**
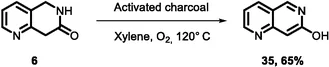
Synthetic route for compound **35** and **1**

To evaluate the potential utility of these scaffolds, we predicted their physicochemical properties and performed a comparative analysis across the series. This assessment included key descriptors relevant to drug‐likeness, such as molecular weight (MW), lipophilicity (ClogP), hydrogen bond donors (HBD), hydrogen bond acceptors (HBA), polar surface area (TPSA), and other parameters associated with solubility and permeability.

Across the series, the values for MW, ClogP, and HBD fell within the “rule of three” (RO3) criteria for fragments (MW ≤ 300, ClogP ≤ 3, HBD ≤ 3). Scaffolds **1**, **2**, **6**, **7**, **8**, and **35** fully satisfied RO3, with TPSA ≤ 60 and HBA ≤ 3, making them particularly attractive starting points for drug discovery programs (Table 1). All scaffolds, except **35** and **2**, displayed Fsp^3^ ≥ 0.25, suggesting three‐dimensionality and saturation associated with greater molecular flexibility and potentially improved solubility compared to flat aromatic systems. The heavy atom count (HAC) for all scaffolds was below 17, which supports their suitability as fragment‐like structures (Table 1). Compounds **4**, **5,** and **9** met all RO3 criteria except for HBA as each scaffold contains an additional nitrogen atom (Table 1). Scaffolds **31–34** showed higher HBA values due to the presence of ethyl ester substituents, which also contributed to increased TPSA (Table 1). These findings highlight the diversity of the synthesized cores and the presence of several scaffolds with favorable profiles for fragment‐based or lead optimization strategies.

**TABLE 1:** Predicted physicochemical properties for the synthesized compounds.

Property	1	2	4	5	6	7	8	9	31	32	33	34	35
**MW**	148	146	149	177	148	148	148	149	220	220	220	221	146
**log P**	0,6	−0,1	−0,5	−0,1	0,4	0,4	0,6	−0,5	0	−1,2	0	−0,9	0,4
**HBD**	1	1	1	1	1	1	1	1	1	1	1	1	1
**HBA**	3	3	4	4	3	3	3	4	5	5	5	6	3
**log D**	0,6	−1,3	−0,5	−0,1	0,4	0,4	0,6	−0,5	0	−1,2	0	−0,9	−1,3
**log S**	−1,4	−1	−0,6	−1,1	−1,2	−1,3	−1,4	−0,6	−1,4	−0,5	−1,3	−0,7	−1,4
**pKa**	5,2	9,1	11,2	4,5	5,2	5,8	5,2	10,5	3,9	3,8	4,6	2,7	9
**TPSA**	42	46	54,9	54,9	42	42	42	54,9	68,3	68,3	68,3	81,2	46
**Fsp3**	0,25	0	0,29	0,44	0,25	0,25	0,25	0,29	0,36	0,36	0,36	0,4	0
**HAC**	11	11	11	13	11	11	11	11	16	16	16	16	11

*Note:* log P, logarithm of partition‐coefficient; log S, logarithm of solubility‐coefficient; log P, calculated lipophilicity; log D, logarithm of distribution‐coefficient (pH 5.5–7.4).

Abbreviations: CDD, collaborative Drug Discovery Vault; HAC, heavy atoms count; HBA, hydrogen bond acceptor; HBD, hydrogen bond donor; MW, molecular weight; TPSA, topological polar surface area.

In drug discovery, scaffold hopping is widely employed as a strategy to generate new drug candidates by modifying the core structure of known bioactive molecules while preserving their key pharmacophoric elements [14]. This approach has enabled the development of several clinically successful drugs [15]. For example, Bosutinib (Wyeth, 2012) emerged from Gefitinib (AstraZeneca, 2003) through the replacement of an isoquinoline ring with a quinazoline core [15]. Similarly, Sorafenib (Bayer, 2007) was developed from Lenvatinib (Eisai, 2005) by substituting the isoquinoline scaffold with a pyridine ring [15]. Such structural transformations not only enhance biological activity or selectivity but are also frequently used to navigate around existing intellectual property by generating novel, synthetically accessible chemical entities that retain the desired therapeutic profile [14].

Scaffold hopping involves computer‐aided searches for active compounds containing alternative core structures [14, 15]. More recently, scaffold hopping has become a valuable tool in small‐molecule drug design, applied to lead compounds or approved drugs as a targeted approach to improve pharmacological properties [14, 15]. Since scaffold **2** has been largely underexplored in medicinal chemistry, we conducted computational similarity and structural analyses to assess its potential for application in drug discovery through fragment replacement.

To ascertain the potential for drug discovery of **2**, this fragment was compared to heterocycles contained in approved drugs from the last decade [16]. The structures of small‐molecule drugs were obtained from the ChemSpider website, and then the scaffolds were generated by cleaving acyclic bonds, resulting in their corresponding cyclic fragments. These fragments consist of contiguous or individual rings, which keeps type of atoms, connection orders, aromaticity information, and exocyclic double bonds, but discarding stereochemistry and charges.

Focusing on heterocyclic fragments, considering their superior properties [17], different heteroaromatic scaffolds were obtained such as benzimidazole, indole, indazole, benzodioxole, benzothiadiazepine, benzothiazole, isoquinoline, and 3,4‐dihydroquinolin‐2(1*H*)‐one. The scaffolds were optimized to the most energetically stable conformation and then aligned with our scaffolds, employing vROCS [18] and EON software to calculate 3D shape, pharmacophoric, and electrostatic (ESP) similarities. In the present analysis, each scaffold was represented by the 30 lowest‐energy conformers before shape and ESP alignment with a single conformer (lowest‐energy) of the template (aripiprazole and/or their target fragment) using vROCS/EON, and the best‐ranked conformation was used for analysis [19, 20]. Based on the obtained similarity scores, the highest similarities were between **2** and **36** (99% of 3D shape, 50% of color and 49% of ESP). The scaffold **36** was generated from the molecule of aripiprazole **40**, which is a third‐generation atypical antipsychotic approved for the treatment of schizophrenia, bipolar disorder, and treatment‐resistant major depression (Scheme 2) [21]. Its principal mechanism of action appears to be associated with partial agonism at the D2 dopamine receptor [21].

### 2.2 Synthesis of Aripiprazole Analog 40

Aiming to validate this strategy, we proposed the replacement of scaffold **36** for **2** in the original drug structure **41**. The synthesis of **40** was carried out by O‐alkylation of **2** using 1,4‐dibromobutane **37** and sodium hydride in DMF at 60 °C. Initial attempts using potassium carbonate gave poor conversion (<30%). Using 3 equivalents of dibromobutane minimized bis‐alkylation while ensuring good conversion to intermediate 3‐(4‐bromobutoxy)‐2,6‐naphthyridine **38** (55% yield) (Scheme 2). The substitution reaction of **38** with 1‐(2,3‐dichlorophenyl)piperazine hydrochloride **39** required optimization to avoid decomposition. Standard conditions (K_2_CO_3_, DMF, 80 °C) gave N‐dealkylation byproducts. Addition of catalytic sodium iodide and lower temperature (60 °C) with extended reaction time (24 h) provided 3‐(4‐(4‐(2,3‐dichlorophenyl)piperazin‐1‐yl)butoxy)‐2,6‐naphthyridine **40** in 61% yield (Scheme 2) on multigram scale [22]. The overall sequence proceeded in two steps from scaffold **2** with 33% overall yield, compared to the six‐step synthesis typically required for aripiprazole.

**SCHEME 2:**
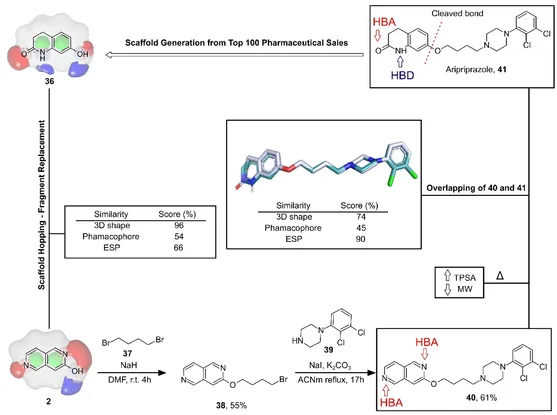
Synthesis of **40**, scaffold hopping by fragment replacement of **36** by **2**. Comparative illustration—the molecular volume is represented by solvent accessible surface (white surface), the pharmacophoric model is represented by rings, H‐bond donors and H‐bond acceptors (green, blue and red spheres, respectively), and the electrostatic potential is represented as negative charged regions and positive ones (red and blue contours, respectively). The original drugs in superimposition figure are presented in gray carbons and substituted ones in pale yellow carbons.

### 2.3 Computational Similarity Analysis and Biological Activity

The new derivative **40** was compared with **41**, presenting 3D shape similarity of 74%, color similarity of 45%, and high ESP similarity of 90%. The high ESP correlation suggests similar interaction patterns with biological targets despite the structural changes. The scaffold replacement results in exchange of 3,4‐dihydroquinolin‐2(1*H*)‐one’s HBD for naphthyridinol’s additional HBA, creating opportunity for different binding modes. The dipole vectors of both molecules align closely (within 15°), suggesting similar orientation preferences in binding sites.

For the replaced structure **40**, calculations indicate the exchange of one hydrogen‐bond donor for a new hydrogen‐bond acceptor at a different position, enabling exploration of new interaction sites [23]. This modification is predicted to increase the topological polar surface area (TPSA) from 44.8 to 54.9 Å^2^ while reducing MW by 21 Da and decreasing lipophilicity (ΔcLog P = −0.8), potentially improving solubility and brain penetration.

To evaluate whether compound **40** could reproduce the biological effects of aripiprazole (**41**), we employed tests commonly used in laboratory animals for detection of antipsychotic activity. The first test assessed the ability of the new compound to prevent or reverse behavioral alterations induced by psychostimulant drugs, such as cocaine [24, 25]. A second test evaluated the potential of the new compound to induce extrapyramidal effects, a common adverse effect associated with classical antipsychotics [24, 26]. The dose of aripiprazole (1 mg/kg) was selected based on published literature showing this dose to be behaviorally active in mice [27], while compound **40**, a new chemical entity with no prior in vivo data, was evaluated across a dose‐range‐finding design (1, 5, 10, and 20 mg/kg) to establish its minimum effective dose in this model.

Treatment with cocaine resulted in a significant increase in locomotor activity compared with the vehicle‐saline group. Pretreatment with aripiprazole **41** (1 mg/kg) inhibited this effect. The two higher doses of compound **40** (10 and 20 mg/kg) also blocked the hyperactivity induced by cocaine (F(6,43) = 9.31, *p* < 0.05, *n* = 6–8 per group) (Figure 6). These data suggest that compound **40** mimics the antipsychotic effect of aripiprazole **41**. The higher effective dose of compound **40** relative to aripiprazole does not necessarily indicate reduced intrinsic potency at the D2 receptor since in vivo effective dose reflects the combined contribution of receptor affinity and pharmacokinetic factors, including absorption, metabolism, protein binding, and brain penetration, and behaviorally active doses of dopaminergic ligands are not always proportional to their in vitro receptor affinity or occupancy [28, 29]. A direct D2 receptor binding or functional assay would be required to determine whether this dose difference reflects reduced receptor affinity, altered pharmacokinetics, or both and is identified here as a priority for follow‐up work.

**FIGURE 6:**
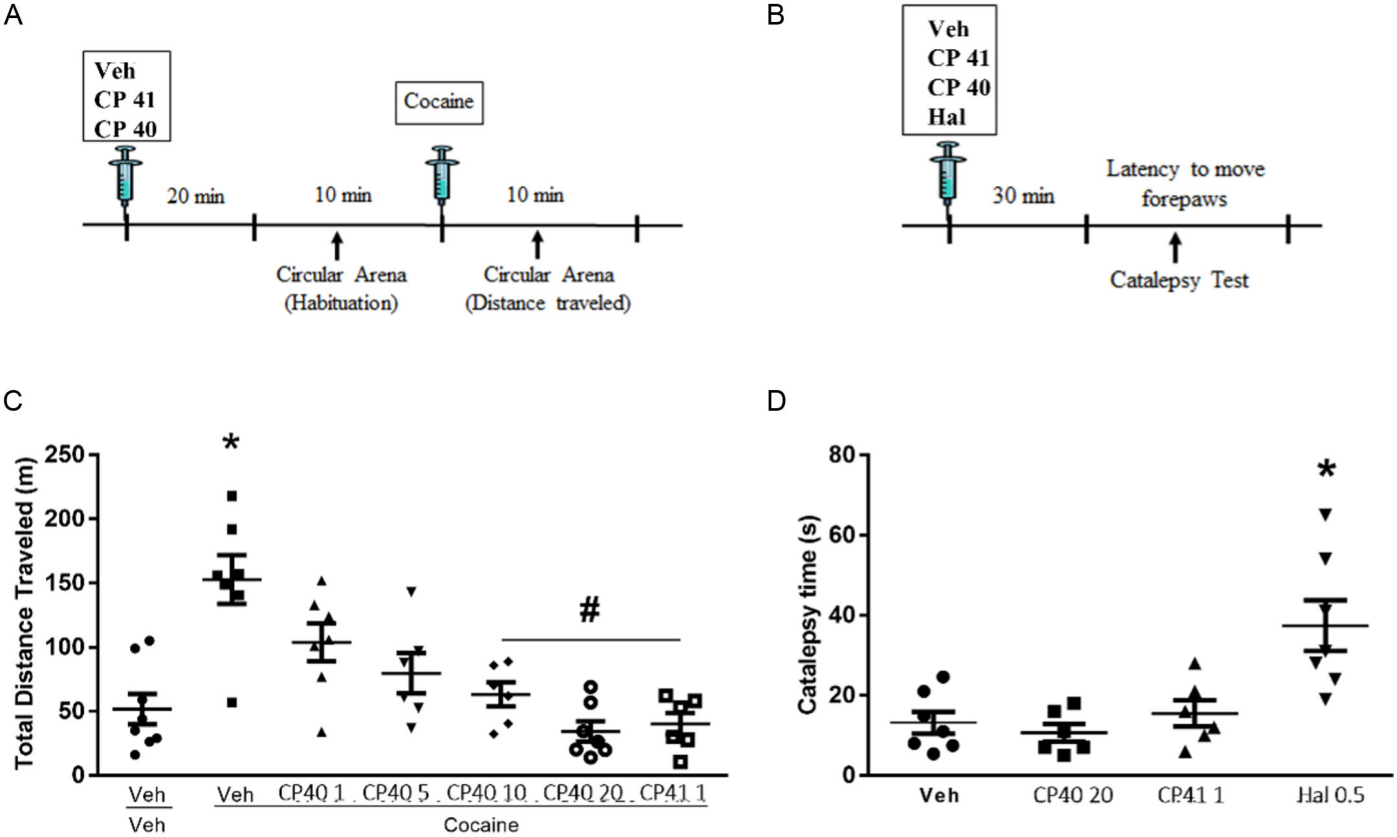
Panels (A,B) show a schematic diagram of the experimental bioassay. Panel (C) demonstrates the effects of compound 40 (CP40; 1, 5, 10, or 20 mg/kg) and aripiprazole (CP41; 1 mg/kg) on cocaine‐induced motor hyperactivity (Coc; 15 mg/kg) in mice (*n* = 6–8 per group). Panel (D) shows the effects of compound 40 (CP40; 20 mg/kg), aripiprazole (CP41; 1 mg/kg), and haloperidol (Hal; 0.5 mg/kg) in the catalepsy test in mice (*n* = 6–7 per group). Data are expressed as mean ± SEM. *p < 0.05 compared with the vehicle–vehicle group; #*p* < 0.05 compared with the vehicle–cocaine group (one‐way ANOVA followed by the Newman–Keuls post hoc test).

The catalepsy test was conducted to evaluate extrapyramidal symptoms [21, 30]. Haloperidol promoted a significant increase in the time spent in immobility, while aripiprazole **41** and compound **40** did not induce catalepsy (F(3,24) = 8.61, *p* < 0.05, *n* = 6–7 per group) (Figure 6). Together, these data suggest that **40** inhibits cocaine hyperlocomotion without promoting motor impairment, sharing the pharmacological properties of aripiprazole **41** and showing potential antipsychotic activity.

## 3 Conclusion

We described the synthesis of 12 promising heterocyclic fragments, **1, 2, 6,** and **8** are underexplored in the literature, and **3**, **4**, **5**, **31–35**, are novel fragments reported for the first time here in this work. These scaffolds were obtained in 3–5 steps with moderate to good yields through streamlined synthetic routes. Several scaffolds fully comply with the “rule of three” criteria for fragments and exhibit favorable three‐dimensionality. The novelty of these heterocycles, particularly their absence from commercial libraries and minimal patent coverage, represents a significant addition to the chemical space available for drug discovery.

The aripiprazole analog **40** was obtained by scaffold‐hopping strategies, specifically by fragment replacement. This compound retained antipsychotic activity without inducing catalepsy, validating both our synthetic approach and the utility of scaffold **2**. Target engagement cannot be directly inferred from behavioral assays, and although computational shape/ESP similarity to aripiprazole suggested similar activity with control, D2 receptor binding and functional assay, should be done in the progress of the work. Furthermore, experimental ADME/PK characterization of compound **40** should validate the compound to further studies. We believe receptor binding/functional assays, a broader behavioral experiments, and experimental ADME/PK profiling as priorities for follow‐up work. These fragments can be applied to multiple drug discovery strategies, including scaffold hopping, to improve the drug‐like properties of the resulting compounds and offers a way to innovate in drug discovery.

## 4 Experimental Section

### 4.1 General Method for Chlorination of Hydroxy‐Pyridazine

Hydroxy‐pyridazine (3 mmol) was added to a round‐bottom flask containing POCl_3_ (7 mL). The reaction was stirred at 100 °C overnight. After cooling, the mixture was concentrated at pressure reduced, and the residue was dissolved in chloroform, washed with water, and the organic phase was dried with sodium sulfate. The solvent was removed under reduced pressure, and the solid residue was purified by silica gel column chromatography (Hexane/Ethyl Acetate 7:3).

“**Caution:** Because phosphoryl chloride (POCl_3_) is highly toxic, volatile, and corrosive, its removal under reduced pressure must be performed strictly within a well‐ventilated fume hood using an appropriate chemical trap to neutralize acidic vapors.”

#### 4.1.1 *Chloropyridazine‐4‐Carbonitrile* (14)

*R*_f_ = 0.4 (DCM/EtOAc 7:3) Yield, (79%), orange oil; ^1^H NMR (300 MHz, CDCl3) *δ* 9.38 (d, *J* = 5.0 Hz, 1H), 7.87 (d, *J* = 5.0 Hz, 1H). ^13^C NMR (75 MHz, CDCl3) *δ* 155.2, 150.2, 131.1, 115.1, 112.4. HRMS‐ (+ESI) *m/z*: [M + H]^+^ calc. for C_5_H_2_ClN_3_, 140,0010; found 140.0008.

#### 4.1.2 *3‐Chloro‐5*,*6‐Dimethylpyridazine‐4‐Carbonitrile* (15)

*R*_f_ = 0.30 (Hex/EtOAc 7:3) Yield, (58%); an off‐white crystalline solid. MP 70–72 °C. ^1^H NMR (400 MHz, CDCl_3_) *δ* (ppm) 2.75 (3H, s), 2.59 (3H, s). ^13^C NMR (101 MHz, CDCl_3_) *δ* 159.7, 153.1, 142.8, 114.7, 112.2, 20.3, 17.9. HRMS‐ (+ESI) *m/z*: [M + H]^+^ calc. for C_7_H_7_ClN_3_^+^: 168,0323, found, 168.0325.

### 4.2 General Method to Obtain Alkylated Products 16–23

To a stirred solution of NaH (289 mg, 7.22 mmol, 60% dispersion in Mineral Oil) in THF (5 mL) was added diethyl malonate (1.16 g, 7.22 mmol) in THF (5 mL) dropwise at 0 °C under N_2_ atmosphere. After the reaction mixture was stirred at 0 °C for 15 min, compound **12, 13** (500 mg, 3.61 mmol) in THF (5 mL) was added. The reaction mixture was heated at reflux for 20 h under N_2_. It was cooled to room temperature and quenched with aq NH_4_Cl (20 mL), and extracted with EtOAc (3 × 20 mL). The organic layer was washed with brine (2 × 10 mL), dried over Na_2_SO_4_, and concentrated. It was purified using column chromatography with ethyl acetate in hexane.

#### 4.2.1 *Diethyl 2‐*(*4‐Cyanopyridazin‐3‐yl*)*Malonate* (16)

(53%): *R*_f_ = 0.4 (Hex/EtOAc 8:2) oil orange oil; ^1^H NMR (300 MHz, CDCl3) *δ* 9.40 (d, *J* = 5.1 Hz, 1H), 7.79 (d, *J* = 5.1 Hz, 1H), 5.40 (s, 1H), 4.34 (q, *J* = 7.1 Hz, 4H), 1.32 (t, *J* = 7.1 Hz, 6H). ^13^C NMR (75 MHz, CDCl3) *δ* 165.2, 150.5, 129.3, 113.6, 63.0, 58.4, 13.9. HRMS‐ (+ESI) *m/z*: [M + H]^+^ calc. for C_12_H_13_N_3_O_4_, 264,0979; found, 264.0979.

#### 4.2.2 *Diethyl 2‐*(*4‐Cyano‐5*,*6‐Methylpyridazin‐3‐Yl*)*Malonate* (17)

*R*_f_ = 0.38 (DCM/EtOAc 7:3); Off‐white oil (53%); ^1^H NMR (400 MHz, CDCl_3_) *δ* 5.31 (s, 1H), 4.36 ( 4H), 2.79 (s, 3H), 2.58 (s, 3H), 1.35 (t, *J* = 7.1 Hz, 6H). ^13^C NMR (101 MHz, CDCl_3_) *δ* 165.5, 159.8, 153.7, 140.1, 114.6, 113.2, 62.7, 58.2, 20.5, 17.5, 13.9. HRMS‐ (+ESI) *m/z*: [M + H]^+^ calc. for C_14_H_18_N_3_O_4_^+^, 292,1292; found, 292.1284.

#### 4.2.3 *Diethyl 2‐*(*3‐Cyanopyridin‐2‐Yl*)*Malonate* (18)

*R*_f_ = 0.37 (Hex/EtOAc 7:3) Yellow oil (57%); ^1^H NMR (400 MHz, CDCl_3_) *δ* 8.72 (dd, *J* = 4.9, 1.8 Hz, 1H), 7.95 (ddd, *J* = 7.9, 5.9, 1.9 Hz, 1H), 7.34 (ddd, *J* = 7.6, 4.9, 2.8 Hz, 1H), 5.18 (s, 1H), 4.32–4.22 (m, 4H), 1.23 (t, *J* = 7.1, 6H).^13^C NMR (101 MHz, CDCl_3_) *δ* 165.8, 156.4, 152.3, 140.51, 122.6, 115.7, 110.7, 62.4, 59.1, 13.9. HRMS‐ (+ESI) *m/z*: [M + H]^+^ calc. for C_13_H_14_N_2_O_4_, 263,1047; found, 263,1023.

#### 4.2.4 *Diethyl 2‐*(*4‐Cyanopyridin‐3‐yl*)*Malonate* (20)

*R*_f_ = 0.35 (Hex/EtOAc 7:3) Off‐white oil (32%); ^1^H NMR (300 MHz, CDCl_3_) *δ* 8.96 (s, 1H), 8.76 (d, *J* = 5.0 Hz, 1H), 7.57 (d, *J* = 5.0 Hz, 1H), 5.03 (s, 1H), 4.39–4.24 (m, 4H), 1.34–1.27 (m, 6H).^13^C NMR (75 MHz, CDCl_3_) *δ* 166.0, 151.6, 149.8, 130.1, 125.5, 121.4, 114.9, 62.8, 54.2, 13.9. HRMS‐ (+ESI) *m/z*: [M + H]^+^ calc. for C_13_H_14_N_2_O_4_, 262,1047; found, 263,1030.

#### 4.2.5 *Diethyl 2‐*(*3‐Cyanopyridin‐4‐yl*)*Malonate* (21)

*R*_f_ = 0.38 (Hex/EtOAc 7:3) Off‐white oil ( 80%); ^1^H NMR (400 MHz, CDCl_3_) *δ* 8.88 (d, *J* = 2.3 Hz, 1H), 8.79 (dd, *J* = 5.2, 2.5 Hz, 1H), 7.67 (dd, *J* = 5.3, 2.1 Hz, 1H), 5.03 (s, 1H), 4.32–4.18 (q, *J* = 7.1 Hz 4H), 1.38–1.20 (t, *J* = 7.1 Hz 6H).^13^C NMR (101 MHz, CDCl_3_) *δ* 165.6, 154.0, 152.1, 144.5, 125.0, 1233, 115.14, 111.1, 62.8, 55.2, 13.9. HRMS‐ (+ESI) *m/z*: [M + H]^+^ calc. for C_13_H_14_N_2_O_4_, 263,1047; found, 263,1023.

#### 4.2.6 *Diethyl 2‐*(*3‐Cyanopyrazin‐2‐yl*)*Malonate* (22)

*R*_f_ = 0.35 (Hex/EtOAc 7:3) Yellow oil (93%);‐ ^1^H NMR (300 MHz, CDCl_3_) *δ* 8.76 (d, *J* = 2.4 Hz, 1H), 8.68 (t, *J* = 2.8 Hz, 1H), 5.25 (s, 1H), 4.36–4.26 (q, *J* = 7.1 Hz 4H), 1.29 (t, *J* = 7.1 Hz, 6H).^13^C NMR (75 MHz, CDCl_3_) *δ* 165.2, 153.1, 146.4, 144.2, 131.2, 114.5, 62.8, 57.6, 13.9. HRMS‐ (+ESI) *m/z*: [M + H]^+^ calc. for C_12_H_13_N_3_O_4_, 264,0979; found, 264,0974.

#### 4.2.7 *Diethyl‐2‐*(*Amino*(*3‐Chloropyridin‐2‐Yl*)*Methylene*)*Malonate* (23)

*R*_f_ = 0.2 (Hex/EtOAc 7:3) **MP‐**71.4–72.0 °C. Light yellow crystalline solid (50%) ^1^H NMR (300 MHz, Acetone) ^1^H NMR (300 MHz, Acetone) *δ* 8.48 (dd, *J* = 4.7, 1.3 Hz, 1H), 7.90 (dd, *J* = 8.2, 1.4 Hz, 1H), 7.45 (dd, *J* = 8.2, 4.7 Hz, 1H), 4.21 (q, *J* = 7.1 Hz, 2H), 3.75 (q, *J* = 7.1 Hz, 2H), 1.28 (t, *J* = 7.1 Hz, 3H), 0.85 (t, *J* = 7.1 Hz, 3H). ^13^C NMR (75 MHz, Acetone) *δ* 168.7, 165.6, 162.6, 153.9, 147.0, 137.0, 129.5, 124.9, 93.0, 59.3, 58.9, 13.8, 13.3. HRMS‐ (+ESI) *m/z*: [M + H]^+^ calc. for C_13_H_15_ClN_2_O_4_, 299,0793; found, 299,0759.

#### 4.2.8 *Diethyl 2‐*(*Amino*(*3‐Chloropyridin‐4‐Yl*)*Methylene*)*Malonate* (24)

**MP‐**122.6–123.2 °C. Off‐white crystalline solid (60%) ^1^H NMR (300 MHz, Acetone) *δ* 8.62 (s, 1H), 8.54 (d, *J* = 4.9 Hz, 1H), 7.36 (dd, *J* = 4.9, 0.4 Hz, 1H), 4.21 (q, *J* = 7.1 Hz, 2H), 3.79 (tt, *J* = 8.0, 4.0 Hz, 2H), 1.27 (t, *J* = 7.1 Hz, 3H), 0.87 (t, *J* = 7.1 Hz, 3H). ^13^C NMR (75 MHz, Acetone) *δ* 168.1, 165.5, 160.1, 149.1, 147.8, 144.2, 129.2, 123.4, 93.6 (s), 59.3, 58.9 13.8, 13.0 (s). HRMS‐ (+ESI) *m/z*: [M + H]^+^ calc. for C_13_H_15_ClN_2_O_4_, 299,0793; found, 299,0779.

### 4.3 General Method for Synthesis of 16–18, 20–23

To a solution of dicarboxylated compound (1.0 g, 3.61 mmol) in DMSO (30 mL) were added H_2_O (1 mL) and LiCl (459 mg, 10.82 mmol), and the resulting mixture was stirred at 100 °C overnight. After the reaction mixture was cooled to room temperature, it was extracted with EtOAc (3 × 20 mL), washed with brine (10 × 2 mL), dried over Na_2_SO_4_, and concentrated. The residue was purified by column chromatography (EtOAc in hexane, 3:7).

#### 4.3.1 *Ethyl 2‐*(*4‐Cyanopyridazin‐3‐yl*)*Acetate* (25)

*R*_f_ = 0.4 (Hex/EtOAc 7:3) (63%), yellow oil; ^1^H NMR (400 MHz, CDCl3) *δ* 9.35 (d, *J* = 5.2 Hz, 1H), 7.74 (d, *J* = 5.2 Hz, 1H), 4.31–4.15 (m, 4H), 1.27 (t, *J* = 7.1 Hz, 3H). ^13^C NMR (101 MHz, CDCl3) *δ* 168.1, 156.5, 150.0, 128.6, 114.5, 113.9, 62.1, 40.8, 14.1. HRMS‐ (+ESI) *m/z*: [M + H]^+^ calc. for C_9_H_9_N_3_O_2,_ 192,0768; found, 191,0767.

#### 4.3.2 *Ethyl 2‐*(*4‐Cyano‐5*,*6‐Dimethylpyridazin‐3‐yl*)*Acetate* (26)

*R*_f_ = 0.5 (DCM/EtOAc 7:3) Off‐white oil (75%) ^1^H NMR (400 MHz, CDCl_3_) *δ* 4.25 (q, *J* = 7.1 Hz, 2H), 4.21 (s, 2H), 2.76 (s, 3H), 2.56 (s, 3H), 1.30 (t, *J* = 7.1 Hz, 3H). ^13^C NMR (101 MHz, CDCl3) *δ* 168.4, 159.2, 154.4, 139.6, 114.5, 113.7, 61.8, 40.7, 20.4, 17.4, 14.1.

#### 4.3.3 *Ethyl 2‐*(*3‐Cyanopyridin‐2‐yl*)*Acetate* (27)

*R*_f_ = 0.5 (Hex/EtOAc 6:4) Light yellow oil (95%); ^1^H NMR (400 MHz, CDCl_3_) *δ* 8.76 (dd, *J* = 4.9, 1.7 Hz, 1H), 7.99 (dd, *J* = 7.9, 1.7 Hz, 1H), 7.37 (dd, *J* = 7.9, 4.9 Hz, 1H), 4.22 (q, *J* = 7.1 Hz, 2H), 4.11 (s, 2H), 1.28 (t, *J* = 7.1 Hz, 3H).^13^C NMR (101 MHz, CDCl_3_) *δ* 168.7, 157.7, 152.4, 140.3, 122.0, 116.2, 110.4, 61.5, 42.7, 14.1. HRMS‐ (+ESI) *m/z*: [M + H]^+^ calc. for C_10_H_10_N_2_O_2_, 191,0815; found, 191,0811.

#### 4.3.4 *Ethyl 2‐*(*3‐Cyanopyridin‐4‐yl*)*Acetate* (28)

*R*_f_ = 0.42 (Hex/EtOAc 6:4) Off white oil (66%); ^1^H NMR (400 MHz, Acetone) *δ* 8.81 (s, 1H), 8.66 (d, *J* = 5.2 Hz, 1H), 7.48 (d, *J* = 5.1 Hz, 1H), 4.08–4.02 (m, 2H), 3.90–3.80 (m, 2H), 1.11 (t, *J* = 7.1 Hz, 3H). ^13^C NMR (101 MHz, Acetone) *δ* 169.3, 153.9, 153.6, 147.8, 126.3, 116.4, 112.1, 62.0, 39.5, 14.4. HRMS (+ESI) *m/z*: [M + H]^+^ calc. for C_10_H_10_N_2_O_2_, 191,0815; found, 191,0813.

#### 4.3.5 *Ethyl 2‐*(*4‐Cyanopyridin‐3‐yl*)*Acetate* (29)

*R*_f_ = 0.4 (Hex/EtOAc 6:4) Off‐white oil (59%); ^1^H NMR (300 MHz, CDCl_3_) *δ* 8.98–8.81 (m, 1H), 8.72 (t, *J* = 11.6 Hz, 1H), 7.41 (d, *J* = 5.2 Hz, 1H), 4.31–4.12 (t, *J* = 7.1 Hz, 2H), 3.87 (s, 2H), 1.33–1.21 (t, *J* = 7.1 Hz, 3H). ^13^C NMR (75 MHz, CDCl_3_) *δ* 168.8, 151.7, 149.1, 131.6, 125.40, 121.5, 115.3, 61.8, 36.9, 14.1. HRMS (+ESI) *m/z*: [M + H]^+^ calc. for C_10_H_10_N_2_O_2_, 191,0815; found, 191,0813.

#### 4.3.6 *Ethyl 2‐*(*3‐Cyanopyrazin‐2‐Yl*)*Acetate* (30)

*R*_f_ = 0.6 (Hex/EtOAc 6:4) Yellow oil (66%); ^1^H NMR (300 MHz, CDCl_3_) *δ* 8.73 (d, *J* = 2.4 Hz, 1H), 8.63 (d, *J* = 2.4 Hz, 1H), 4.22 (q, *J* = 7.1 Hz, 2H), 4.12 (s, 2H), 1.34–1.22 (t, *J* = 7.1 Hz, 3H). ^13^C NMR (75 MHz, CDCl_3_) *δ* 168.1, 154.2, 146.5, 143.7, 131.2, 114.9, 61.9, 41.0, 14.1. HRMS (+ESI) m/z: [M + H]^+^ calc. for C_9_H_9_N_3_O_2,_ 192,0768; found, 191,0772.

### 4.4 Procedure for Synthesis of 3‐Oxo‐5,6‐Dimethyl‐2,3‐Dihydropyridazine‐4‐Carbonitrile (34)

In a screw‐capped reaction tube, a mixture of diacetyl **11** (10 mmol), **10** cyanoacetic hydrazide (10 mmol), and a few drops of acetic acid were refluxed in water (20 mL) overnight. Upon completion of the reaction (as indicated by TLC analysis), the mixture was extracted with EtOAc (3 15 mL). The combined organic layers were washed with brine (15 mL), dried over Na_2_SO_4,_ and concentrated under reduced pressure. The crude material was purified by column chromatography (silica, DCM/EtOAc 7:3). Compound **8** was isolated as a white solid in 58% yield.

*3‐Oxo‐5*,*6‐dimethyl‐2*,*3‐dihydropyridazine‐4‐carbonitrile* (**12**) off‐white crystalline solid (58%). ^1^H NMR (400 MHz, DMSO) *δ* (ppm) 13.40 (1H, s), 2.38 (3H, s), 2.27 (3H, s). HRMS‐ (+ESI) *m/z*: [M + Na]^+^ calc. for C_7_H_7_N_3_ONa^+^, 172,0481; found, 172.0483.

### 4.5 Reductive Cyclization of 16–18, 20−23, 25–30

To a round bottom flask (1 equiv) of **16–18, 20−23, and 25–30,** Pd/C 10% (2 equiv) in AcOH/EtOH (3:1) were added. The mixture was purged with H_2_ and subsequently stirred under H_2_ atmosphere at 50 °C. After TLC analysis indicated full conversion of the starting material, the mixture was filtered over celite and concentrated under vacuum. The mixture was extracted with EtOAc, the combined organic phases washed with brine, dried over Na_2_SO_4_, and concentrated under vacuum. The crude product was purified by column chromatography using MeOH in DCM (3:7).

#### 4.5.1 *5*,*8‐Dihydropyrido*[*4*,*3‐c*]*Pyridazin‐7*(*6H*)*‐One* (4)

*R*_f_ = 0.4 (CH_2_Cl_2_/MeOH 8:2) yellow solid (55%); **MP‐**175.2–175.0 °C. ^1^H NMR (300 MHz, DMSO) *δ* 9.10 (d, *J* = 5.1 Hz, 1H), 8.18 (s, 1H), 7.61 (d, *J* = 5.1 Hz, 1H), 4.42 (s, 2H), 3.80 (s, 2H); ^13^C (75 MHz, DMSO) *δ* 168.9, 156.2, 149.9, 132.1, 123.1, 42.0, 37.5HRMS (+ESI) *m/z*: [M + H]^+^ calc. for C_7_H_8_N_3_O^+^, 150,0662; found, 150,0661.

#### 4.5.2 *3*,*4‐Dimethyl‐5*,*8‐Dihydropyrido*[*4*,*3‐c*]*Pyridazin‐7*(*6H*)*‐One* (5)

*R*_f_ = 0.35 (DCM/EtOAc 6:4) OffWhite oil (62%); ^1^H NMR (400 MHz, DMSO) *δ* 8.13 (s, 1H), 4.40 (s, 2H), 3.70 (s, 2H), 2.58 (s, 3H), 2.20 (s, 3H). ^13^C NMR (101 MHz, DMSO) *δ* 169.2, 158.1, 153.5, 132.5, 129.9, 40.7, 37.2, 20.4, 13.6. HRMS‐ (+ESI) *m/z*: [M + H]^+^ calc. for C_9_H_12_N_3_O^+^, 178,0622; found, 178.0975.

#### 4.5.3 *5*,*8‐Dihydro‐1*,*6‐Naphthyridin‐7*(*6H*)*‐One* (6)

*R*_f_ = 0.33 (CH_2_Cl_2_/MeOH 9:1); Off‐white solid (58%). **MP‐**154.4–155.3 °C. ^1^H NMR (400 MHz, Acetone) *δ* 8.31 (d, *J* = 4.7 Hz, 1H), 7.54 (d, *J* = 7.5 Hz, 1H), 7.21 (s, 1H), 7.11 (dd, *J* = 7.6, 4.9 Hz, 1H), 4.40 (s, 2H), 3.48 (s, 2H).^13^C NMR (101 MHz, Acetone) *δ* 170.8, 153.7, 149.4, 134.1, 127.9, 122.7, 44.1, 40.6. HRMS (+ESI) *m/z*: [M + H]^+^ calc. for C_8_H_8_N_2_O^+^, 149,0710; found, 149,0708.

#### 4.5.4 *1*,*4‐Dihydro‐2*,*7‐Naphthyridin‐3*(*2H*)*‐One* (8)

*R*_f_ = 0.38 (CH_2_Cl_2_/MeOH 9:1); Orange solid (45%). **MP‐**149.1–150.3 °C. ^1^H NMR (300 MHz, Acetone) *δ* 8.48 (d, *J* = 6.0 Hz, 1H), 8.43 (d, *J* = 4.8 Hz, 1H), 7.27–7.19 (m, 1H), 4.56 (s, 2H), 3.55 (s, 2H). HRMS (+ESI) *m/z*: [M + H]^+^ calc. for C_8_H_8_N_2_O^+^:; 149,0710,; found, 149,0718.

#### 4.5.5 *1*,*4‐Dihydro‐2*,*6‐Naphthyridin‐3*(*2H*)*‐One* (1)

*R*_f_ = 0.24 (CH_2_Cl_2_/MeOH 9:1); Off‐white solid (66%). **MP‐**179.6–180.0 °C. ^1^H NMR (300 MHz, Acetone) *δ* 8.45 (s, 1H), 8.42 (d, *J* = 5.0 Hz, 1H), 7.28 (d, *J* = 4.9 Hz, 1H), 4.54 (s, 2H), 3.53 (s, 2H). ^13^C NMR (75 MHz, Acetone) *δ* 169.2, 148.7, 147.5, 140.9, 128.9, 120.9, 44., 33.3. HRMS (+ESI) m/z: [M + H]^+^ calc. for C_8_H_8_N_2_O^+^, 149,0710; found, 149,0706.

#### 4.5.6 *5*,*8‐Dihydropyrido*[*3*,*4‐b*]*Pyrazin‐7*(*6H*)*‐One* (9)

*R*_f_ = 0.21 (CH_2_Cl_2_/MeOH 9:1); Orange solid (51%)‐ **MP‐**178.1–178.5 °C. ^1^H NMR (300 MHz, Acetone) *δ* 8.50 (d, *J* = 2.6 Hz, 1H), 8.47 (d, *J* = 2.5 Hz, 1H), 4.60 (s, 2H), 3.72 (s, 2H). ^13^C NMR (101 MHz, Acetone) *δ* 169.3, 148.9, 148.4, 144.3, 143.3, 46.6, 39.1. HRMS (+ESI) *m/z*: [M + H]^+^ calc. for C_7_H_7_N_3_O^+^, 150.0662; found 150,0661.

#### 4.5.7 *Ethyl 7‐Oxo‐5*,*6*,*7*,*8‐Tetrahydro‐1*,*6‐Naphthyridine‐8‐Carboxylate* (31)

*R*_f_ = 0.35 (CH_2_Cl_2_/MeOH 8:2); Yellow solid (56%). **MP‐**123.8–124.7 °C. ^1^H NMR (300 MHz, Acetone) *δ* 8.49 (d, *J* = 4.8 Hz, 1H), 7.77 (d, *J* = 7.8 Hz, 1H), 7.68 (s, 1H), 7.35 (dd, *J* = 7.7, 4.8 Hz, 1H), 4.78–4.49 (m, 3H), 4.15 (q, *J* = 7.1 Hz, 2H), 1.20 (t, *J* = 7.1 Hz, 3H). ^13^C NMR (75 MHz, Acetone) *δ* 167.8, 166.5, 150.6, 148.6, 134.0, 127.2, 122.6, 61.2, 56.8, 43.2, 13.5. HRMS (+ESI) *m/z*: [M + H]^+^ calc. for C_12_H_12_N_2_O_3_, 221,0921; found, 221,0925.

#### 4.5.8 *Ethyl 3‐Oxo‐1*,*2*,*3*,*4‐Tetrahydro‐2*,*6‐Naphthyridine‐4‐Carboxylate* (32)

*R*_f_ = 0.40 (CH_2_Cl_2_/MeOH 9:1); yellow solid (60%) **MP** 145.2–146.0 °C. ^1^H NMR (300 MHz, CDCl_3_) *δ* 8.54 (s, 1H), 8.51 (d, *J* = 5.1 Hz, 1H), 8.25 (s, 1H), 7.16 (d, *J* = 5.0 Hz, 1H), 4.74 (d, *J* = 16.8 Hz, 1H), 4.55 (s, 1H), 4.39 (dd, *J* = 16.8, 3.9 Hz, 1H), 4.20–4.11 (m, 2H), 1.23 (t, *J* = 7.1 Hz, 3H). ^13^C NMR (75 MHz, CDCl_3_) *δ* 167.7, 167.7, 148.9, 148.8, 140.2, 126.6, 120.5, 62.5, 51.2, 44.5, 13.9. HRMS (+ESI) *m/z*: [M + H]^+^ calc. for C_12_H_12_N_2_O_3_, 221,0921; found, 221,0928.

#### 4.5.9 *Ethyl 3‐Oxo‐1*,*2*,*3*,*4‐Tetrahydro‐2*,*6‐Naphthyridine‐4‐Carboxylate* (33)

*R*_f_ = 0.42 (CH_2_Cl_2_/MeOH 9:1); yellow solid (60%). **MP‐**137.2–137.6 °C. ^1^H NMR (300 MHz, CDCl_3_) *δ* 8.54 (s, 1H), 8.51 (d, *J* = 5.1 Hz, 1H), 8.25 (s, 1H), 7.15 (t, *J* = 4.6 Hz, 1H), 4.74 (d, *J* = 16.8 Hz, 1H), 4.55 (s, 1H), 4.39 (dd, *J* = 16.8, 3.9 Hz, 1H), 4.20–4.11 (m, 2H), 1.27–1.19 (m, 3H). ^13^C NMR (75 MHz, CDCl_3_) *δ* 167.7, 167.7, 148.9, 148.8, 140.2, 126.6, 120.5, 62.5, 51.2, 44.5, 13.9. HRMS (+ESI) *m/z*: [M + H]^+^ calc. for C_12_H_12_N_2_O_3_, 221,0921; found, 221,0922.

#### 4.5.10 *Ethyl‐7‐Oxo‐5*,*6*,*7*,*8‐Tetrahydropyrido*[*3*,*4‐b*]*Pyrazine‐8‐Carboxylate* (34)

*R*_f_ = 0.5 (CH_2_Cl_2_/MeOH 9:1); yellow solid (60%) **MP‐**122.4–122.7 °C ^1^H NMR (300 MHz, CDCl_3_) *δ* 8.86–8.43 (m, 2H), 5.01–4.76 (m, 2H), 4.61 (dd, *J* = 17.2, 3.2 Hz, 1H), 4.25 (ddd, *J* = 14.3, 7.1, 3.6 Hz, 2H), 1.34–1.24 (m, 3H). ^13^C NMR (75 MHz, CDCl_3_) *δ* 167.0, 166.5, 146.7, 145.4, 143.9, 143.7, 62.2, 55.7, 45.9, 14.0. HRMS (+ESI) *m/z*: [M + H]^+^ calc. for C_12_H_13_N_3_O_4_, 222,0873; found, 222,0875.

### 4.6 Procedure for the Synthesis of *1*,*6‐Naphthyridin‐7‐ol* (35)

Activated charcoal (30 mg) was added to a solution of **6** (30 mg) in xylene (10 mL) at 120 °C under an oxygen‐saturated atmosphere. The reaction was kept under stirring for 48 h. The reaction was filtered through celite and washed with MeOH. The solvent was removed under reduced pressure, and the product was purified by flash chromatography (ethyl acetate ‐ methanol, 9:1), resulting in (65%) of a yellow solid.

#### 4.6.1 *1*,*6‐Naphthyridin‐7‐ol* (35)

*R*_f_ = 0.37 (CH_2_Cl_2_/MeOH 9:1); yellow solid (65%) ^1^H NMR (300 MHz, DMSO) *δ* 9.57 (s, 1H), 8.76 (dd, *J* = 4.9, 1.5 Hz, 1H), 8.10 (dd, *J* = 7.6, 1.6 Hz, 1H), 7.58 (dd, *J* = 7.6, 4.9 Hz, 1H), 7.32 (s, 1H). ^13^C NMR (75 MHz, DMSO) *δ* 169.52, 167.41, 165.18, 153.64, 132.19, 125.54, 125.39, 86.08. HRMS (+ESI) *m/z*: [M + H]^+^ calc. for C_8_H_6_N_2_O^+^, 147,0553; found, 147,0550.

Compound **2** were synthesized as described in our previous methodology [7].

### 4.7 Procedure for the Synthesis of *3‐*(*4‐Bromobutoxy*)*‐2*,*6‐Naphthyridine* (38)

To a 5 mL sealed vial was added **2** (233 mg, 1.6 mmol) and NaH (90 mg, 2.4 mmol). The vial was purged with N_2_. Dry DMF (2 mL) was added under N_2_ atmosphere at 0 °C. The mixture was kept under stirring at 0 °C for 10 min. After this time, 1,4‐dibromobutane **37** (0.573 mL, 4.8 mmol) was added to the solution, and the reaction was stirred at room temperature for 17h. After this period, the solution was diluted with saturated Na_2_S_2_O_4,_ and the aqueous phase was extracted with Et_2_O. The organic phase was dried over MgSO_4_. The solvent was removed under reduced pressure, and the product purified by classical column chromatography on silica gel (1:1, EtOAc: Hex).

#### 4.7.1 *3‐*(*4‐Bromobutoxy*)*‐2*,*6‐Naphthyridine* (38)

*R*_f_ = 0.33 (Hex/EtOAc 5:5), pale yellow solid (72%). 95.6–96.1 °C. ^1^H NMR (400 MHz, CDCl_3_) *δ* 9.21 (s, 1H), 9.01 (s, 1H), 8.46 (d, *J* = 5.8 Hz, 1H), 7.68 (d, *J* = 5.8 Hz, 1H), 7.11 (s, 1H), 4.43 (d, *J* = 6.2 Hz, 2H), 3.51 (t, *J* = 6.5 Hz, 2H), 2.12–2.00 (m, 4H). ^13^C NMR (101 MHz, CDCl_3_) *δ* 161.75, 151.31, 150.16, 141.39, 133.76, 126.20, 119.06, 101.06, 66.07, 33.40, 29.70, 29.46, 27.87. ESI‐HRMS (pos): calculated for [C_12_H_14_BrN_2_O]^+^, m/z 281.0284; found, 214,1102.

### 4.8 Procedure for the Synthesis of *3‐*(*4‐*(*4‐*(*2*,*3‐Dichlorophenyl*)*Piperazin‐1‐Yl*)*Butoxy*)*‐2*,*6‐Naphthyridine* (40)

To a round‐bottom flask was added 3‐(4‐bromobutoxy)‐2,6‐naphthyridine 54 (250 mg, 0.89 mmol), NaI (26 mg, 0.178 mmol), K_2_CO_3_ (368 mg, 2.67 mmol), 1‐2,3‐dichlorophenyl) piperazine 55 (284 mg, 1.07 mmol), and acetonitrile (50 mL). The reaction mixture stirred at reflux for 17 h. After this period, the solvent was removed under reduced pressure, the crude residue was solubilized in water (5 mL), and then extracted with CHCl_3_ (3 x 20 mL). The organic phase was dried over MgSO_4_ and concentrated under reduced pressure. The product was purified by flash chromatography (DCM: MeOH, 96:4).

#### 4.8.1 *3‐*(*4‐*(*4‐*(*2*,*3‐Dichlorophenyl*)*Piperazin‐1‐yl*)*Butoxy*)*‐2*,*6‐Naphthyridine* (40)

*R*_f_ = 0.32 (DCM/MeOH 96:4), pale yellow crystals (61%). 106.2–107.0 °C ^1^H NMR (300 MHz, CDCl_3_) *δ* 9.22 (s, 1H), 9.03 (s, 1H), 8.47 (d, *J* = 5.8 Hz, 1H), 7.69 (d, *J* = 5.8 Hz, 1H), 7.14 (dd, *J* = 8.4, 5.7 Hz, 3H), 6.96 (dd, *J* = 6.2, 3.4 Hz, 1H), 4.42 (t, *J* = 6.3 Hz, 2H), 3.09 (s, 4H), 2.69 (s, 4H), 2.62–2,47 (m, 2H), 2.00–1.87 (m, 2H), 1.86–1.71 (m, 2H), 1.26 (dd, *J* = 8.6, 5.7 Hz, 2H). ^13^C NMR (75 MHz, CDCl_3_) *δ* = 161.9, 151.3, 151.2, 150.2, 141.3, 134, 133.7. 127.4, 126.1, 124.5, 119, 118.5, 100.8, 67, 58.2, 53.2, 51.2, 27.2, and 23.4. HRMS (ESI) m/z: [M + H^+^] calculated C_22_H_25_Cl_2_N_4_O^+^, 431.1400; found, 431.1381. Purity 97.7%.

## Author Contributions

The manuscript was written through contributions of all authors. All authors have given approval to the final version of the manuscript. T.O.A.P., P.W., P.E.S.JP., and S.M.G.M. conducted library synthesis. P.H.G. and J.P.G. and S.R.L.J. for biological assays and P.T.T.F.L., G.H.G.T. and V.G.M. for computational studies. Bioin addit was experiments. F.S.E. and W.D. supervised the research. The manuscript was written, reviewed, and edited by T.O.A.P., P.E.S.J., F.S.E., and W.D., and all authors. All authors have approved the final version of the manuscript.

## Funding

This work has been by National Council for Scientific and Technological Development and The World Academy of Science for PhD Scholarship for TOAP (CNPq‐TWAS 163813/2018‐3), São Paulo Research Foundation (FAPESP Grants #2019/15532‐1, #2021/10084‐3, 2021/13237‐5, and #2023/07081‐8), and CAPES for the PhD scholarships.

## Conflicts of Interest

The authors declare no conflict of interest.

## Supporting information

Supplementary Material

## Data Availability

The data that support the findings of this study are available from the corresponding author upon reasonable demand.
