# Supplementary material for: Synthesis of Nitrogen‐Containing Heterocyclic Scaffolds and Applications in Scaffold Hopping: Development of an Aripiprazole Analog
Source: ChemMedChem. 2026 Oct 25;21(20):e70505. doi: 10.1002/cmdc.70505 (PMC13696743; doi:10.1002/cmdc.70505)
Supplement: Supplementary file 1 [file CMDC-21-e70505-s001.pdf]

Supplementary Information

SYNTHESIS OF NITROGEN-CONTAINING HETEROCYCLIC SCAFFOLDS AND APPLICATIONS IN SCAFFOLD HOPPING: DEVELOPMENT OF AN ARIPIPRAZOLE ANALOGUE

Temitayo Omowumi Alegbejo Price[a], Pieterjan Winant[b], Paulo Eliandro da Silva Júnior[a,e], Shaiani Maria Gil de Melo[a], Pedro Henrique Gobira[a], Julia Possamai Gimenes[a], Pedro Tenório Teixeira Fernandes Leite[c], Vinícius Gonçalves Maltarollo[d], Gustavo Henrique Goulart Trossini[d], A. Ganesan[e], Sâmia Regiane Lourenço Joca[a], Wim Dehaen[b], Flavio da Silva Emery[a]*

[a]T. O. Alegbejo Price, P. E da Silva Júnior, S.M.G de Melo P. H Gobira, J. P. Gimenes, S. R. L. Joca, F. S. Emery
Faculty of Pharmaceutical Sciences of Ribeirão Preto,
Department of Pharmaceutical Sciences,
University of Sao Paulo, Ribeirão Preto, 14040-903, Brazil
Email: flavioemery@usp.br
[b] P. Winant, W. Dehaen
Department of Chemistry,
KU Leuven,
Celestijnenlaan 200f-bus 02404, 3001 Leuven, Belgium
[c] P. T. T. F Leite,
Departamento de Produtos Farmacêuticos,
Faculdade de Farmácia,
Universidade Federal de Minas Gerais
[d] V. G. Maltarollo, G. H. G. Trossini
Faculty of Pharmaceutical Sciences,
University of Sao Paulo,
Av. Lineu Prestes, 580, Cid. Universitária, Butantã, São Paulo, 05508-000, Brazil
[e] P. E da Silva Júnior, A. Ganesan
School of Pharmacy,
University of East Anglia,
Norwich Research Park, Norwich, Norfolk NR4

Contents

Computational method: 5

Similarity Study 5

Fragment hopping strategy 8

Calculated relevant pharmaceutical properties 9

Compared to aripriprazole structure; B compared structure 40 and 42. 13

Experimental Bioassay 13

1.Animals 13

2. Drugs 13

3. Behavioural test 14

3.1 Locomotor Activity Assessment 14

3.2 Catalepsy test 14

References 14

NMR Spectra 15

^{1}H NMR (300 MHz, DMSO-d6) spectrum of 3-Oxo-5,6-dimethyl-2,3-dihydropyridazine-4-carbonitrile (12) 15

^{13}C NMR (75 MHz, DMSO-d6) spectrum of 3-Oxo-5,6-dimethyl-2,3-dihydropyridazine-4-carbonitrile (12) 15

^{1}H NMR (300 MHz, $CDCl_3$) spectrum of Chloropyridazine-4-carbonitrile (14) 16

^{1}H NMR (75 MHz, $CDCl_3$) spectrum of Chloropyridazine-4-carbonitrile (14) 16

^{1}H NMR (300 MHz, $CDCl_3$) spectrum of 3-Chloro-5,6-dimethylpyridazine-4-carbonitrile (15) 17

^{13}C NMR (75 MHz, $CDCl_3$) spectrum of 3-Chloro-5,6-dimethylpyridazine-4-carbonitrile (15) 17

^{1}H NMR (300 MHz, $CDCl_3$) spectrum of Diethyl 2-(4-cyanopyridazin-3-yl) malonate (16) 18

^{1}H NMR (75 MHz, $CDCl_3$) spectrum of Diethyl 2-(4-cyanopyridazin-3-yl) malonate (16) 18

^{1}H NMR (300 MHz, $CDCl_3$) spectrum of Diethyl 2-(4-cyano-5,6-methylpyridazin-3-yl) malonate (17) 19

^{1}H NMR (300 MHz, $CDCl_3$) spectrum of Diethyl 2-(4-cyano-5,6-methylpyridazin-3-yl) malonate (17) 19

^{1}H NMR (300 MHz, $CDCl_3$) spectrum of Diethyl 2-(3-cyanopyridin-2-yl)malonate (18) ... 20

^{1}H NMR (75 MHz, $CDCl_3$) spectrum of Diethyl 2-(3-cyanopyridin-2-yl)malonate (18) 20

^{1}H NMR (300 MHz, $CDCl_3$) spectrum of Diethyl 2-(4-cyanopyridin-3-yl)malonate (20) ... 21

^{1}H NMR (75 MHz, $CDCl_3$) spectrum of Diethyl 2-(4-cyanopyridin-3-yl)malonate (20) 21

^{1}H NMR (300 MHz, $CDCl_3$) spectrum of Diethyl 2-(3-cyanopyridin-4-yl)malonate (21) ... 22

^{1}H NMR (75 MHz, $CDCl_3$) spectrum of Diethyl 2-(3-cyanopyridin-4-yl)malonate (21) 22

^{1}H NMR (300 MHz, $CDCl_3$) spectrum of Diethyl 2-(3-cyanopyrazin-2-yl)malonate (22) .. 23

^{1}H NMR (75 MHz, $CDCl_3$) spectrum of Diethyl 2-(3-cyanopyrazin-2-yl)malonate (22) 23

^{1}H NMR (300 MHz, Acetone) spectrum of Diethyl-2-(amino(3-chloropyridin-2-yl)methylene)malonate (23) 24

^{13}C NMR (75 MHz, Acetone) spectrum of Diethyl-2-(amino(3-chloropyridin-2-yl)methylene)malonate (23) 24

^{1}H NMR (300 MHz, Acetone) spectrum of Diethyl 2-(amino(3-chloropyridin-4-yl)methylene)malonate (24) 25

^{13}C NMR (75 MHz, Acetone) spectrum of Diethyl 2-(amino(3-chloropyridin-4-yl)methylene)malonate (24) 25

^{1}H NMR (300 MHz, $CDCl_3$) spectrum of Ethyl 2-(4-cyanopyridazin-3-yl) acetate (25) 26

^{13}C NMR (75 MHz, $CDCl_3$) spectrum of Ethyl 2-(4-cyanopyridazin-3-yl) acetate (25) 26

^{1}H NMR (300 MHz, $CDCl_3$) spectrum of Ethyl 2-(4-cyano-5,6-dimethylpyridazin-3-yl)acetate (26) 27

^{1}H NMR (75 MHz, $CDCl_3$) spectrum of Ethyl 2-(4-cyano-5,6-dimethylpyridazin-3-yl)acetate (26) 27

^{1}H NMR (300 MHz, $CDCl_3$) spectrum of Ethyl 2-(3-cyanopyridin-2-yl)acetate (27) 28

^{1}H NMR (300 MHz, $CDCl_3$) spectrum of Ethyl 2-(3-cyanopyridin-2-yl)acetate (27) 28

^{1}H NMR (300 MHz, $(CD_3)_2CO$) spectrum of Ethyl 2-(3-cyanopyridin-4-yl)acetate (28) 29

^{1}H NMR (75 MHz, $(CD_3)_2CO$) spectrum of Ethyl 2-(3-cyanopyridin-4-yl)acetate (28) 29

^{1}H NMR (300 MHz, $CDCl_3$) spectrum of Ethyl 2-(4-cyanopyridin-3-yl)acetate (29) 30

^{13}C NMR (75 MHz, $CDCl_3$) spectrum of Ethyl 2-(4-cyanopyridin-3-yl)acetate (29) 30

^{1}H NMR (300 MHz, $CDCl_3$) spectrum of Ethyl 2-(3-cyanopyrazin-2-yl)acetate 31

^{1}H NMR (75 MHz, $CDCl_3$) spectrum of Ethyl 2-(3-cyanopyrazin-2-yl)acetate 31

^{1}H NMR (300 MHz, DMSO-d6) spectrum of 5,8-dihydropyrido[4,3-c] pyridazin-7(6H)-one (4) 32

^{13}C NMR (75 MHz, DMSO-d6) spectrum of 5,8-dihydropyrido[4,3-c] pyridazin-7(6H)-one (4) 32

^{1}H NMR (300 MHz, (DMSO) spectrum of 3,4-dimethyl-5,8-dihydropyrido[4,3-c]pyridazin-7(6H)-one (5) 33

^{13}C NMR (75 MHz, (DMSO) spectrum of 3,4-dimethyl-5,8-dihydropyrido[4,3-c]pyridazin-7(6H)-one (5) 33

^{1}H NMR (300 MHz, $(CD_3)_2CO$) spectrum of 5,8-dihydro-1,6-naphthyridin-7(6H)-one (6) 34

^{13}C NMR (75 MHz, $(CD_3)_2CO$) spectrum of 5,8-dihydro-1,6-naphthyridin-7(6H)-one (6) 34

^{1}H NMR (300 MHz, $(CD_3)_2CO$) spectrum of 1,4-dihydro-2,6-naphthyridin-3(2H)-one (1) 35

*^{1}H NMR (75 MHz, $(CD_3)_2CO$) spectrum of 1,4-dihydro-2,6-naphthyridin-3(2H)-one (1)*35

^{1}H NMR (300 MHz, $(CD_3)_2CO$) spectrum of 1,4-dihydro-2,7-naphthyridin-3(2H)-one (8) 36

^{1}H NMR (300 MHz, $(CD_3)_2CO$) spectrum of 5,8-dihydropyrido[3,4-b]pyrazin-7(6H)-one(9) 36

^{13}C NMR (75 MHz, $(CD_3)_2$ CO) spectrum of 5,8-dihydropyrido[3,4-b]pyrazin-7(6H)-one(9) 37
^{1}H NMR (300 MHz, $(CD_3)_2$ CO) spectrum of ethyl 7-oxo-5,6,7,8-tetrahydro-1,6-naphthyridine-8-carboxylate (31) 37
^{13}C NMR (75 MHz, $(CD_3)_2$ CO) spectrum of ethyl 7-oxo-5,6,7,8-tetrahydro-1,6-naphthyridine-8-carboxylate (31) 38
^{1}H NMR (300 MHz, $(CD_3)_2$ CO) spectrum of ethyl 3-oxo-1,2,3,4-tetrahydro-2,6-naphthyridine-4-carboxylate (32) 38
^{13}C NMR (75 MHz, $(CD_3)_2$ CO) spectrum of ethyl 3-oxo-1,2,3,4-tetrahydro-2,6-naphthyridine-4-carboxylate (32) 39
.... 39
^{1}H NMR (75 MHz, (CD 3)2 CO) spectrum of ethyl 3-oxo-1,2,3,4-tetrahydro-2,7-naphthyridine-4-carboxylate 33 39
^{13}C NMR (75 MHz, $(CD_3)_2$ CO) spectrum of ethyl 3-oxo-1,2,3,4-tetrahydro-2,7-naphthyridine-4-carboxylate (33) 40
^{1}H NMR (300 MHz, $(CD_3)_2$ CO) spectrum of Ethyl-7-oxo-5,6,7,8-tetrahydropyrido[3,4-b]pyrazine-8-carboxylate (34) 40
^{13}C NMR (75 MHz, $(CD_3)_2$ CO) spectrum of Ethyl-7-oxo-5,6,7,8-tetrahydropyrido[3,4-b]pyrazine-8-carboxylate (34) 41
^{1}H NMR (300 MHz, DMSO) spectrum of 1,6-naphthyridin-7-ol (35) 41
^{13}C NMR (75 MHz, DMSO) spectrum of 1,6-naphthyridin-7-ol 42
^{1}H-NMR – 400 MHz in DMSO-d6: 2,6-naphthyridin-3-ol (2) 42
^{13}C NMR - 101 MHz in DMSO-d6: 2,6-naphthyridin-3-ol (2) 43
^{1}H-NMR – 300 MHz in $CDCl_3$: 3-(4-bromobutoxy)-2,6-naphthyridine (38) 43
^{13}C-NMR – 75 MHz in $CDCl_3$: 3-(4-bromobutoxy)-2,6-naphthyridine (38) 44
^{1}H NMR – 300 MHz in $CDCl_3$: 3-(4-(4-(2,3-dichlorophenyl)piperazin-1-yl)butoxy)-2,6-naphthyridine (40) 44
^{13}C NMR – 75 MHz in $CDCl_3$: 3-(4-(4-(2,3-dichlorophenyl)piperazin-1-yl)butoxy)-2,6-naphthyridine (40) 45
Purity of Most Active compounds 46

Computational method:

Similarity Study

Initially, the 3D structures of studied fragments were constructed with ChemBioDraw and after conformational analysis calculations were performed with OMEGA 2.5.1.4 software in order to generate the most stable conformation of each fragment. The OMEGA conformational analysis uses the Merck molecular force field (MMFF) to calculate the conformational parameters and energies.

In the second step of our computational study, we aligned the fragments **43-90** with fragments **2**, with vROCS software[12]. The alignments were performed with 3D shape and color superimposition of compounds and scoring by a combo of its Tanimoto similarities. After the generation of three alignments, we calculated the electrostatic similarity of between compounds **43-90** and fragment **2**, employing the pre-aligned datasets with EON software. The atomic charges of compounds were calculated with MMFF implemented in EON software.

All similarities (shape, color and electrostatic) were calculated by Tanimoto similarity (Equation 1) that ranges between 0 (completely different structures) and 1 (identical structures). And, finally, the figures of shape, color and electrostatic potential of compounds as well the superimposition of original and substituted drugs were generated using VIDA software.

$$Sim_{Tanimoto}(A,B) = \frac{AB}{|A|+|B|-|AB|} = \frac{AB}{A+B+AB} \quad \textbf{(1)}$$

After alignment between all 48 fragments to each in-house scaffolds, we obtained the Shape, Color and ESP Tanimoto similarity scores that are shown by Supplementary Table 1 and Figure S1.

Table S1. Shape, Color and ESP scores (0, red, and 1, green) obtained from vROCS and EON calculations.

Fragment	2		
	Shape	Color	ESP
**43**	0.772	0.334	0.335
**44**	0.850	0.29	0.000
**45**	0.618	0.241	0.022
**46**	0.922	0.378	0.164

**47**	0.856	0.330	0.045
**48**	0.787	0.207	-0.010
**49**	0.933	0.355	0.166
**50**	0.807	0.322	0.005
**51**	0.712	0.208	-0.005
**52**	0.904	0.438	0.604
**53**	0.604	0.263	0.398
**54**	0.854	0.28	0.112
**55**	0.772	0.144	-0.071
**56**	0.931	0.373	0.012
**57**	0.911	0.349	-0.105
**58**	0.988	0.568	0.442
**59**	0.839	0.35	0.496
**60**	0.998	0.579	0.357
**61**	0.785	0.244	0.049
**62**	0.919	0.401	-0.075
**63**	0.639	0.289	0.168
**64**	0.855	0.253	-0.034
**65**	0.879	0.383	0.269
**66**	0.961	0.428	0.201
**67**	0.820	0.291	-0.025
**68**	0.951	0.360	0.112
**69**	0.933	0.315	-0.027
**70**	0.958	0.428	0.203
**71**	0.929	0.394	0.103
**71**	0.705	0.226	0.099
**73**	0.621	0.251	0.268
**74**	0.572	0.43	0.276
**75**	0.622	0.233	0.318
**76**	0.625	0.286	0.263
**77**	0.578	0.286	0.140
**78**	0.623	0.286	0.244
**79**	0.621	0.283	0.218
**80**	0.620	0.183	0.006
**81**	0.717	0.253	0.306
**82**	0.619	0.193	0.037
**83**	0.720	0.288	0.396
**84**	0.374	0.143	0.161
**85**	0.644	0.144	0.068
**86**	0.704	0.288	0.231
**87**	0.817	0.324	0.104
**88**	0.887	0.280	0.223
**89**	0.936	0.282	0.040
**90**	0.765	0.336	0.004

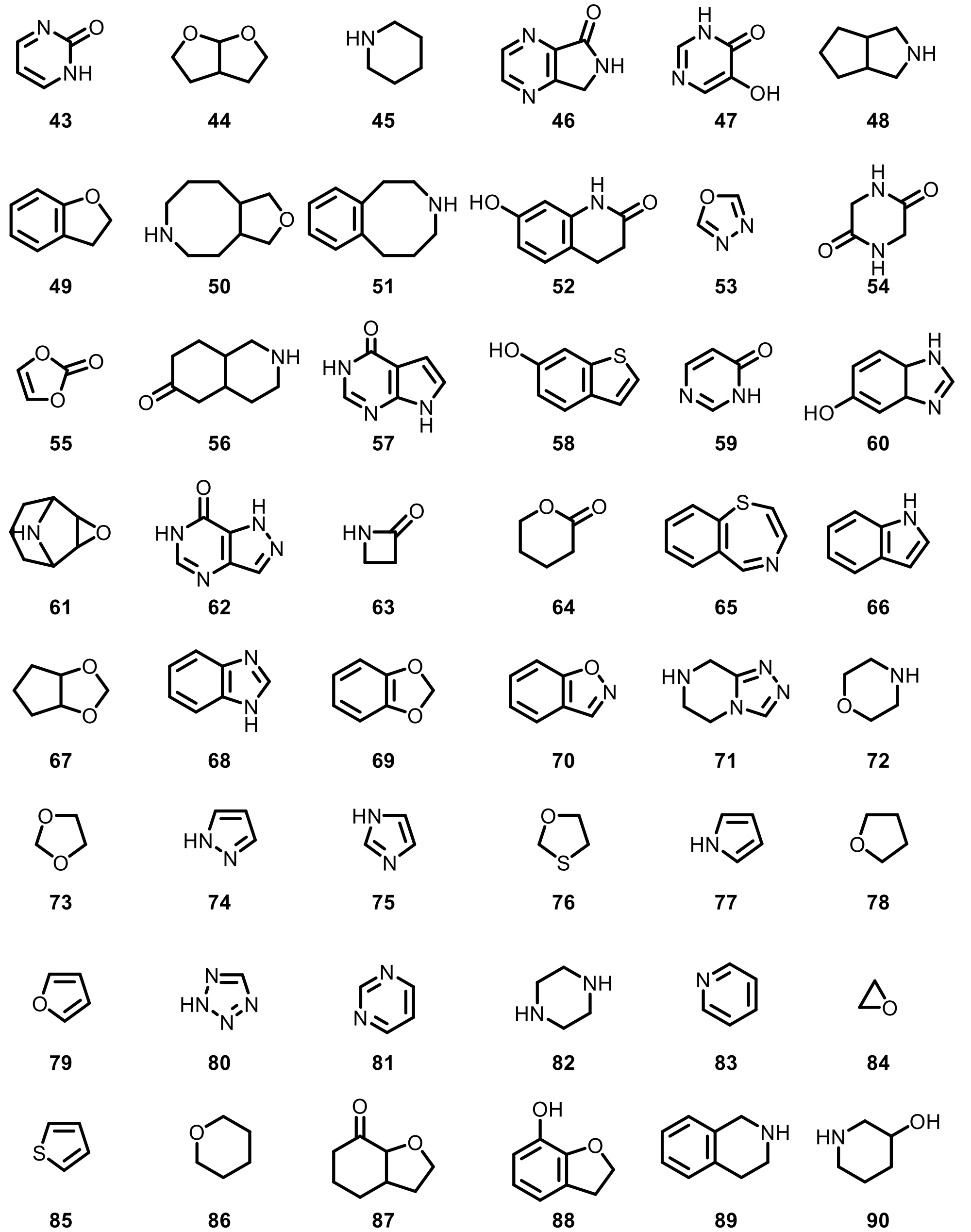

Figure S1. Distribution of shape and color similarities of 48 fragments to our three proposed fragments.

Analyzing statistically the calculated similarities indices, we could note that the shape similarities range from 32% to 99% while color similarities range from 13% to 67% and ESP similarity range from 0 to 75% (Supplementary Tables 1 and 2). The average values of similarity are also higher for shape similarities than color and ESP. Indeed, the maximum values of color and ESP similarities are equals to 56.5% / 48.7%, for fragment **2** respectively. Therefore, is clear that the fragments have higher shape similarity than the others two. From these data, is possible to note that the substitution of drugs fragments for in-house ones is a

good strategy to mimic ligand-receptor complementarity and, just in some cases, to mimic chemical interactions (color and ESP similarity).

Fragment hopping strategy

The replacement of **36** in the structure of aripiprazole was made using the fragments **2**

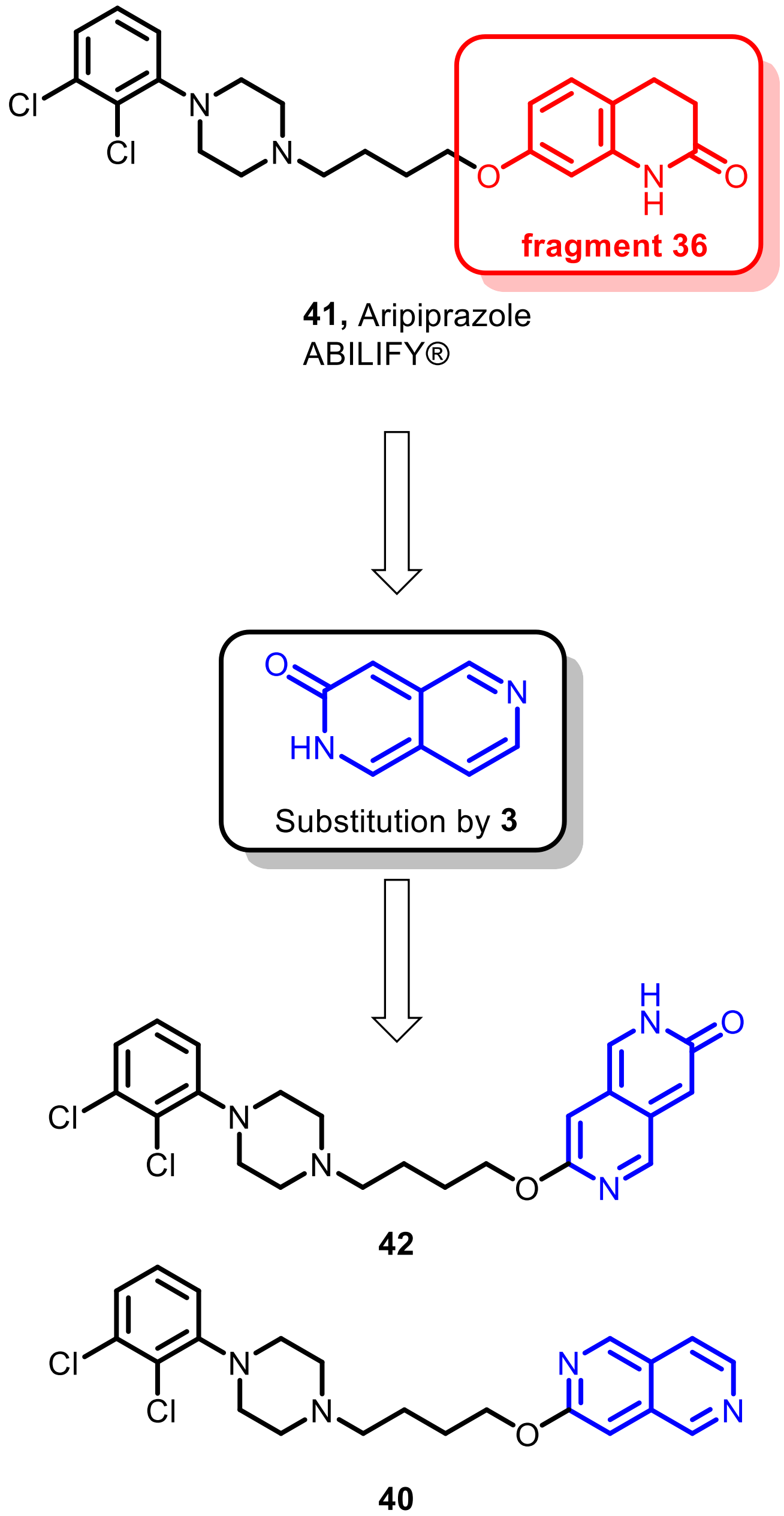

Figure S2. The substitution on aripiprazole structure by **2**.

We generated 2 substituted structures to each of our scaffolds replaced in 2 different positions, then the compounds were aligned by shape and color with the respective drug using

vROCS. It is possible to notice that all the replacements keep average shape similarity of 73.4% (63.8%-92.7% to the original drug providing complementarity to respective target, also relative average color similarity of 43.5% (35.8%-57.8%). Also the ESP similarity between them was calculated using EON, interestingly the replaced structures presented high ESP similarity to **2** (average similarity of 86,95%) (Table S3).

Table S2. Shape, Color and ESP scores obtained from vROCS and EON calculations.

Structure	*Shape Similarity*	*Color Similarity*	*ESP Similarity*
**40**	0.735	0.454	0.899
**42**	0.656	0.398	0.84

Calculated relevant pharmaceutical properties

The structures of small molecule drugs were obtained in ChemSpider website (www.chemspider.com), then decomposed into their corresponding cyclic fragment using ChemBioDraw 13.0 PerkinElmer Informatics program. These fragments consists on contiguous or individual rings, which keeps type of atoms, connections orders, aromaticity information and exocyclic double bonds, but discarding stereochemistry and charges. Focusing on heterocyclic fragments were obtained 47 heterocyclic fragments represented below (Figure 2).

We calculate structural properties of all heterocyclic systems described in this work (**2** and drug fragments **43-90**) such as molecular weight (MW), xlogP, hydrogen bonds acceptor (HBA), hydrogen bond donor (HBD), number of rotatable bonds, topological polar surface area (TPSA) and carbon sp^3 fraction (Fsp^3). These properties were calculated employing the Vortex software from Vortex Dotmatics. (Supplementary Table 4).

Table S3. Relevant pharmaceutical properties and its proposed variation of the structures **2** and drug fragments **43-90**.

Fragment	*MW*	*XLogP*	*HBA*	*HBD*	*TPSA*	*RotBond*	*fSP3*
**2**	146.05	-0.35	3	1	41.5	0	0
**Fragment**	**MW**	**XLogP**	**HBA**	**HBD**	**TPSA**	**RotBond**	**fSP3**
**43**	96.03	-0.57	3	1	41.5	0	0
**44**	114.07	0.88	2	0	18.5	0	1
**45**	85.09	0.93	1	1	12	0	1
**46**	135.04	-1.71	4	1	54.9	0	0.167
**47**	112.03	-1.45	4	2	61.7	1	0

**48**	111.10	1.13	1	1	12	0	1
**49**	120.06	1.79	1	0	9.2	0	0.25
**50**	155.13	1.02	2	1	21.3	0	1
**51**	161.12	2.15	1	1	12	0	0.455
**52**	147.07	1.51	2	1	29.1	0	0.222
**53**	87.03	-0.56	3	1	38.3	0	0.667
**54**	70.02	0.51	3	0	39.2	0	0
**55**	114.04	-1.68	4	2	58.2	0	0.5
**56**	86.00	0.27	3	0	35.5	0	0
**57**	153.12	1.16	2	1	29.1	0	0.889
**58**	135.04	0.32	4	2	57.3	0	0
**59**	150.01	3.04	1	1	48.5	1	0
**60**	96.03	-0.7	3	1	41.5	0	0
**61**	134.05	1.79	3	2	48.9	1	0
**62**	125.08	0.35	2	1	24.6	0	1
**63**	136.04	-0.13	5	2	70.1	0	0
**64**	71.04	-0.39	2	1	29.1	0	0.667
**65**	100.05	1.07	2	0	26.3	0	0.8
**66**	161.03	2.1	1	0	37.7	0	0
**67**	117.06	2.62	1	1	15.8	0	0
**68**	114.07	1.06	2	0	18.5	0	1
**69**	118.05	2.16	2	1	28.7	0	0
**70**	122.04	1.63	2	0	18.5	0	0.143
**71**	119.04	1.49	2	0	26.3	0	0
**71**	124.07	0.22	4	1	42.7	0	0.6
**73**	87.07	-0.52	2	1	21.3	0	1
**74**	74.04	-0.06	2	0	18.5	0	1
**75**	68.04	1.24	2	1	28.7	0	0
**76**	90.01	0.62	1	0	34.5	0	1
**77**	67.04	1.19	1	1	15.8	0	0
**78**	72.06	0.79	1	0	9.2	0	1
**79**	68.03	1.43	1	0	13.4	0	0
**80**	70.03	-0.11	4	1	54.5	0	0

**81**	80.04	0.06	2	0	25.8	0	0
**82**	86.08	-0.84	2	2	24.1	0	1
**83**	79.04	0.65	1	0	12.9	0	0
**84**	44.03	-0.11	1	0	12.5	0	1
**85**	84.00	1.99	0	0	28.2	0	0
**86**	86.07	1.24	1	0	9.2	0	1
**87**	140.08	1.25	2	0	26.3	0	0.875
**88**	136.05	1.42	2	1	29.5	1	0.25
**89**	133.09	1.6	1	1	12	0	0.333
**90**	101.08	-0.24	2	2	32.3	1	1

Moreover we calculated the variation of molecular properties such as MW, hydrogen bonds (HBA and HBD) and TPSA, between **2** and the drug fragments **43-90**(Supplementary Table 5).

Table S4. Proposed properties variation between structures **2** and the drug fragments **43-90**.

Fragment	Δ ***MW***	Δ ***HBA***	Δ ***HBD***	Δ ***TPSA***
	**2**	**2**	**2**	**2**
**43**	50.02	0	0	0
**44**	31.98	1	1	23
**45**	60.96	2	0	29.5
**46**	11.00	-1	0	-13.4
**47**	34.02	-1	-1	-20.2
**48**	34.94	2	0	29.5
**49**	25.99	2	1	32.3
**50**	-9.08	1	0	20.2
**51**	-15.07	2	0	29.5
**52**	-1.02	1	0	12.4
**53**	59.02	0	0	3.2
**54**	76.03	0	1	2.3
**55**	32.01	-1	-1	-16.7

**56**	60.05	0	1	6
**57**	-7.07	1	0	12.4
**58**	11.00	-1	-1	-15.8
**59**	-3.97	2	0	-7
**60**	50.02	0	0	0
**61**	12.00	0	-1	-7.4
**62**	20.96	1	0	16.9
**63**	10.01	-2	-1	-28.6
**64**	75.01	1	0	12.4
**65**	46.00	1	1	15.2
**66**	-14.98	2	1	3.8
**67**	28.99	2	0	25.7
**68**	31.98	1	1	23
**69**	27.99	1	0	12.8
**70**	24.01	1	1	23
**71**	27.01	1	1	15.2
**71**	21.97	-1	0	-1.2
**73**	58.98	1	0	20.2
**74**	72.01	1	1	23
**75**	78.01	1	0	12.8
**76**	56.03	2	1	7
**77**	79.01	2	0	25.7
**78**	73.99	2	1	32.3
**79**	78.02	2	1	28.1
**80**	76.02	-1	0	-13
**81**	66.01	1	1	15.7
**82**	59.96	1	-1	17.4
**83**	67.01	2	1	28.6
**84**	102.02	2	1	29
**85**	62.04	3	1	13.3
**86**	59.97	2	1	32.3
**87**	5.96	1	1	15.2

**88**	10.00	1	0	12
**89**	12.96	2	0	29.5
**90**	44.96	1	-1	9.2

After to calculate the properties and its variations, we conducted the same calculations using Vortex to validate the proposed contribution of each one of scaffolds 1, 2 and 3. To this end we used the same strategy of modification approached in the similarity study. We substituted **14** in the structure of aripiprazole by fragments **2**, then the same properties and variations was calculated (Supplementary Table 6).

Table S6. Validation of the proposed properties variation.

**Drug Properties**								
**Drug**	MW		HBA		HBD		TPSA	
**Aripriprazole**	447.148		6		1		96.3	
**Validation calculations**								
**Structure**	MW	Δ MW	HBA	Δ HBA	HBD	Δ HBD	TPSA	Δ TPSA
**40**	435.1228	-12.0252	7	1	2	1	69.2	24.4
**42**	421.1436	-26.0045	6	0	2	1	52.1	7.3

Compared to aripriprazole structure; B compared structure 40 and 42.

Experimental Bioassay

1.Animals

Male Swiss mice weighing 25–30 g were obtained from the animal facility of the University of São Paulo, Ribeirão Preto campus. Animals were group-housed (5 per cage) in a temperature-controlled room maintained at 25 °C under a 12 h light/dark cycle (lights on at 7:00 a.m.), with food and tap water available ad libitum. All experiments were conducted in accordance with the ethical principles established by the Brazilian National Council for the Control of Animal Experimentation (CONCEA), and all efforts were made to minimize animal suffering. All procedures were approved by the Animal Ethics Committee of the School of Pharmaceutical Sciences of Ribeirão Preto (protocol no. 19.1.1156.60.8).

2. Drugs

Aripiprazole (CP41, 1 mg/kg; Cayman Chemical, USA) used in the present experiments was dissolved in physiological saline containing 5% Tween 80 and 5% DMSO. The same vehicle was used to dissolve compound 40 (1, 5, 10, or 20 mg/kg). Haloperidol (0.5

mg/kg; Teuto, Brazil) and cocaine hydrochloride (15 mg/kg; Merck, Germany) were dissolved in saline. The doses used in this study were selected based on previous reports[1-2].

3. Behavioural test

3.1 Locomotor Activity Assessment

The locomotor activity assessment followed previously established procedures [3]. Briefly, before exposure to the circular arena (40 cm in diameter), animals received an intraperitoneal injection of vehicle, compound 40 (1, 5, 10, or 20 mg/kg), or aripiprazole (1 mg/kg). Twenty minutes after pretreatment, the animals were pre-exposed to the arena for 10 min. Following this habituation phase, each mouse received an injection of either saline or cocaine (15 mg/kg) and was immediately returned to the arena. Locomotor activity was assessed as the total distance traveled over a 10 min period using the AnyMaze software (Stoelting Co., USA).

3.2 Catalepsy test

The catalepsy test was conducted as previously described [1]. Briefly, mice received intraperitoneal injections of aripiprazole (1 mg/kg), compound 40 (20 mg/kg), or haloperidol (0.5 mg/kg). Thirty minutes later, the animals were positioned with their hind paws on a bench and their forepaws placed on a glass bar (0.5 cm in diameter) elevated 5 cm above the floor. The latency for the animal to remove both forepaws from the bar or to climb down was recorded

References

1. Silva Junior, P. E.; Rezende, L. C. D.; Gimenes, J. P.; Maltarollo, V. G.; Dale, J.; Trossini, G. H. G.; Emery, F. S.; Ganesan, A., *Rsc Advances* **2016,** *6* (27), 22777-22780.

2. Brimble, M. A.; Brimble, M. T.; Hodges, R.; Lane, G. A., *Australian Journal of Chemistry* **1988,** *41* (10), 1583-1590.

3. Campiani, G.; Morelli, E.; Gemma, S.; Nacci, V.; Butini, S.; Hamon, M.; Novellino, E.; Greco, G.; Cagnotto, A.; Goegan, M.; Cervo, L.; Dalla Valle, F.; Fracasso, C.; Caccia, S.; Mennini, T., *Journal of Medicinal Chemistry* **1999,** *42* (21), 4362-4379.

NMR Spectra

^{1}H NMR (300 MHz, DMSO-d6) spectrum of 3-Oxo-5,6-dimethyl-2,3-dihydropyridazine-4-carbonitrile (12)

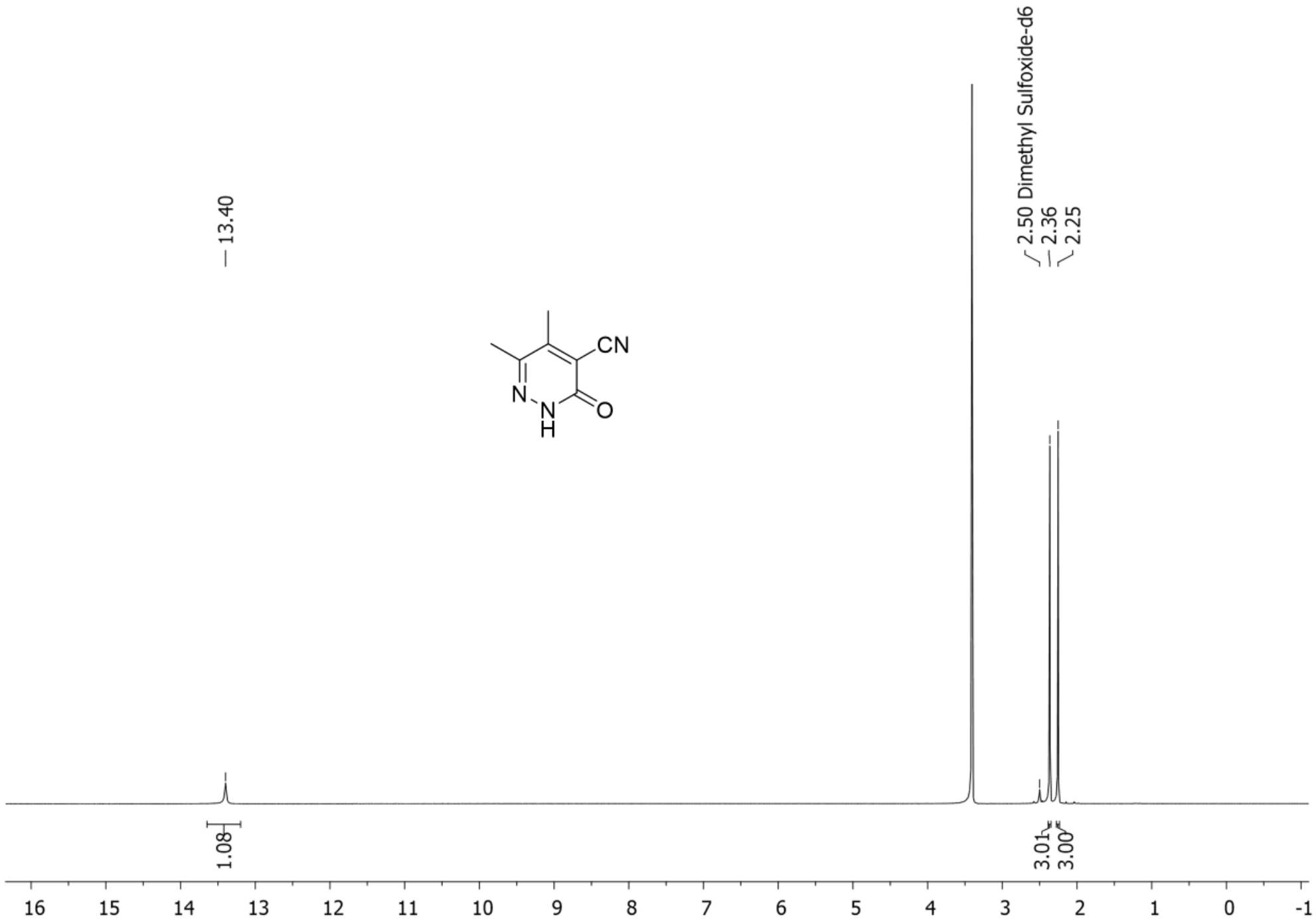

^{13}C NMR (75 MHz, DMSO-d6) spectrum of 3-Oxo-5,6-dimethyl-2,3-dihydropyridazine-4-carbonitrile (12)

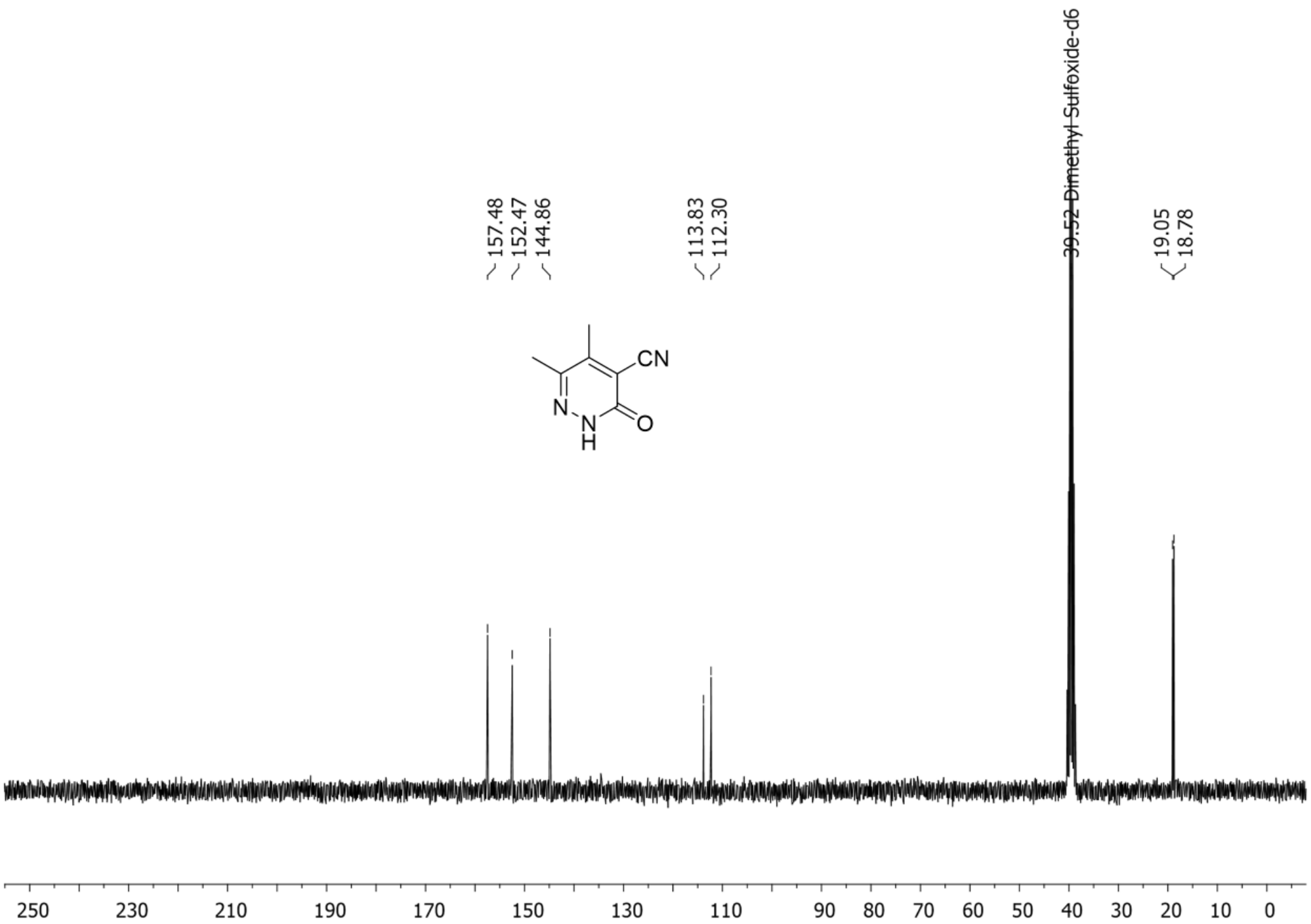

^{1}H NMR (300 MHz, $CDCl_3$) spectrum of Chloropyridazine-4-carbonitrile (14)

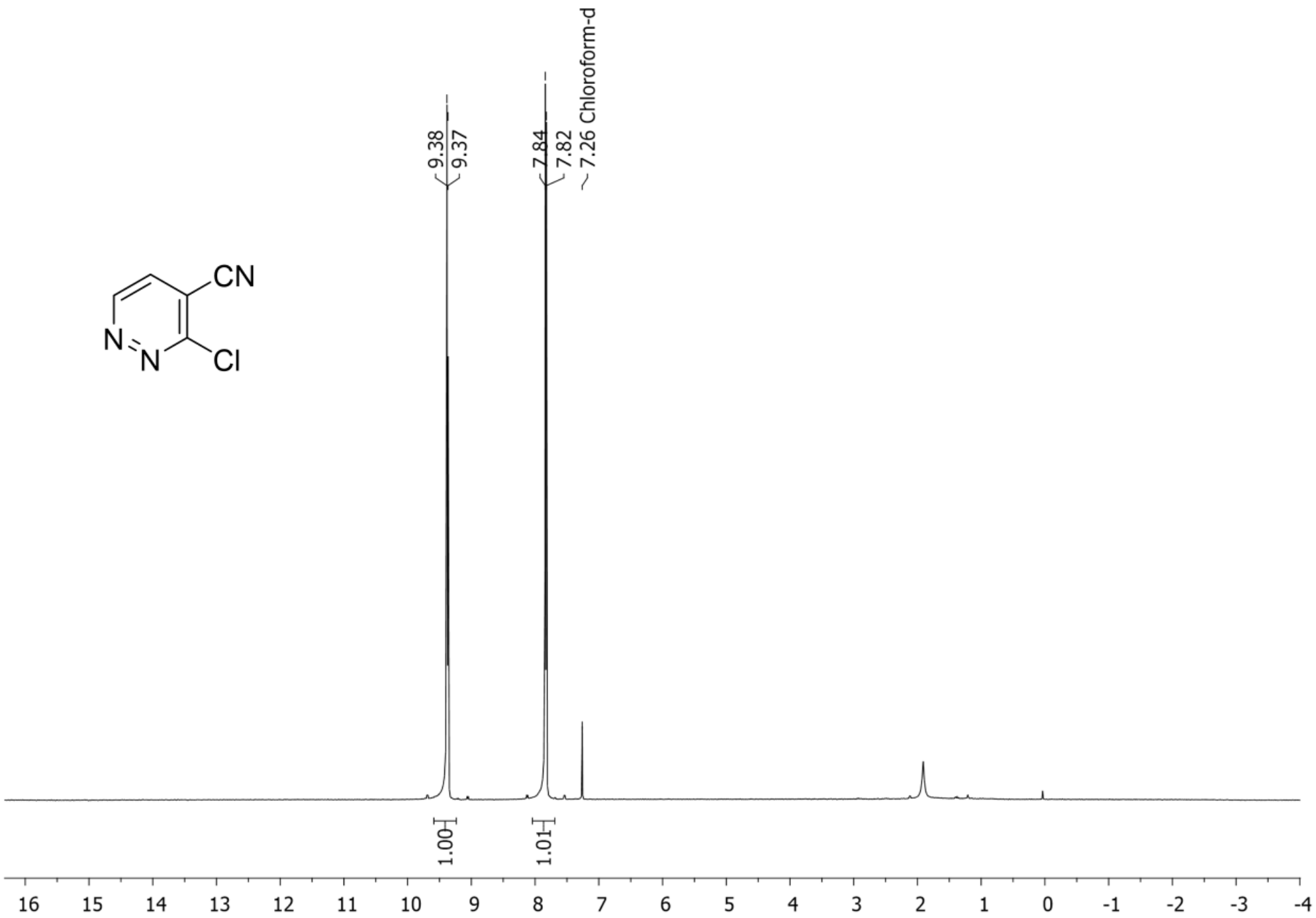

^{1}H NMR (75 MHz, $CDCl_3$) spectrum of Chloropyridazine-4-carbonitrile (14)

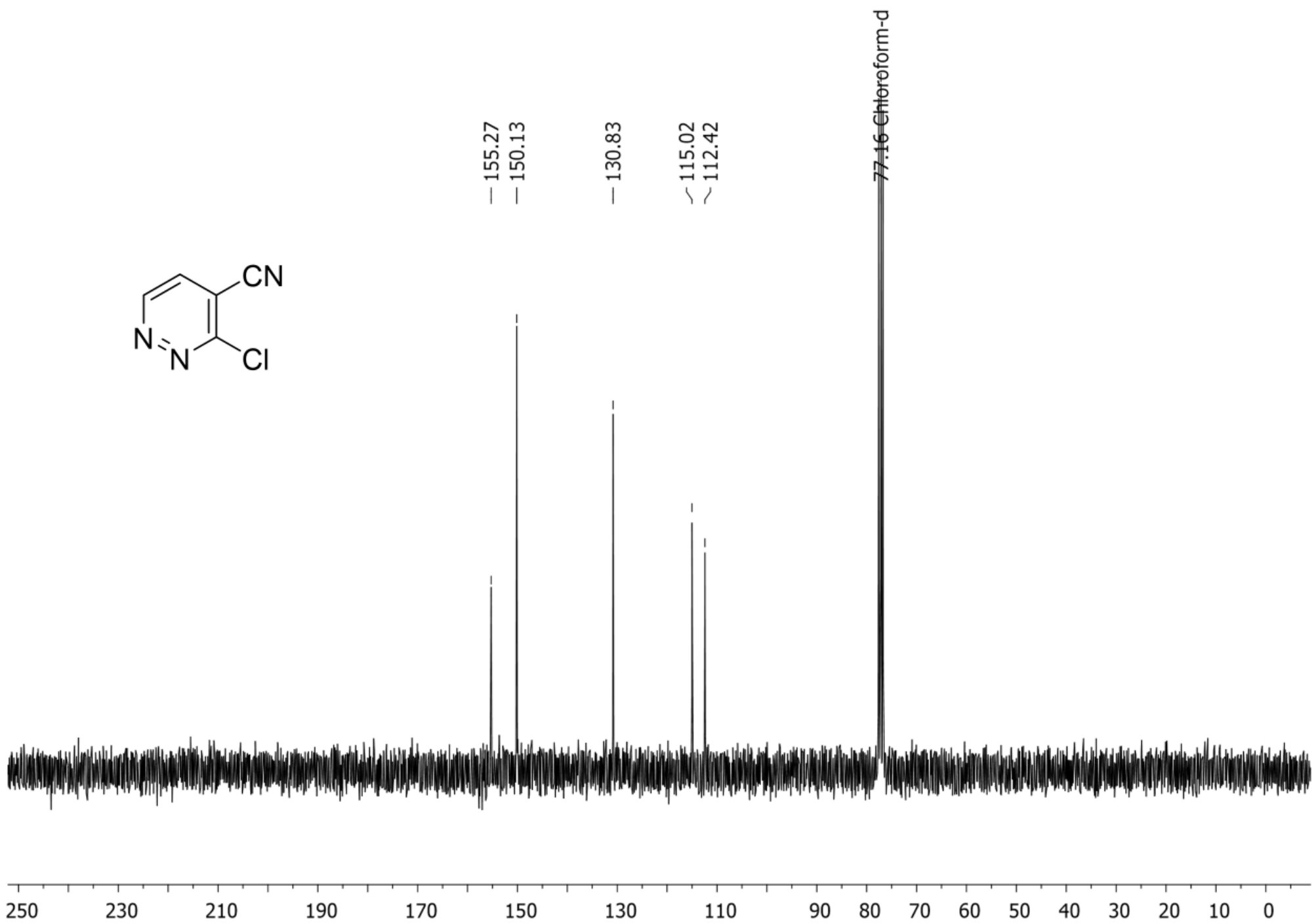

^{1}H NMR (300 MHz, $CDCl_3$) spectrum of 3-Chloro-5,6-dimethylpyridazine-4-carbonitrile (15)

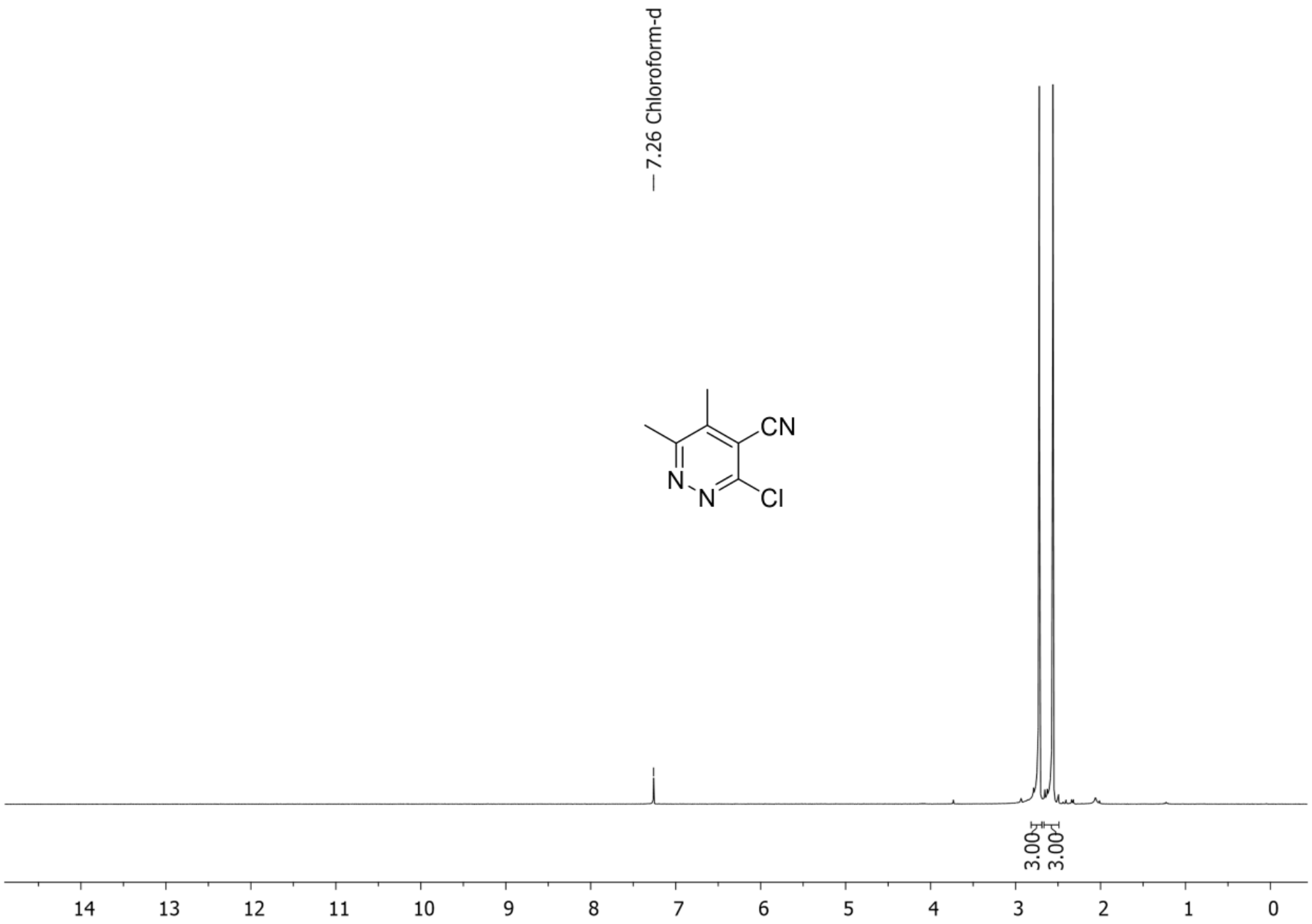

^{13}C NMR (75 MHz, $CDCl_3$) spectrum of 3-Chloro-5,6-dimethylpyridazine-4-carbonitrile (15)

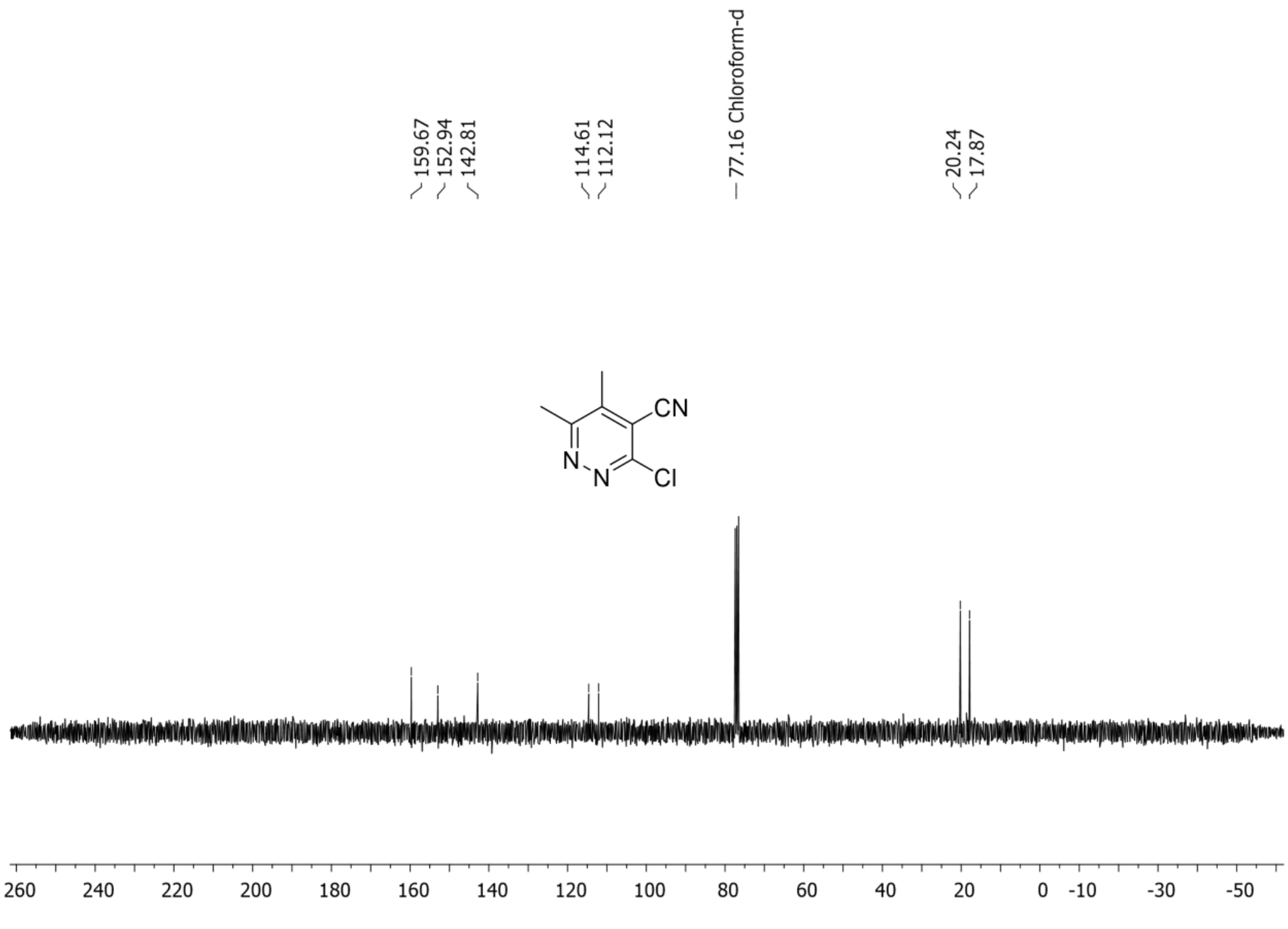

^{1}H NMR (300 MHz, $CDCl_3$) spectrum of Diethyl 2-(4-cyanopyridazin-3-yl) malonate (16)

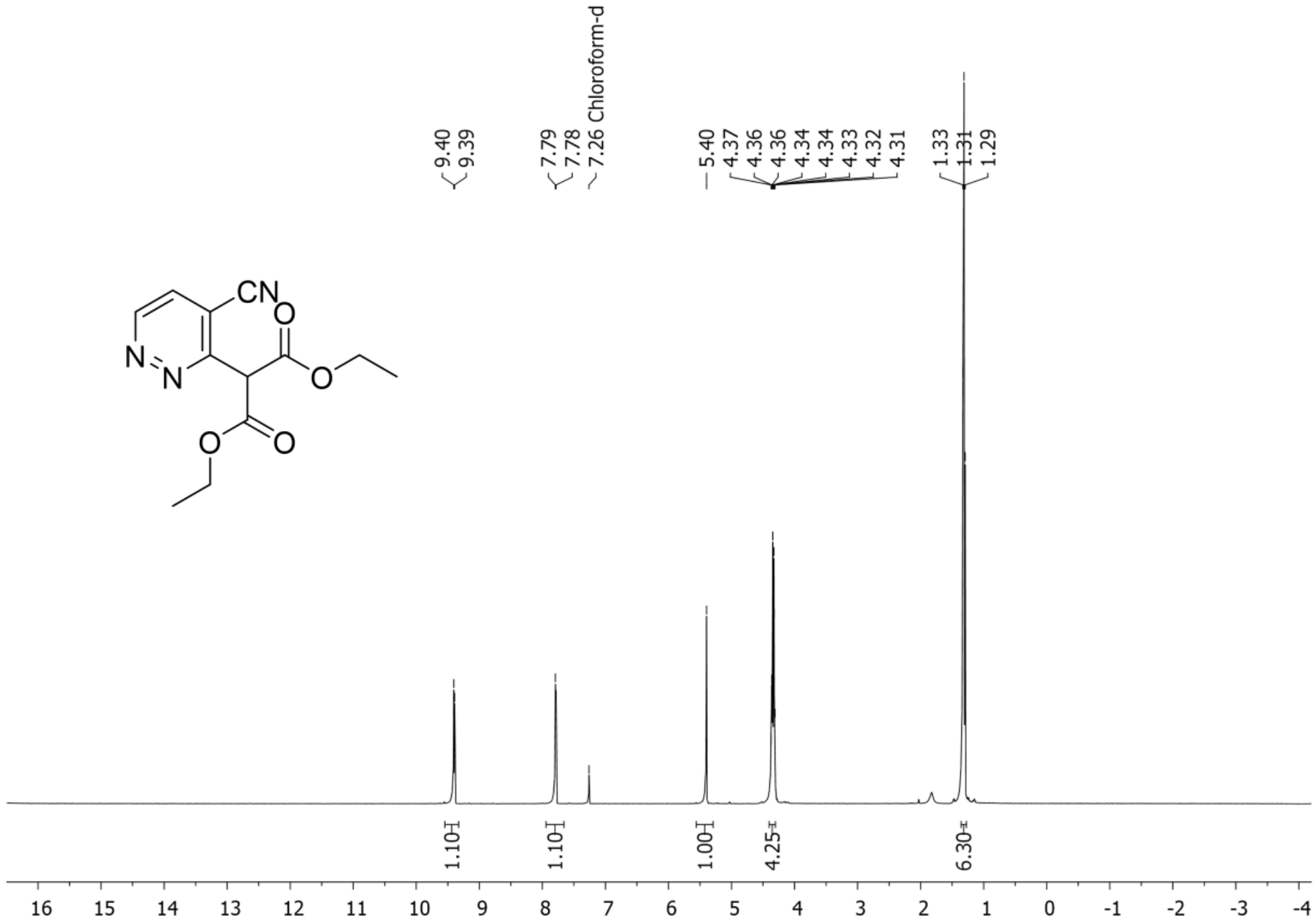

^{1}H NMR (75 MHz, $CDCl_3$) spectrum of Diethyl 2-(4-cyanopyridazin-3-yl) malonate (16)

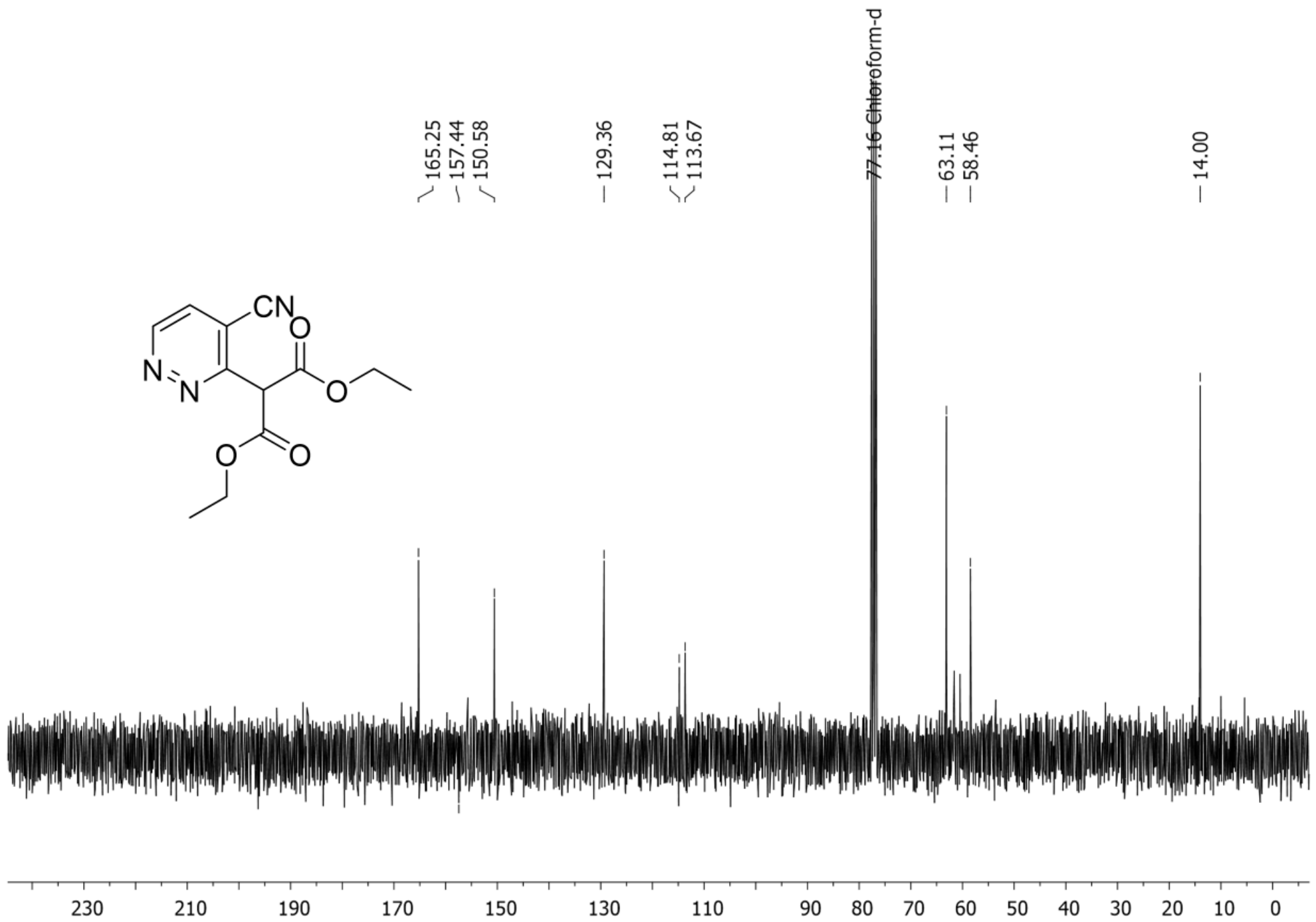

^{1}H NMR (300 MHz, $CDCl_3$) spectrum of Diethyl 2-(4-cyano-5,6-methylpyridazin-3-yl) malonate (17)

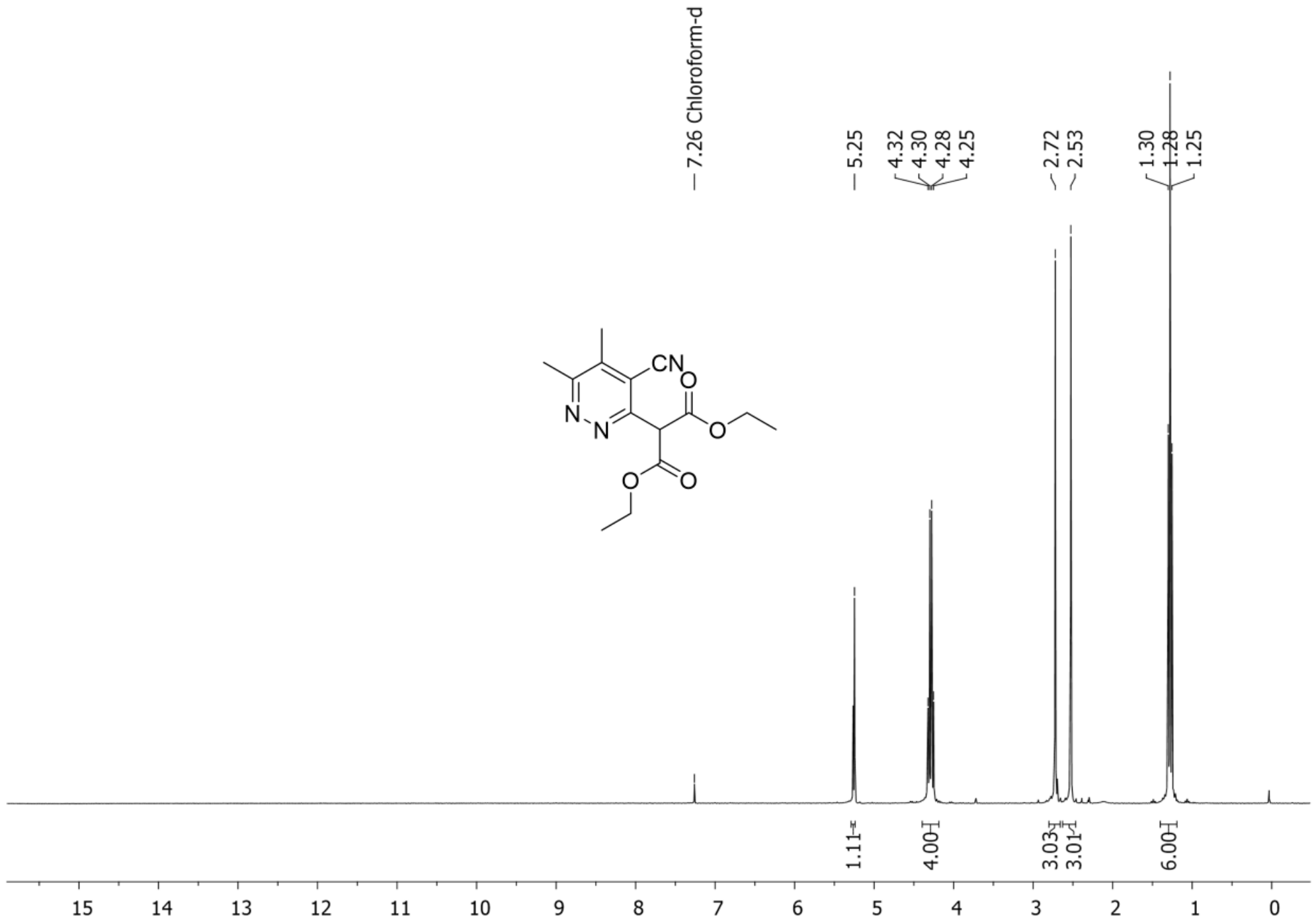

^{1}H NMR (300 MHz, $CDCl_3$) spectrum of Diethyl 2-(4-cyano-5,6-methylpyridazin-3-yl) malonate (17)

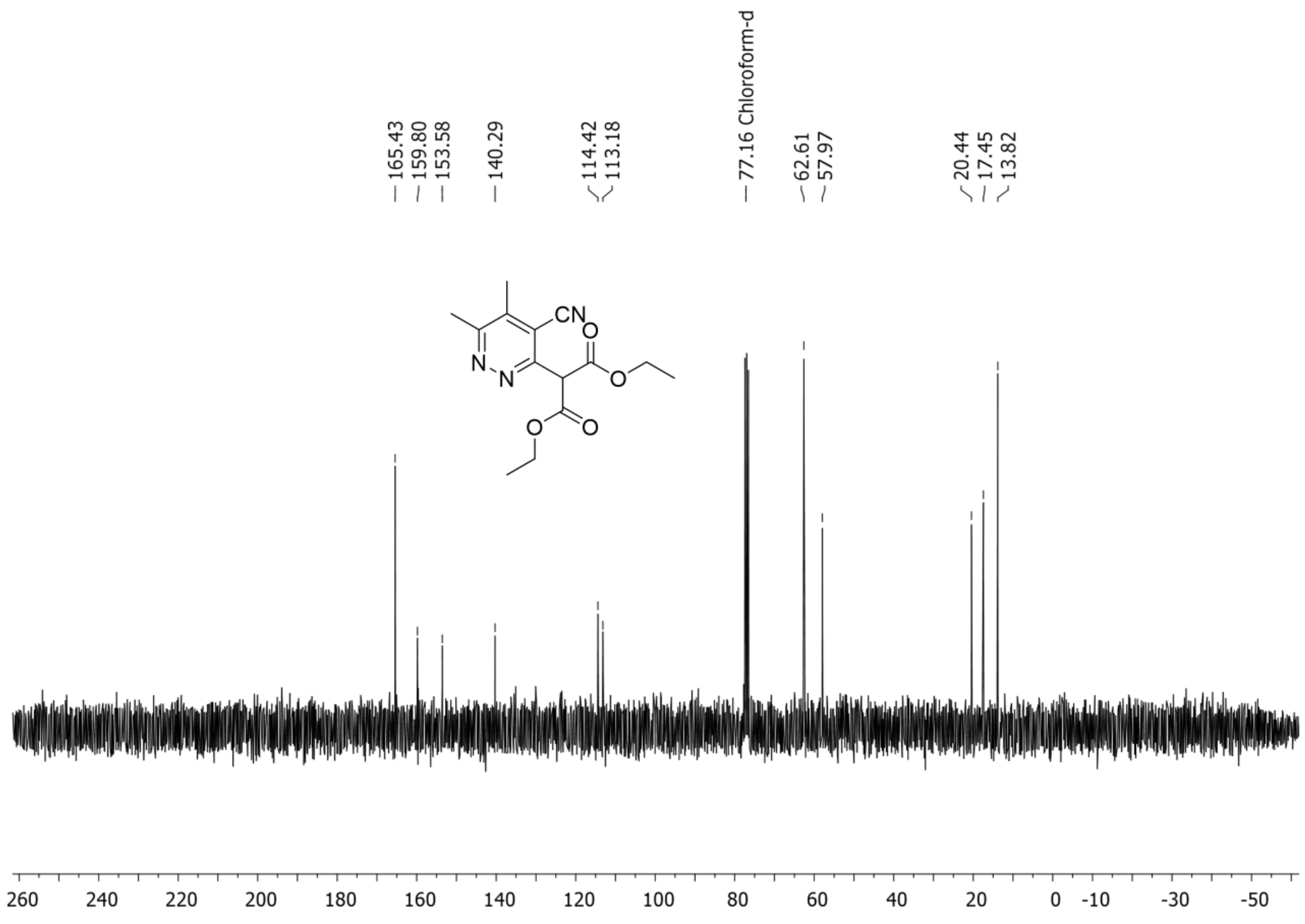

^{1}H NMR (300 MHz, $CDCl_3$) spectrum of Diethyl 2-(3-cyanopyridin-2-yl)malonate (18)

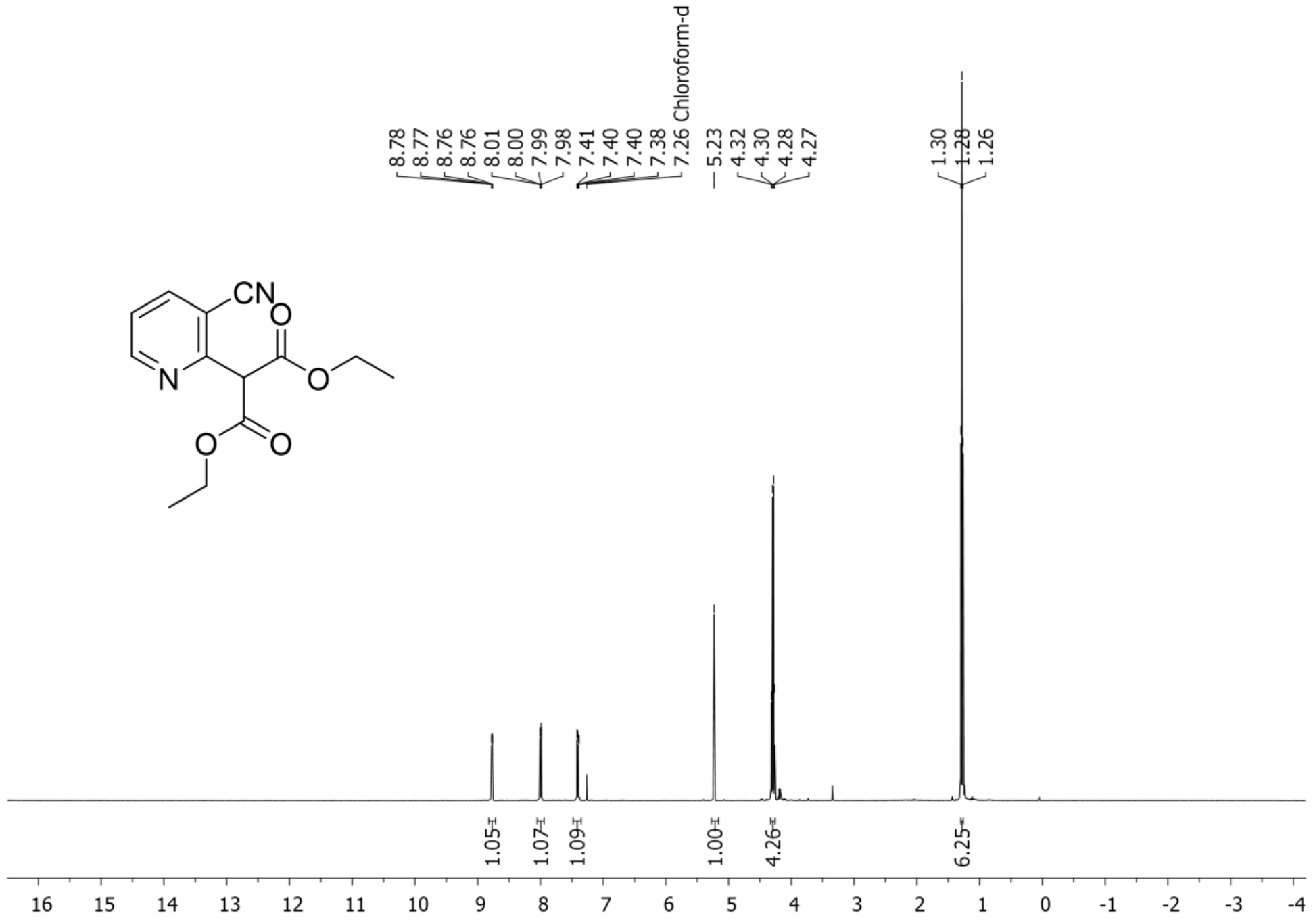

^{1}H NMR (75 MHz, $CDCl_3$) spectrum of Diethyl 2-(3-cyanopyridin-2-yl)malonate (18)

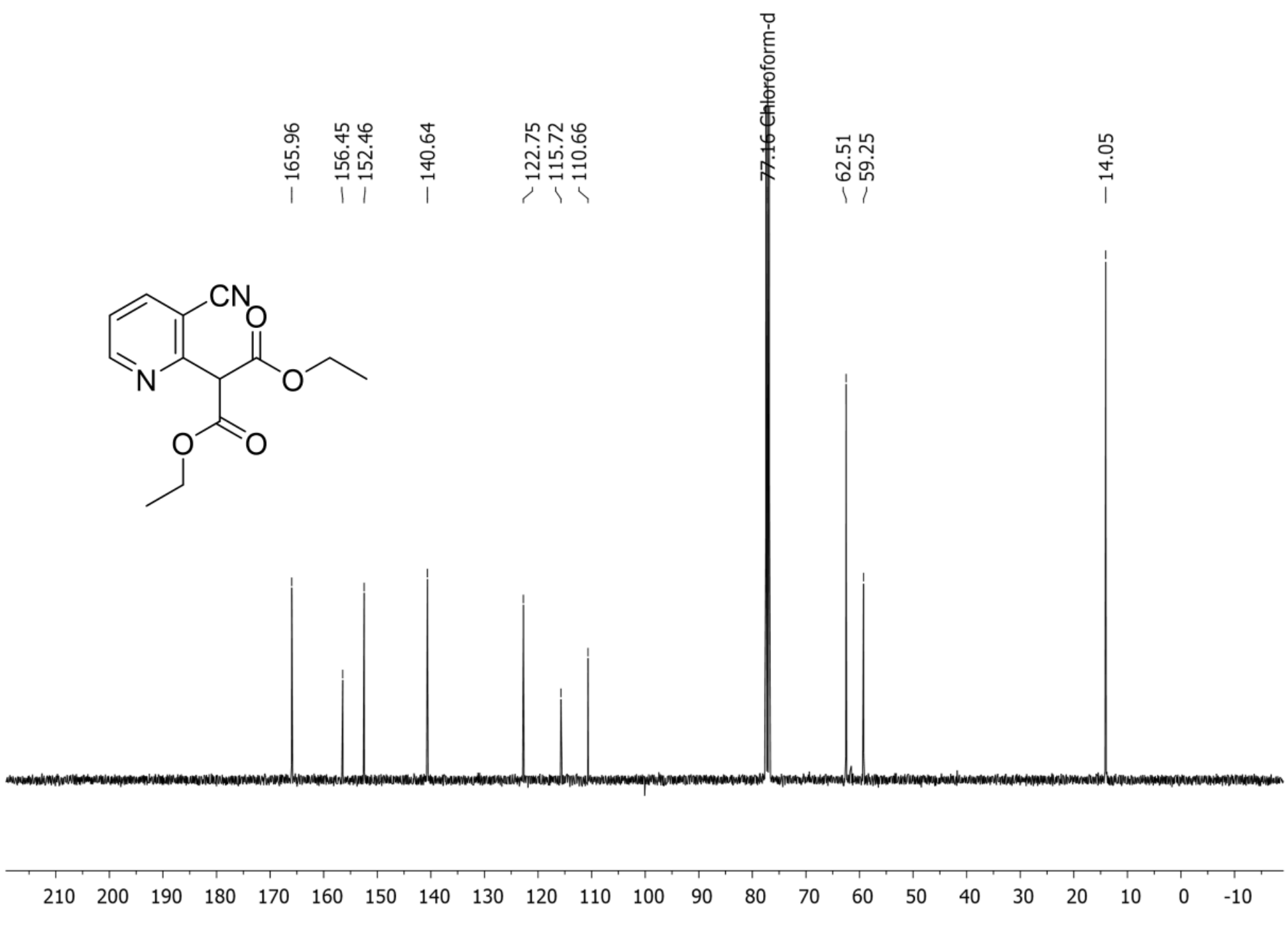

^{1}H NMR (300 MHz, CDCl₃) spectrum of Diethyl 2-(4-cyanopyridin-3-yl)malonate (20)

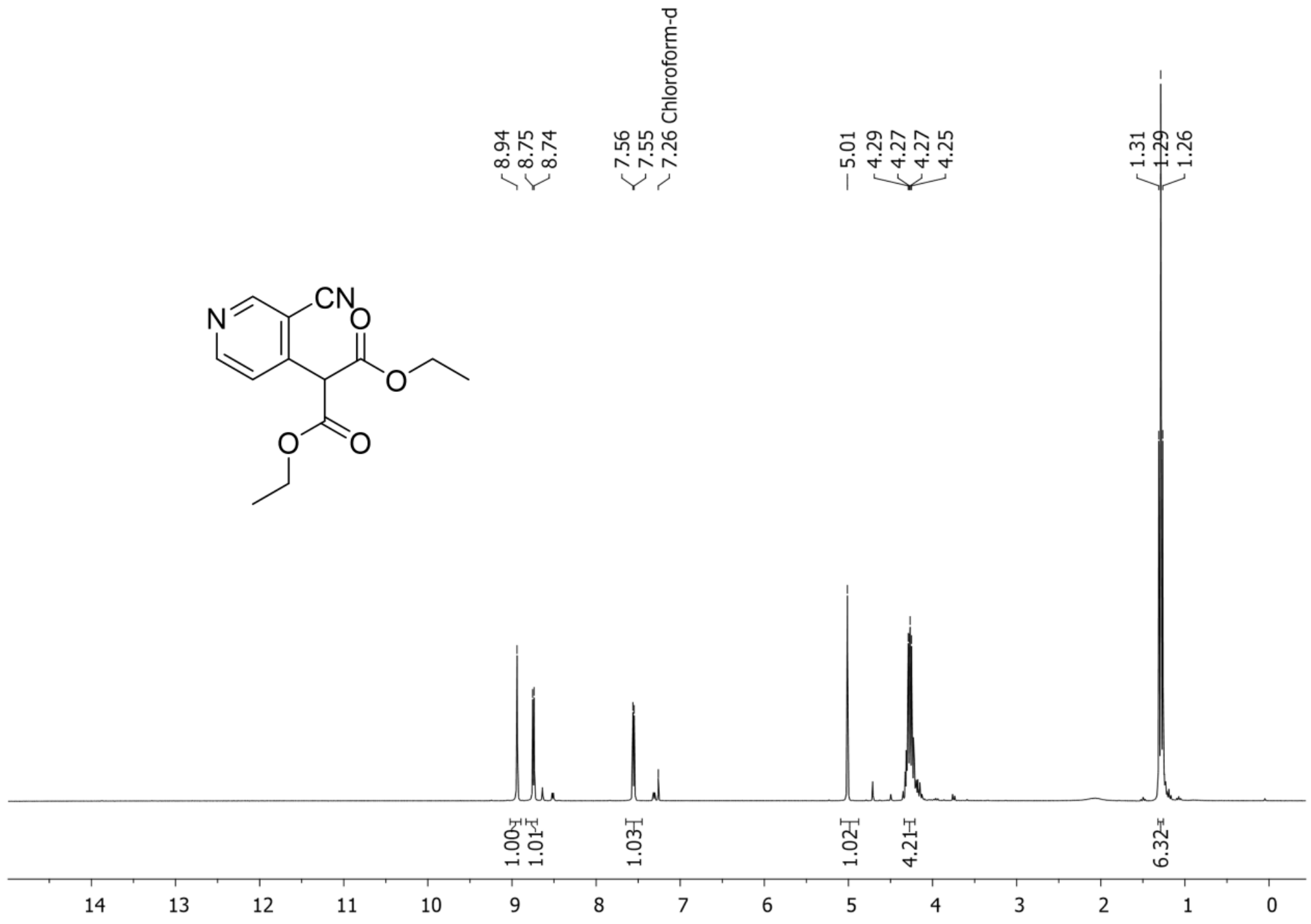

^{1}H NMR (75 MHz, CDCl₃) spectrum of Diethyl 2-(4-cyanopyridin-3-yl)malonate (20)

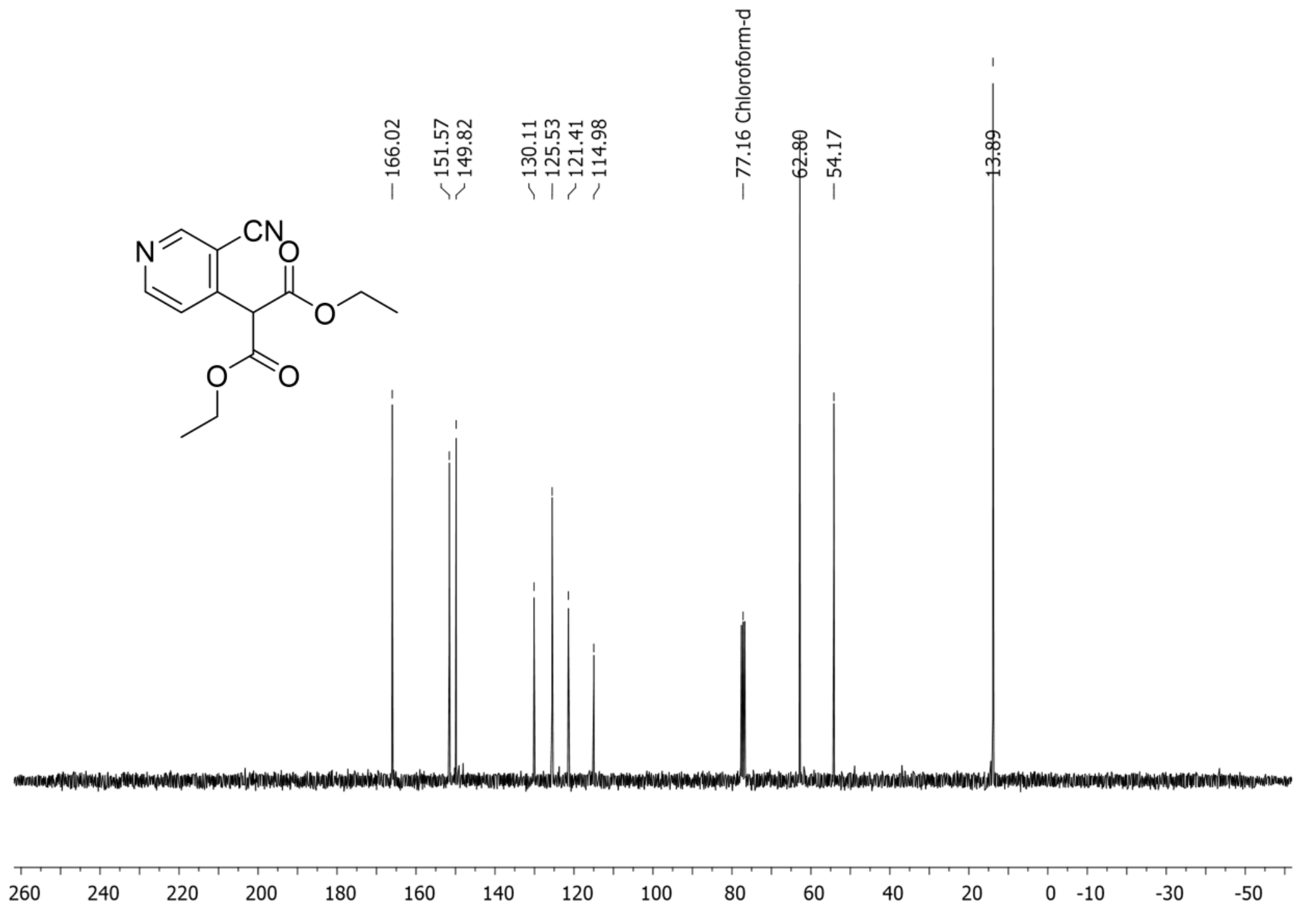

^{1}H NMR (300 MHz, $CDCl_3$) spectrum of Diethyl 2-(3-cyanopyridin-4-yl)malonate (21)

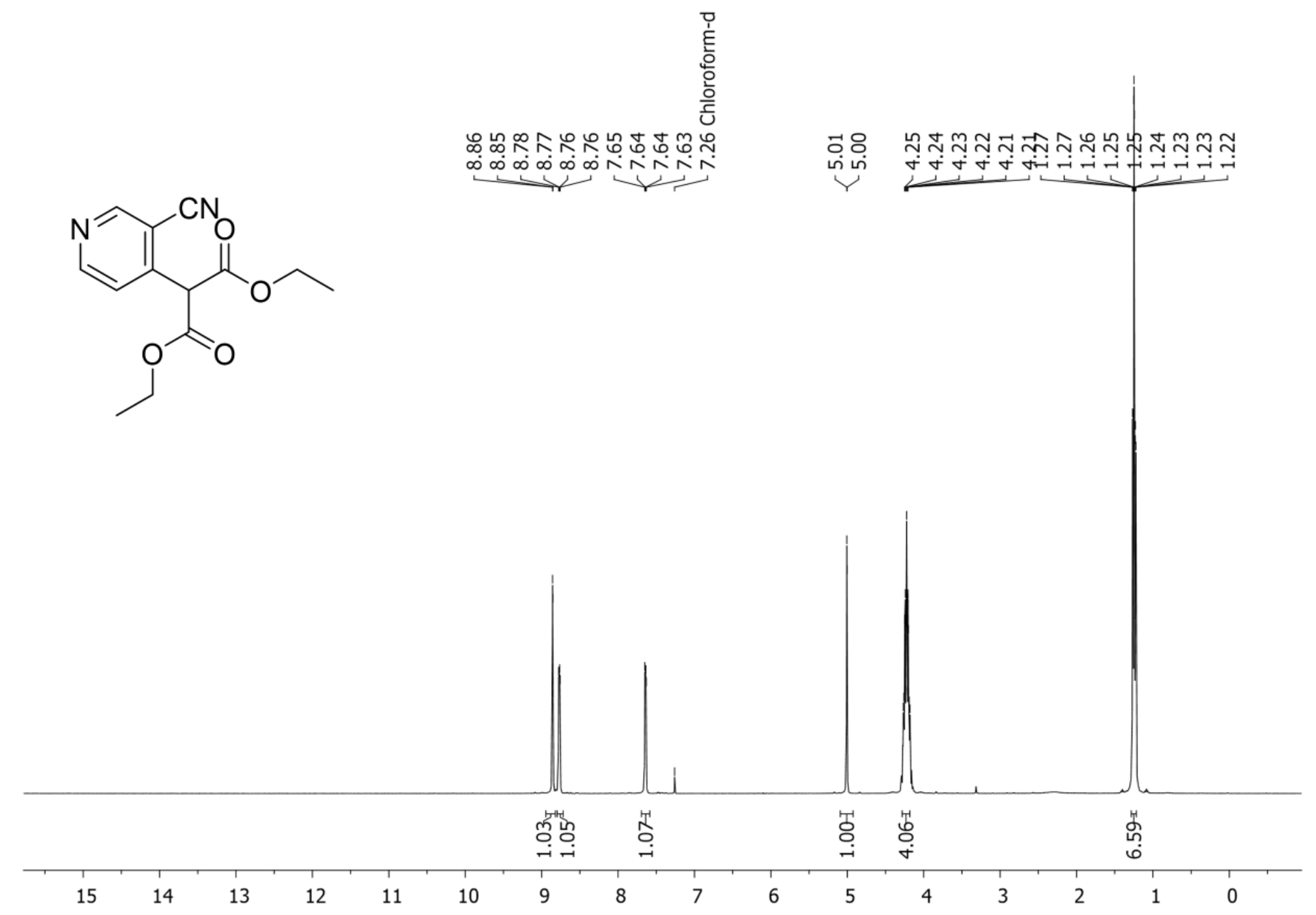

^{1}H NMR (75 MHz, $CDCl_3$) spectrum of Diethyl 2-(3-cyanopyridin-4-yl)malonate (21)

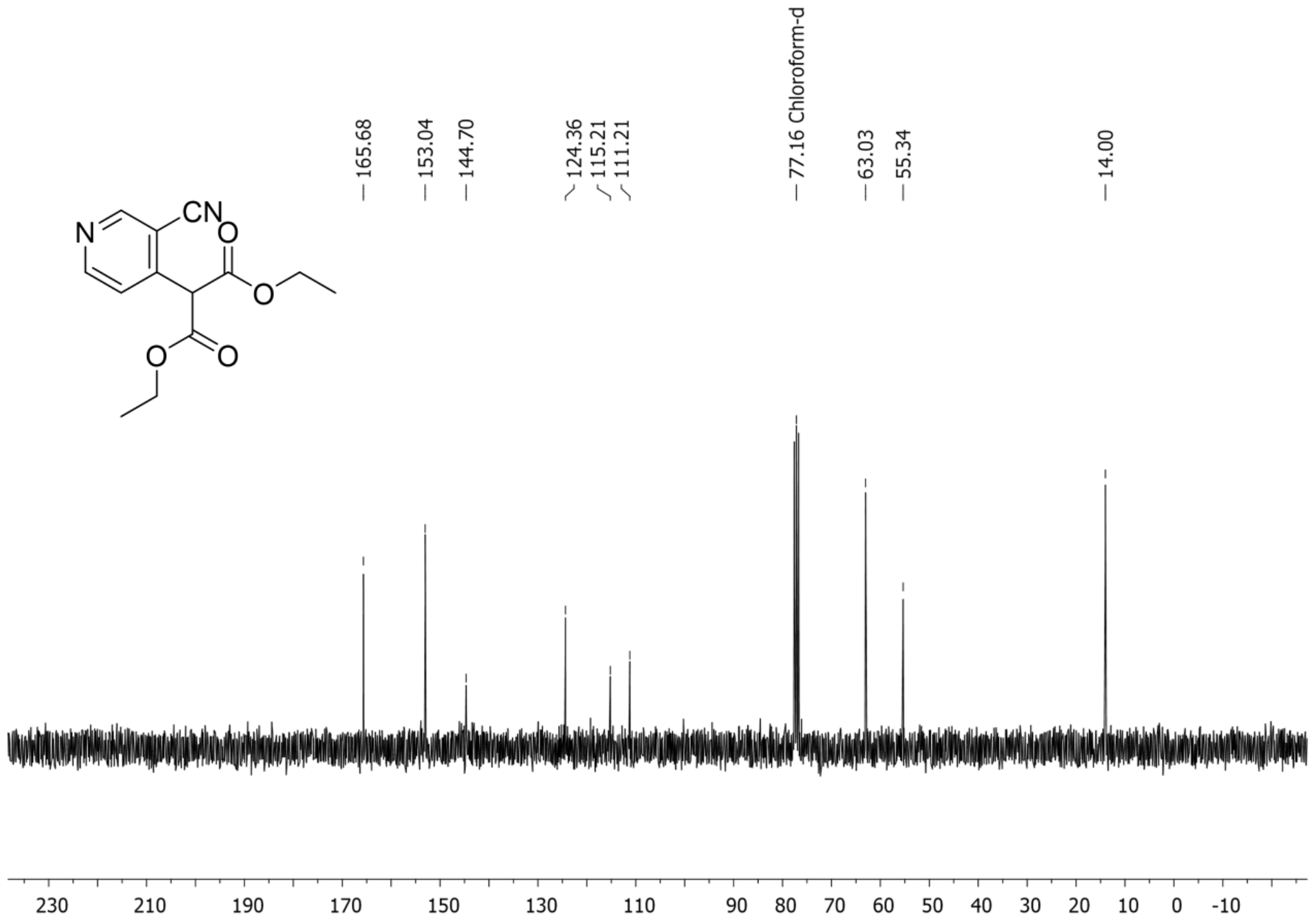

¹H NMR (300 MHz, CDCl₃) spectrum of Diethyl 2-(3-cyanopyrazin-2-yl)malonate (22)

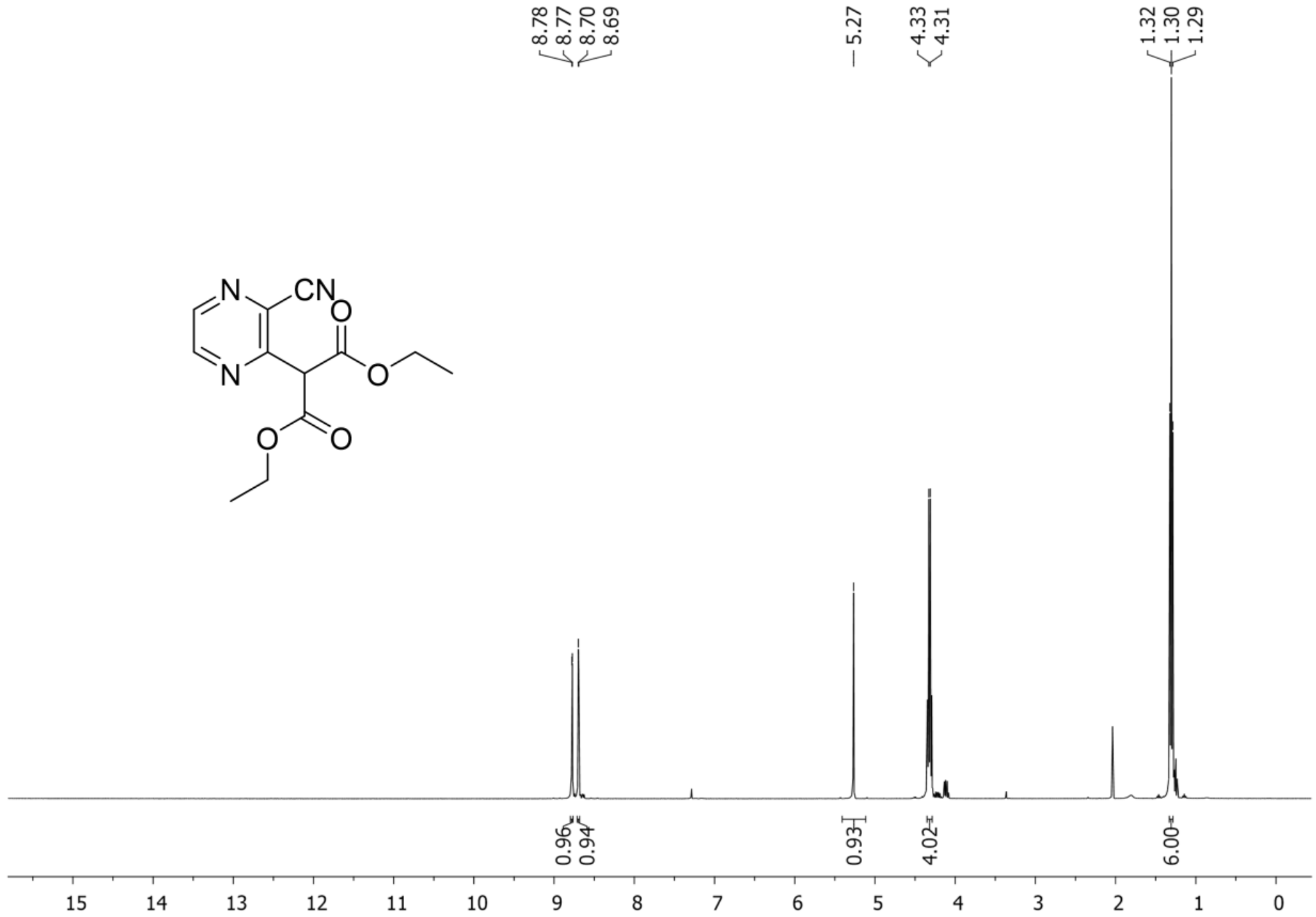

¹H NMR (75 MHz, CDCl₃) spectrum of Diethyl 2-(3-cyanopyrazin-2-yl)malonate (22)

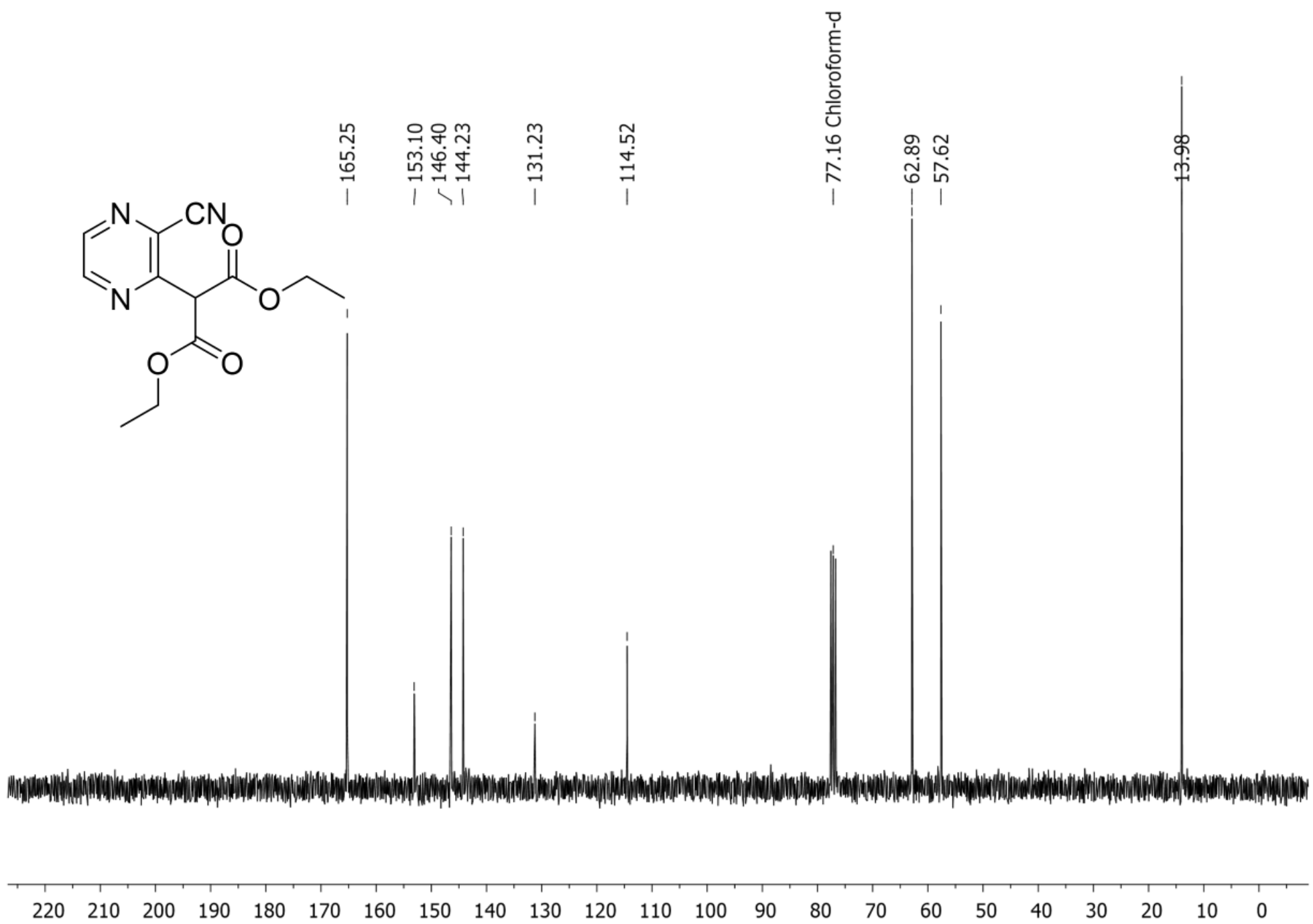

^1H NMR (300 MHz, Acetone) spectrum of Diethyl-2-(amino(3-chloropyridin-2-yl)methylene)malonate (23)

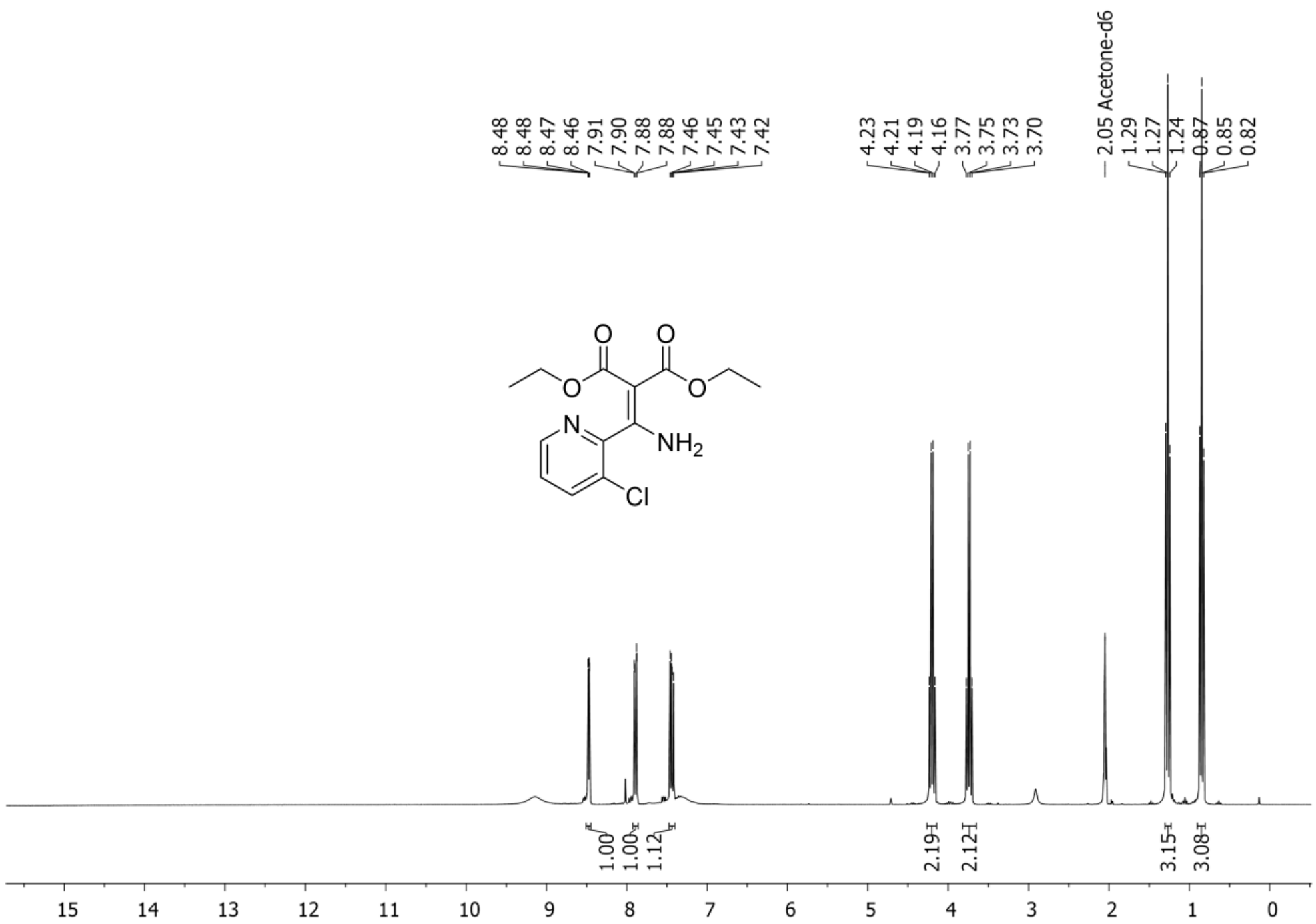

^{13}C NMR (75 MHz, Acetone) spectrum of Diethyl-2-(amino(3-chloropyridin-2-yl)methylene)malonate (23)

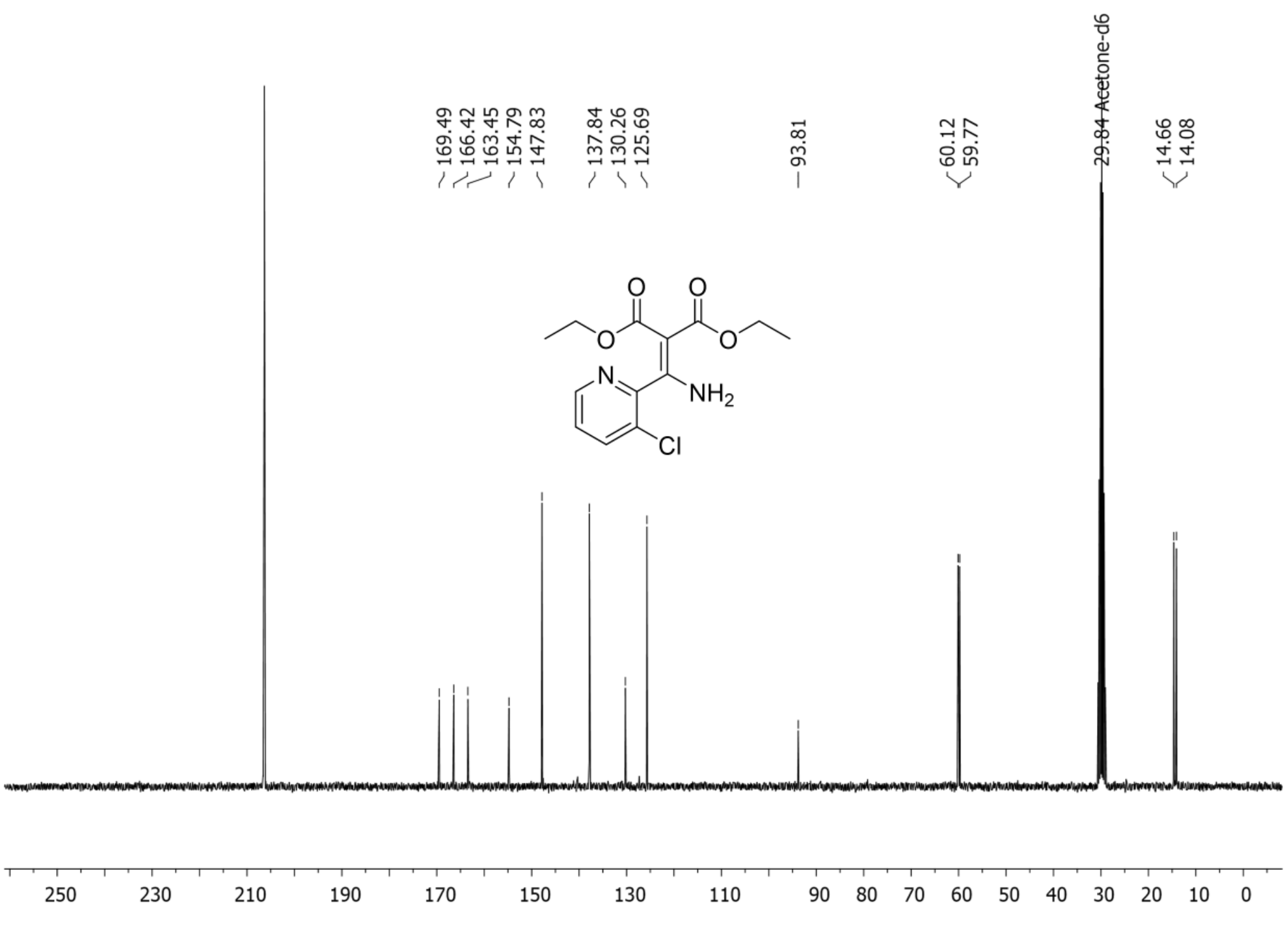

^{1}H NMR (300 MHz, Acetone) spectrum of Diethyl 2-(amino(3-chloropyridin-4-yl)methylene)malonate (24)

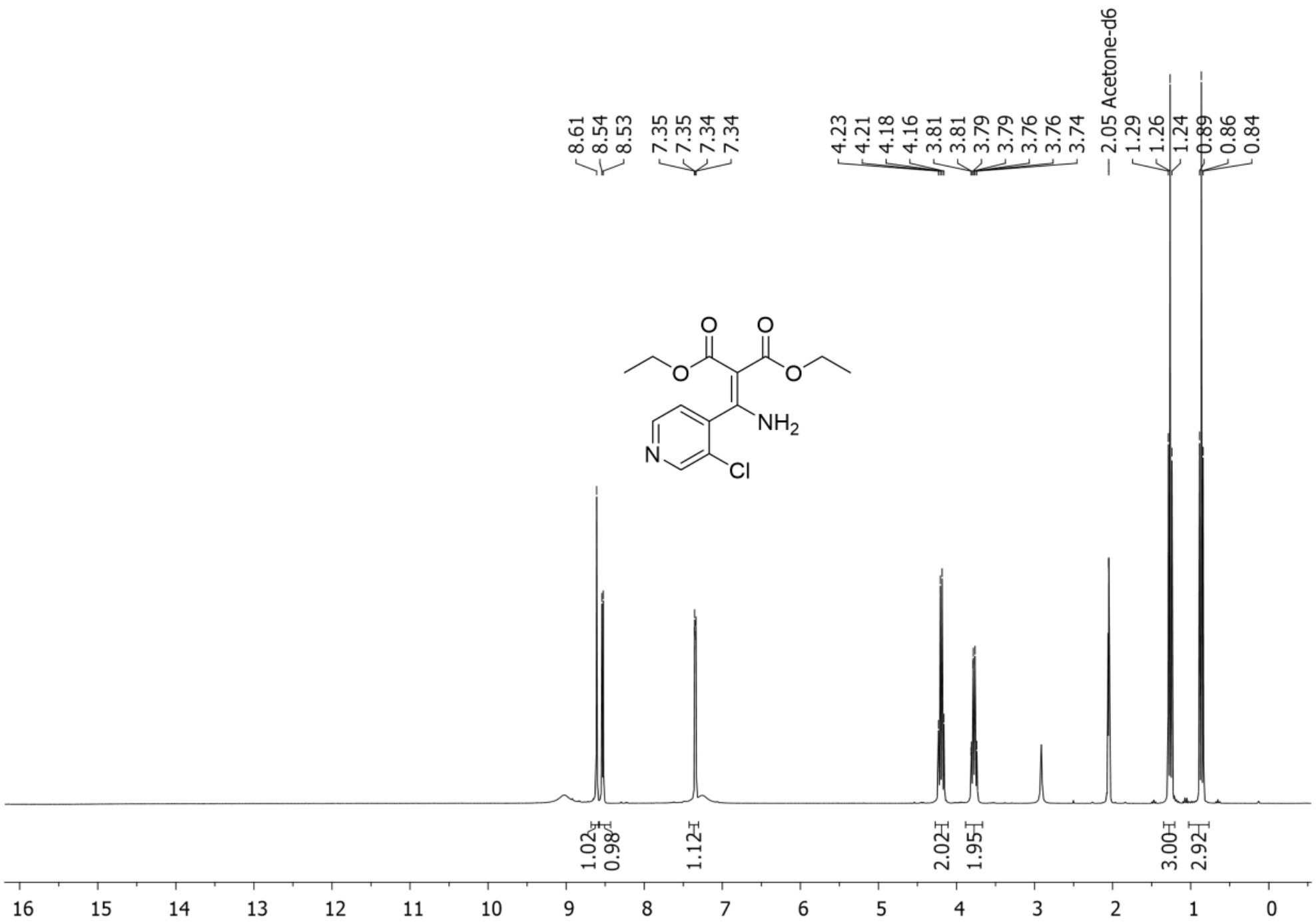

^{13}C NMR (75 MHz, Acetone) spectrum of Diethyl 2-(amino(3-chloropyridin-4-yl)methylene)malonate (24)

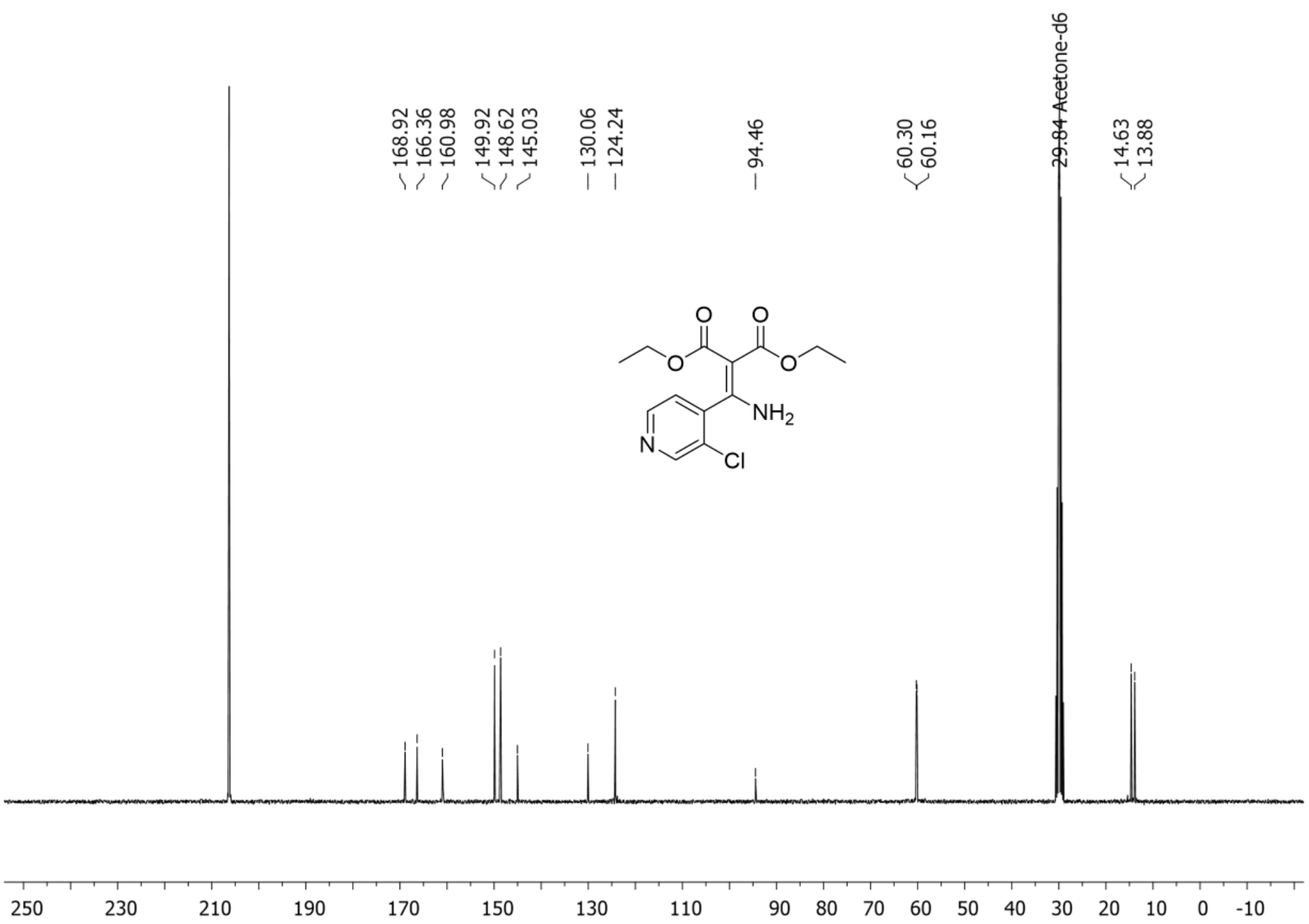

^{1}H NMR (300 MHz, $CDCl_3$) spectrum of Ethyl 2-(4-cyanopyridazin-3-yl) acetate (25)

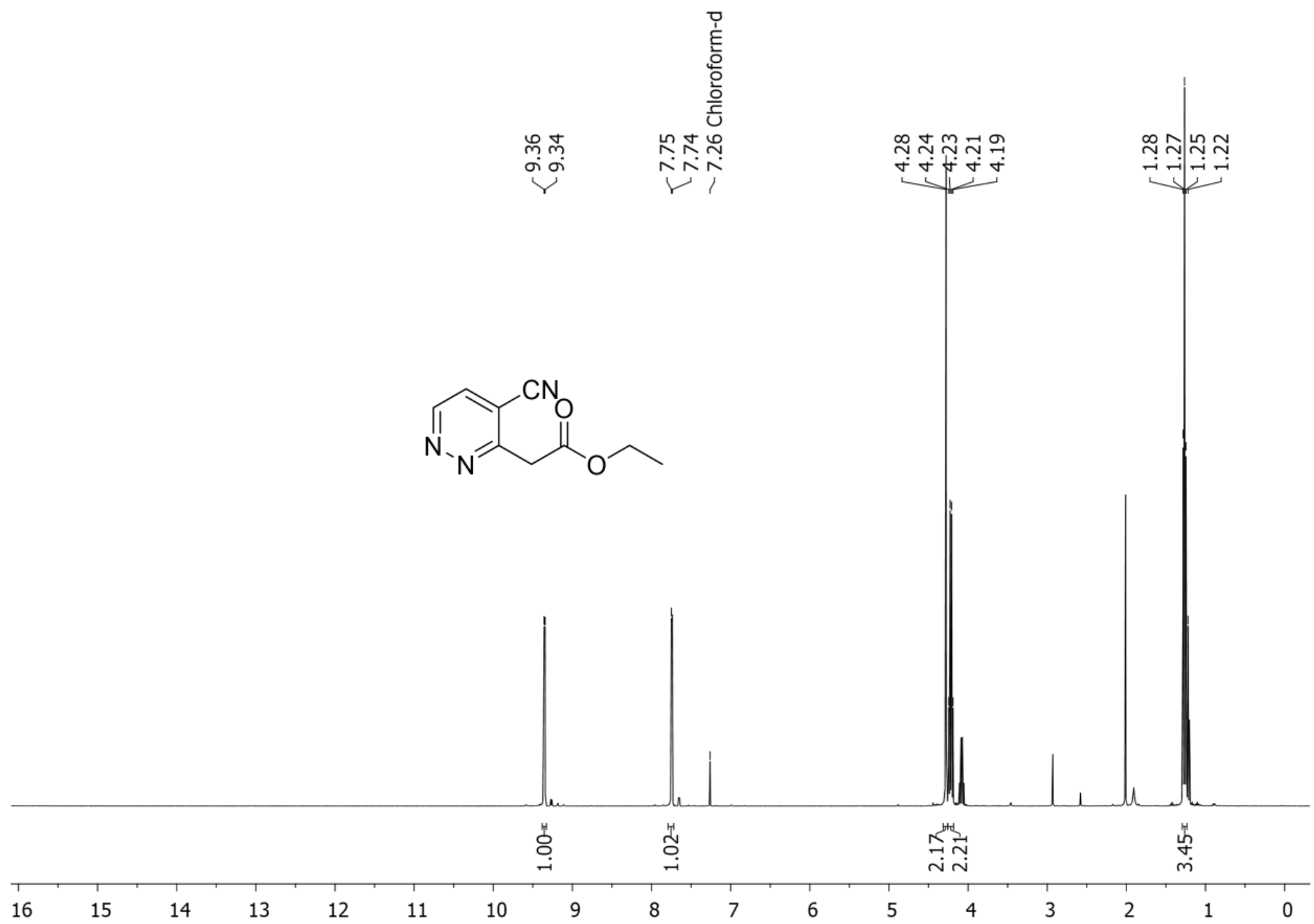

^{13}C NMR (75 MHz, $CDCl_3$) spectrum of Ethyl 2-(4-cyanopyridazin-3-yl) acetate (25)

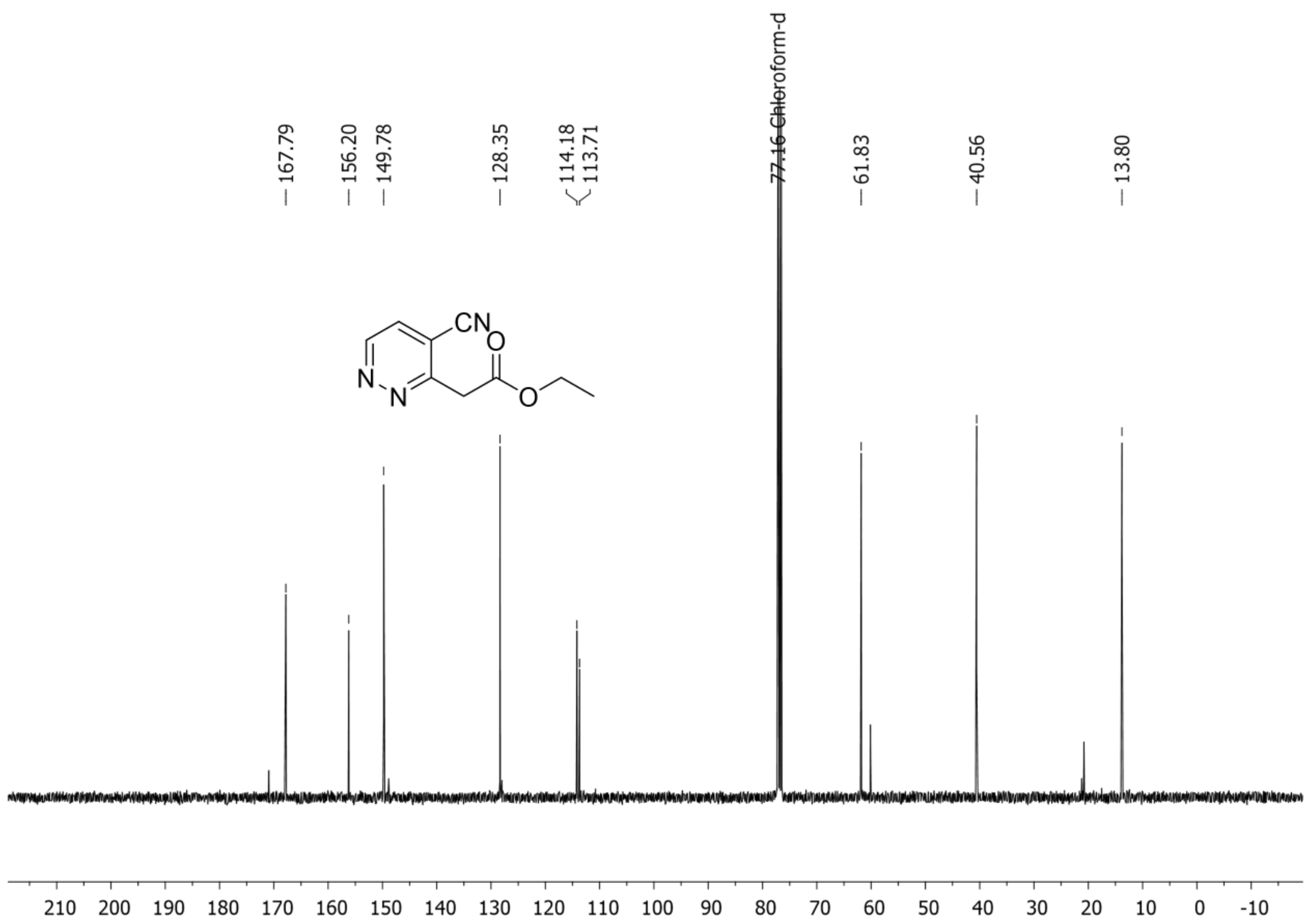

^{1}H NMR (300 MHz, $CDCl_3$) spectrum of Ethyl 2-(4-cyano-5,6-dimethylpyridazin-3-yl)acetate (26)

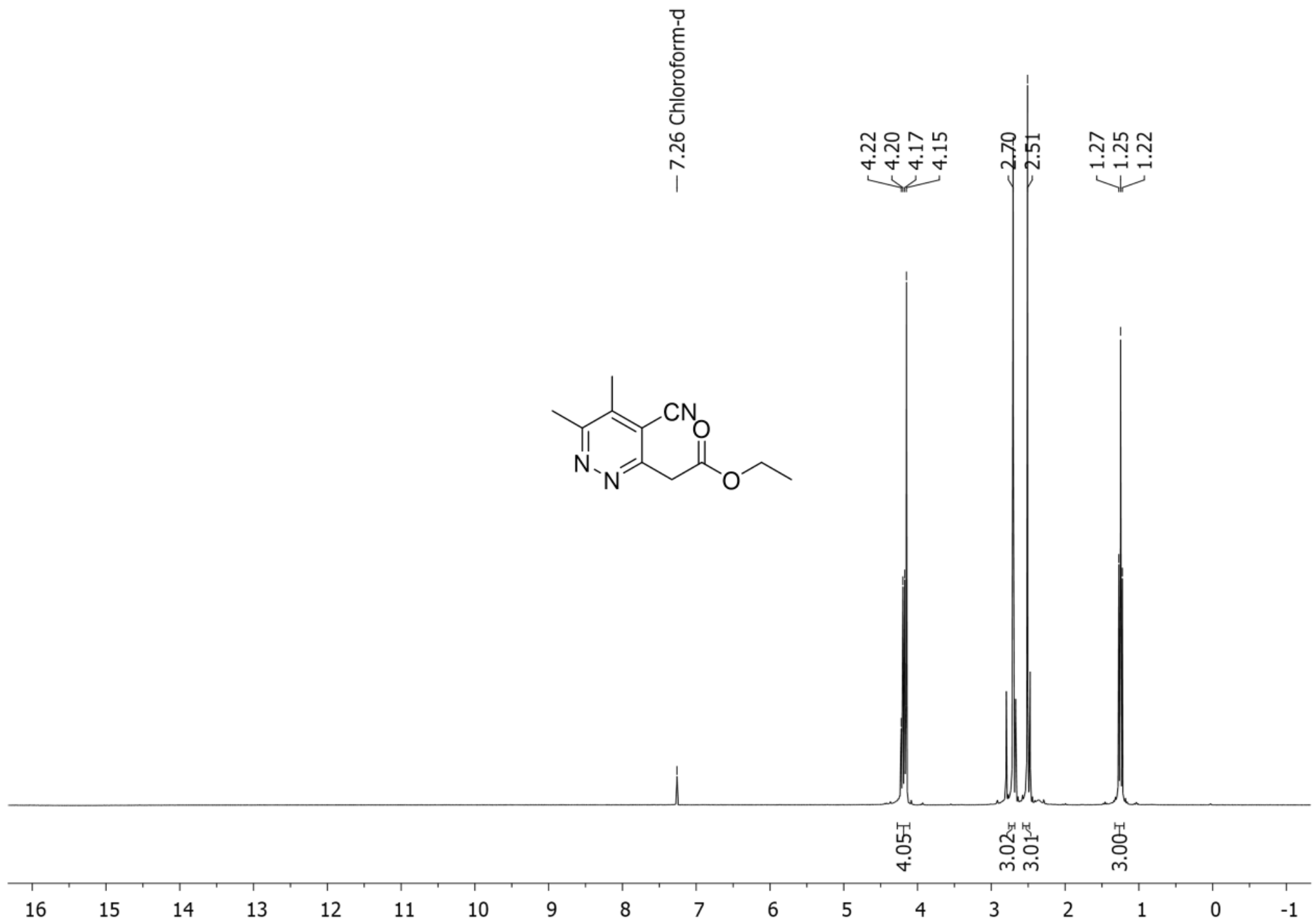

^{1}H NMR (75 MHz, $CDCl_3$) spectrum of Ethyl 2-(4-cyano-5,6-dimethylpyridazin-3-yl)acetate (26)

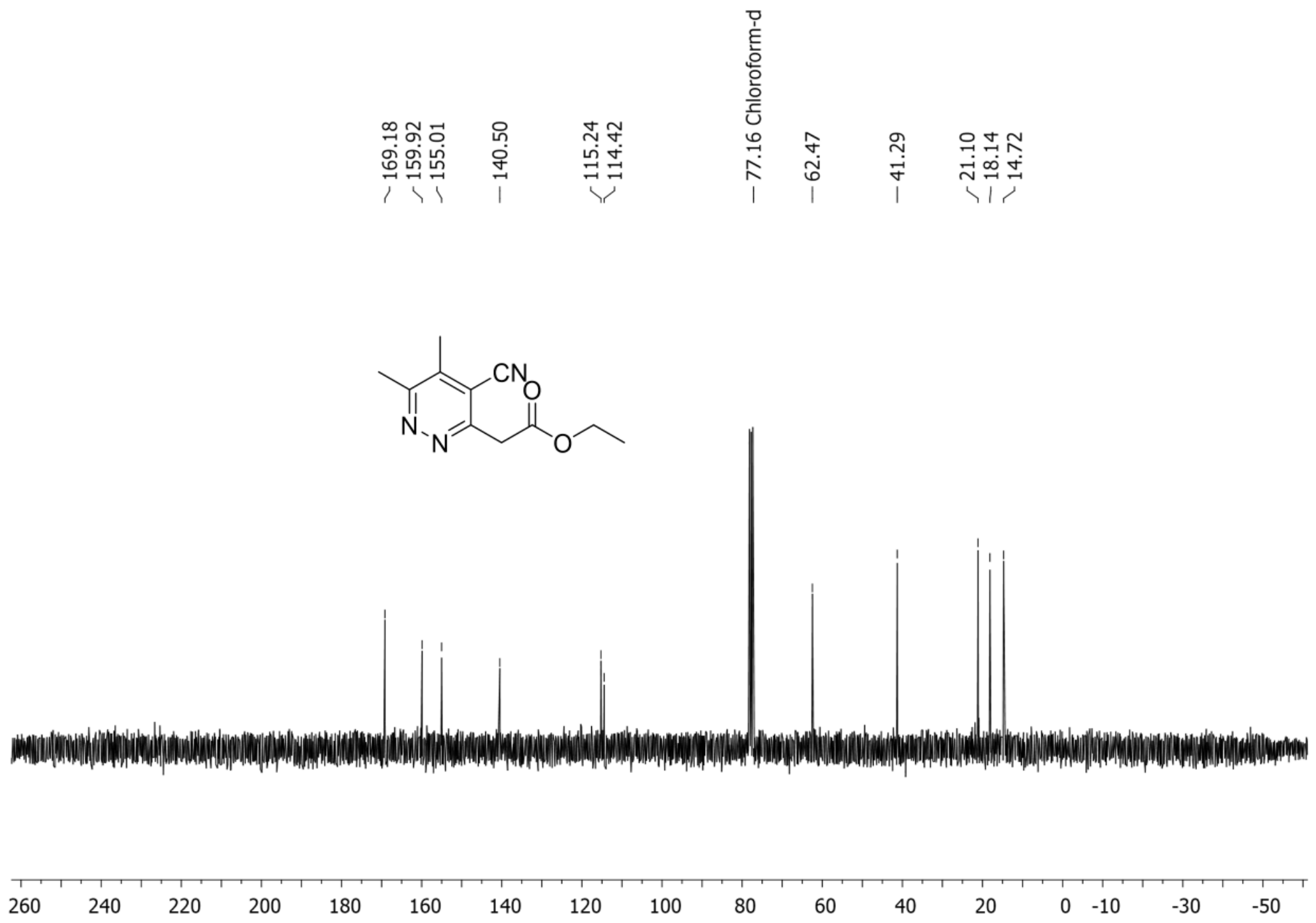

^{1}H NMR (300 MHz, $CDCl_3$) spectrum of Ethyl 2-(3-cyanopyridin-2-yl)acetate (27)

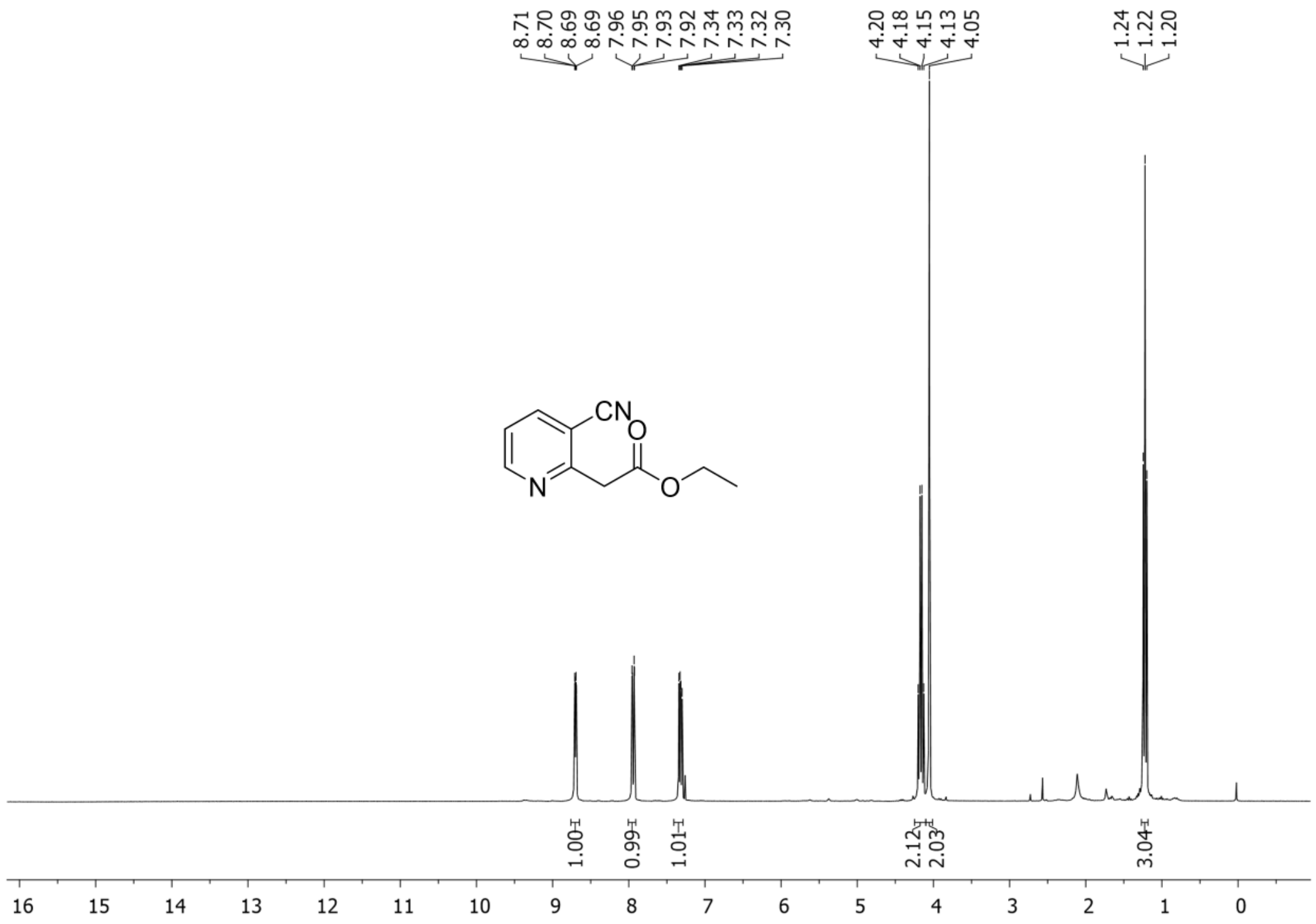

^{1}H NMR (300 MHz, $CDCl_3$) spectrum of Ethyl 2-(3-cyanopyridin-2-yl)acetate (27)

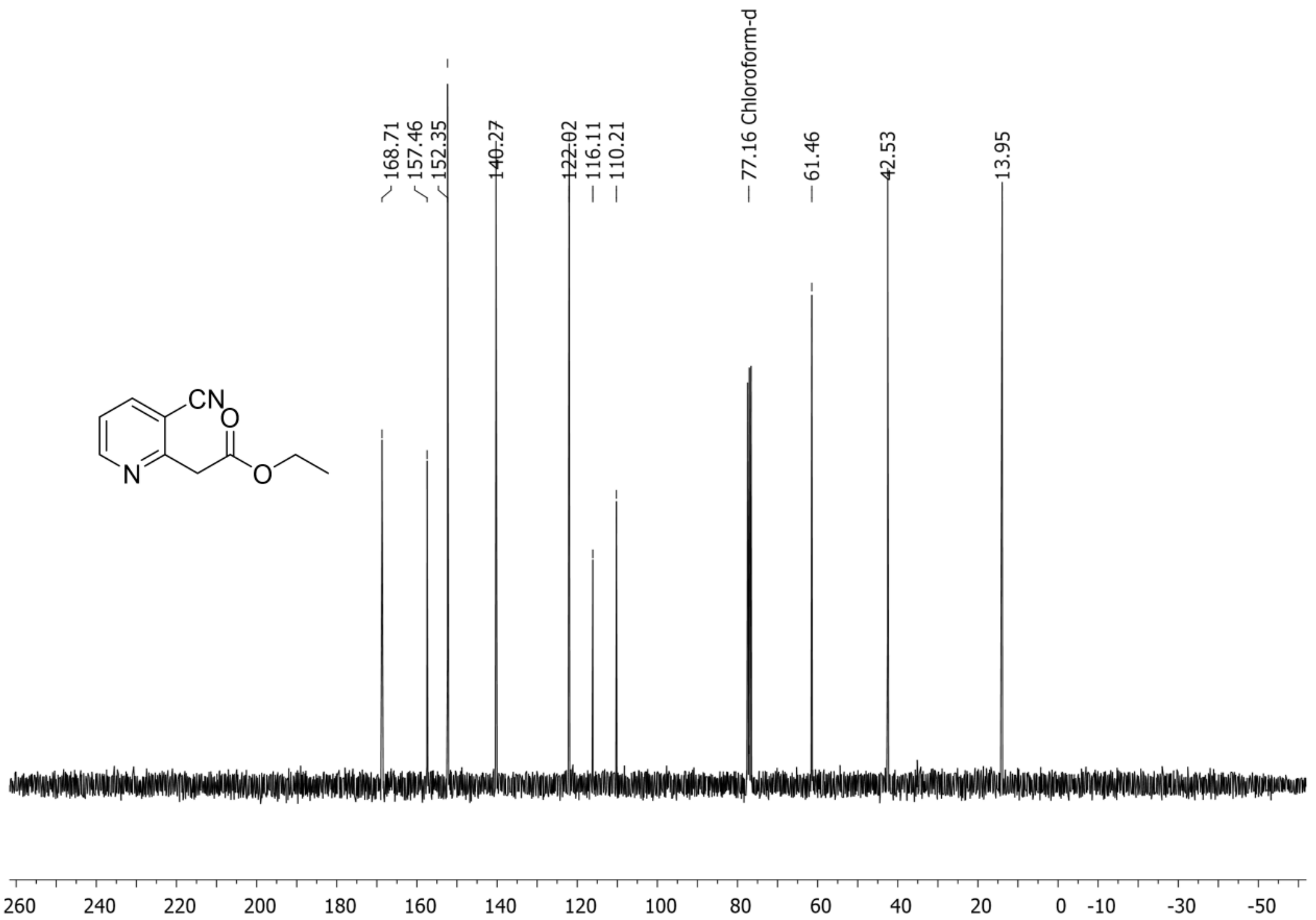

^{1}H NMR (300 MHz, $(CD_3)_2CO$) spectrum of Ethyl 2-(3-cyanopyridin-4-yl)acetate (28)

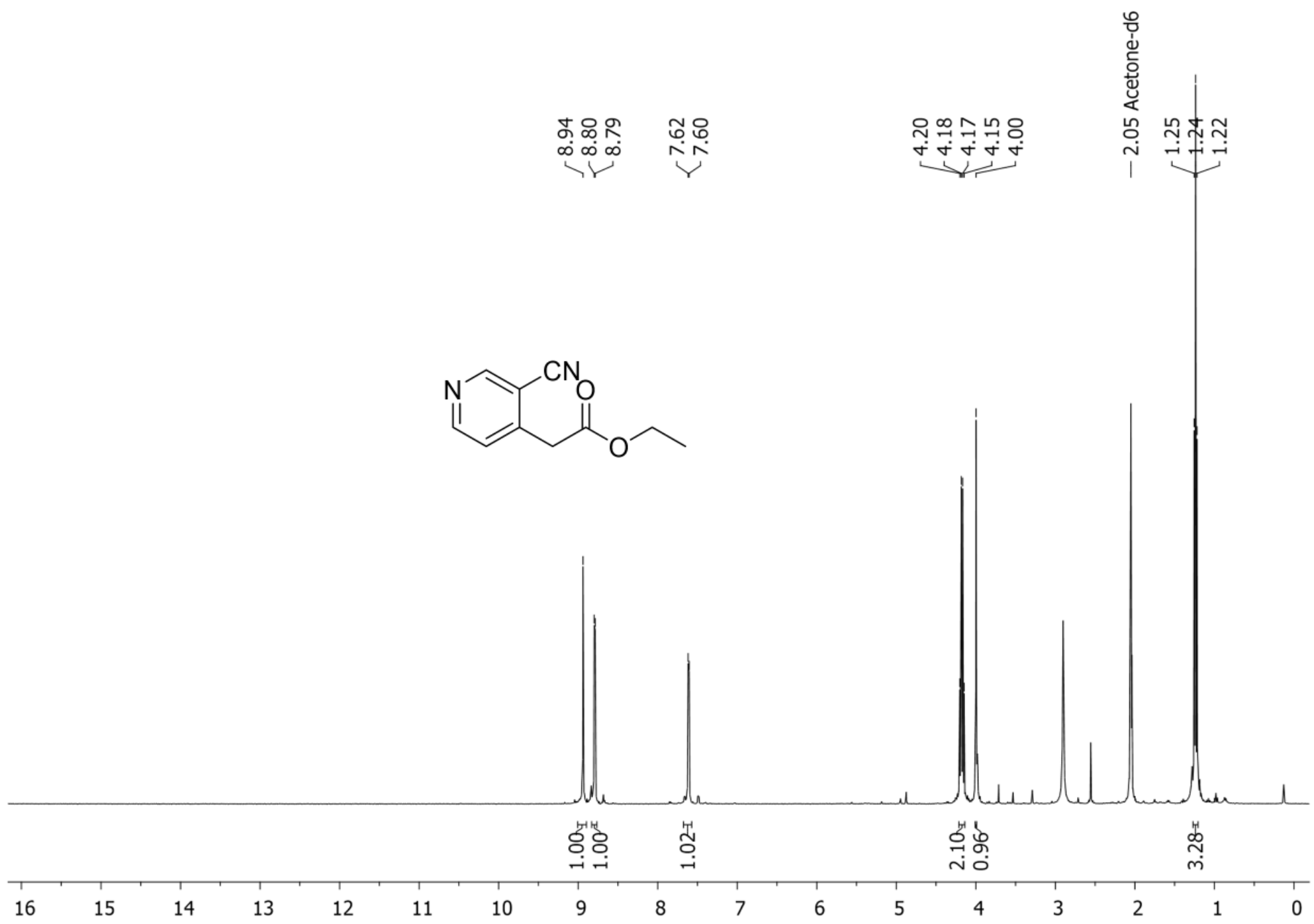

^{1}H NMR (75 MHz, $(CD_3)_2CO$) spectrum of Ethyl 2-(3-cyanopyridin-4-yl)acetate (28)

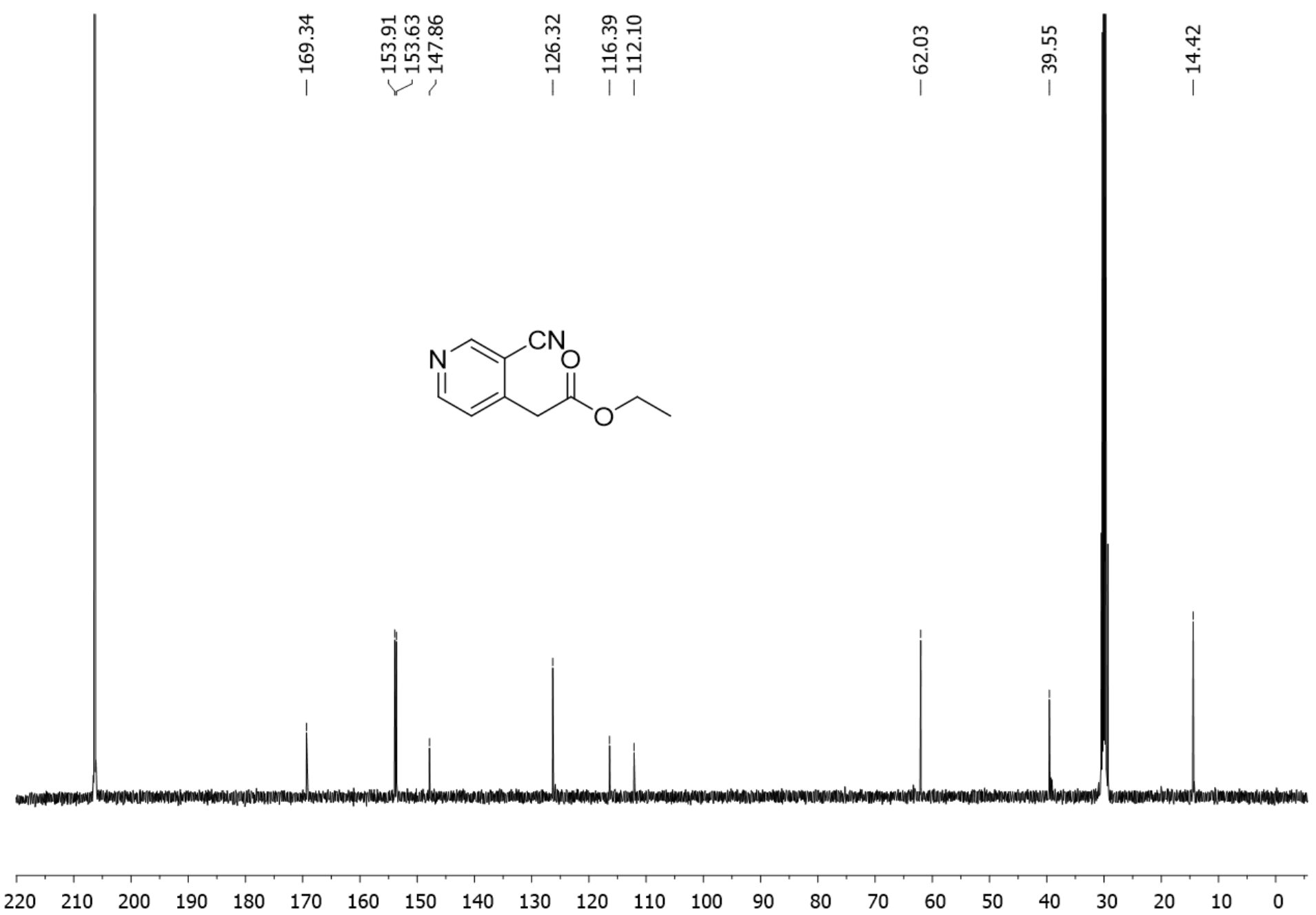

^{1}*H NMR (300 MHz, CDCl$_3$) spectrum of Ethyl 2-(4-cyanopyridin-3-yl)acetate (29)*

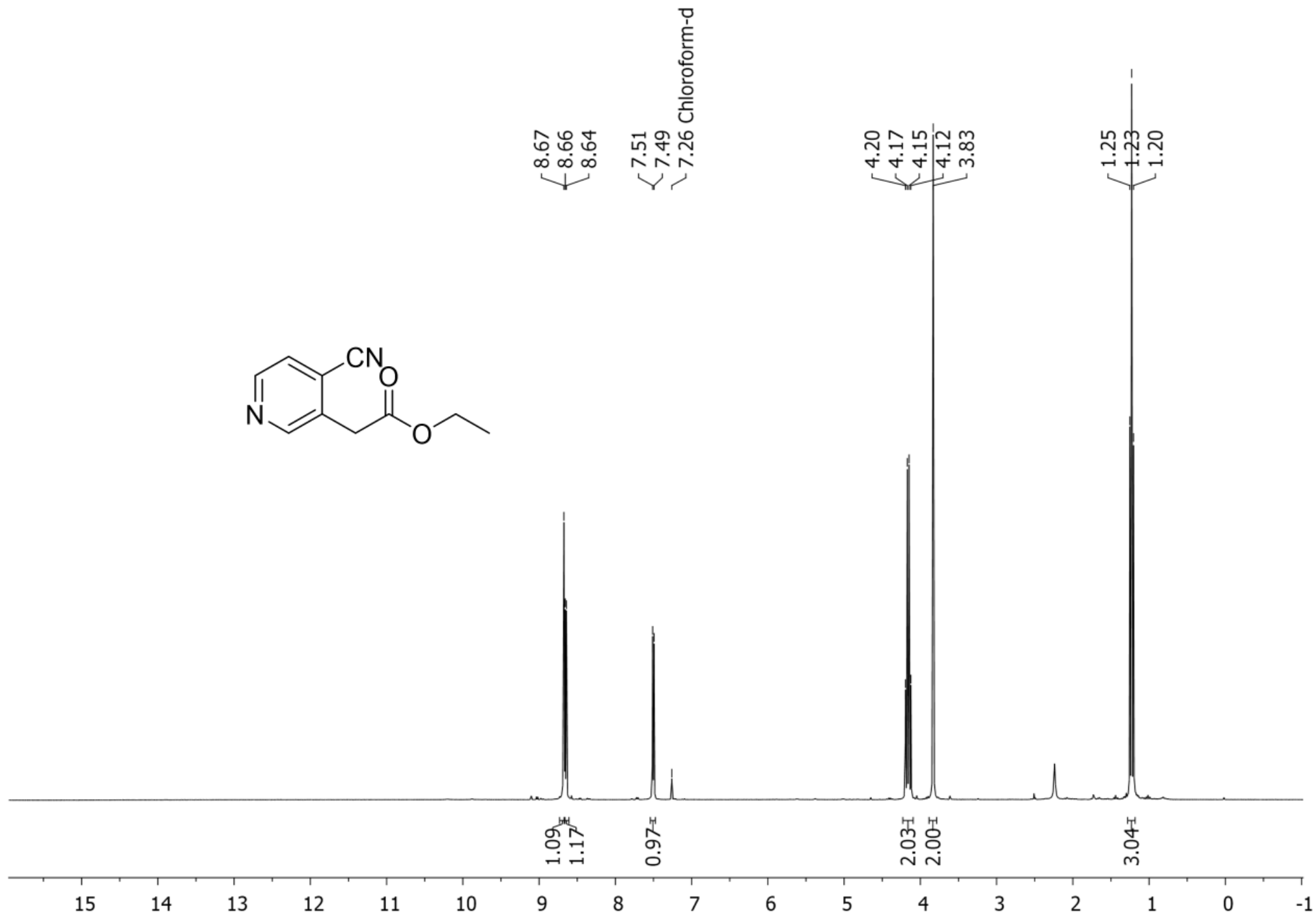

^{13}*C NMR (75 MHz, CDCl$_3$) spectrum of Ethyl 2-(4-cyanopyridin-3-yl)acetate (29)*

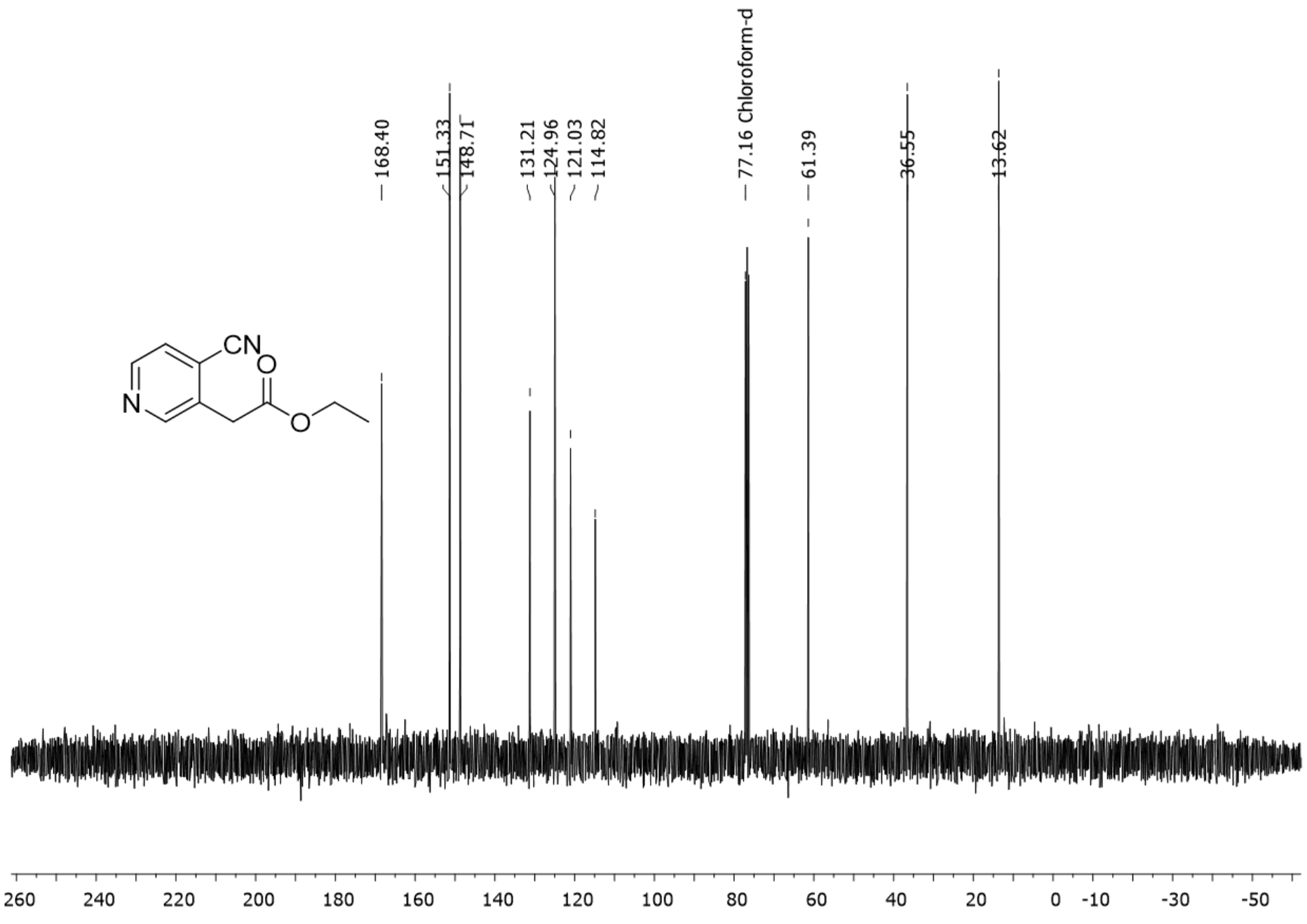

^{1}H NMR (300 MHz, CDCl3) spectrum of Ethyl 2-(3-cyanopyrazin-2-yl)acetate

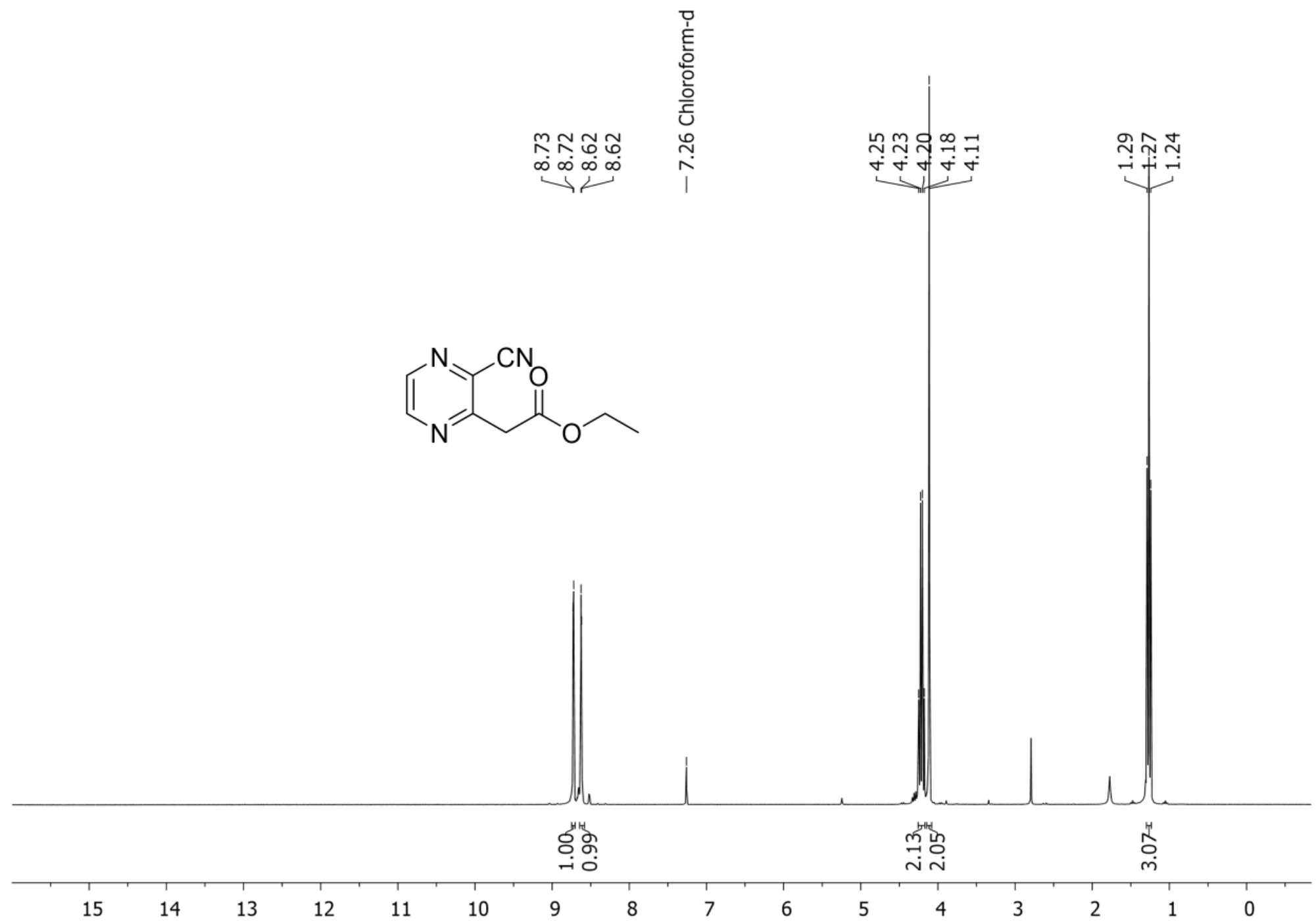

^{1}H NMR (75 MHz, CDCl3) spectrum of Ethyl 2-(3-cyanopyrazin-2-yl)acetate

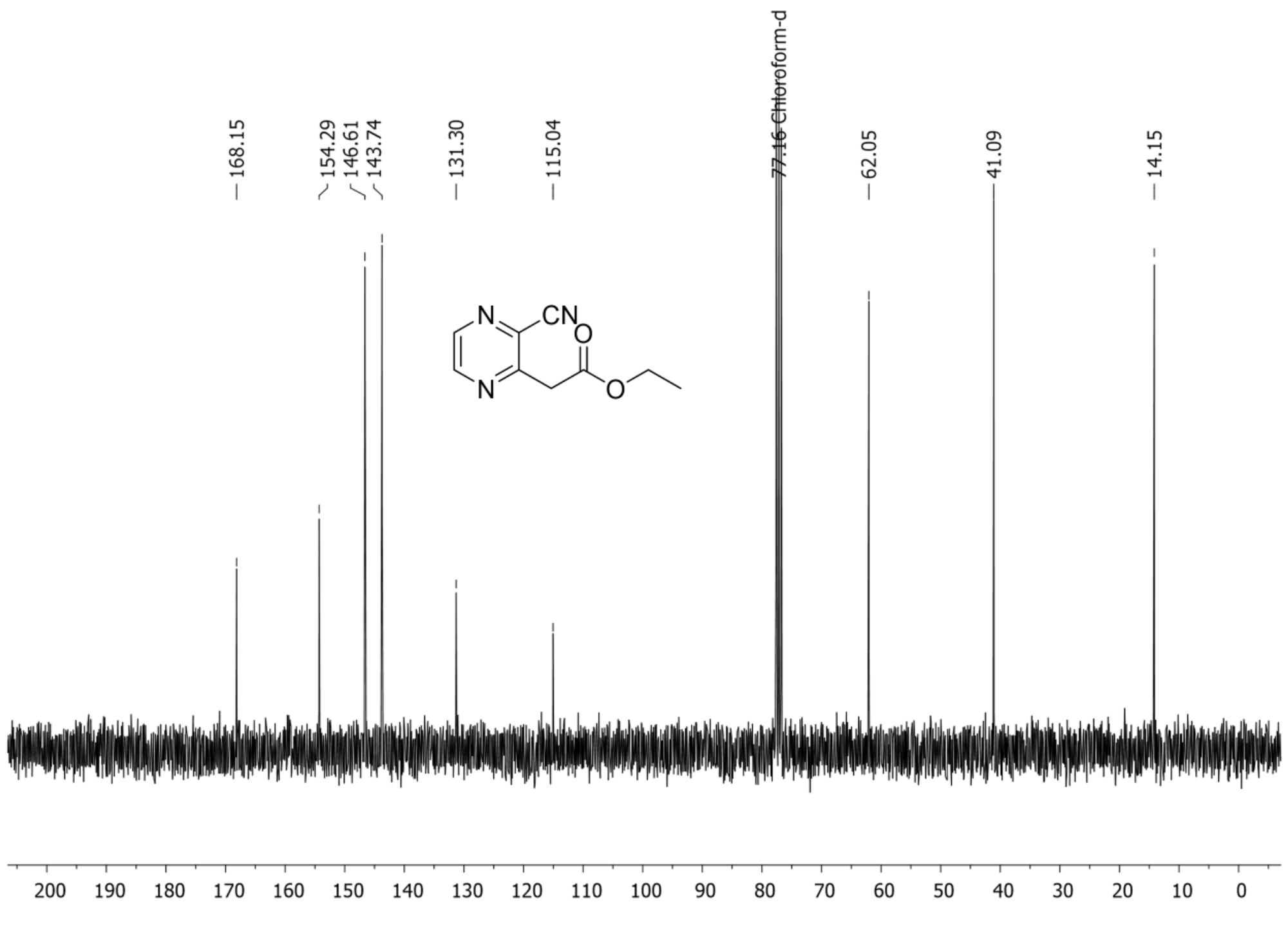

^{1}H NMR (300 MHz, DMSO-d6) spectrum of 5,8-dihydropyrido[4,3-c] pyridazin-7(6H)-one (4)

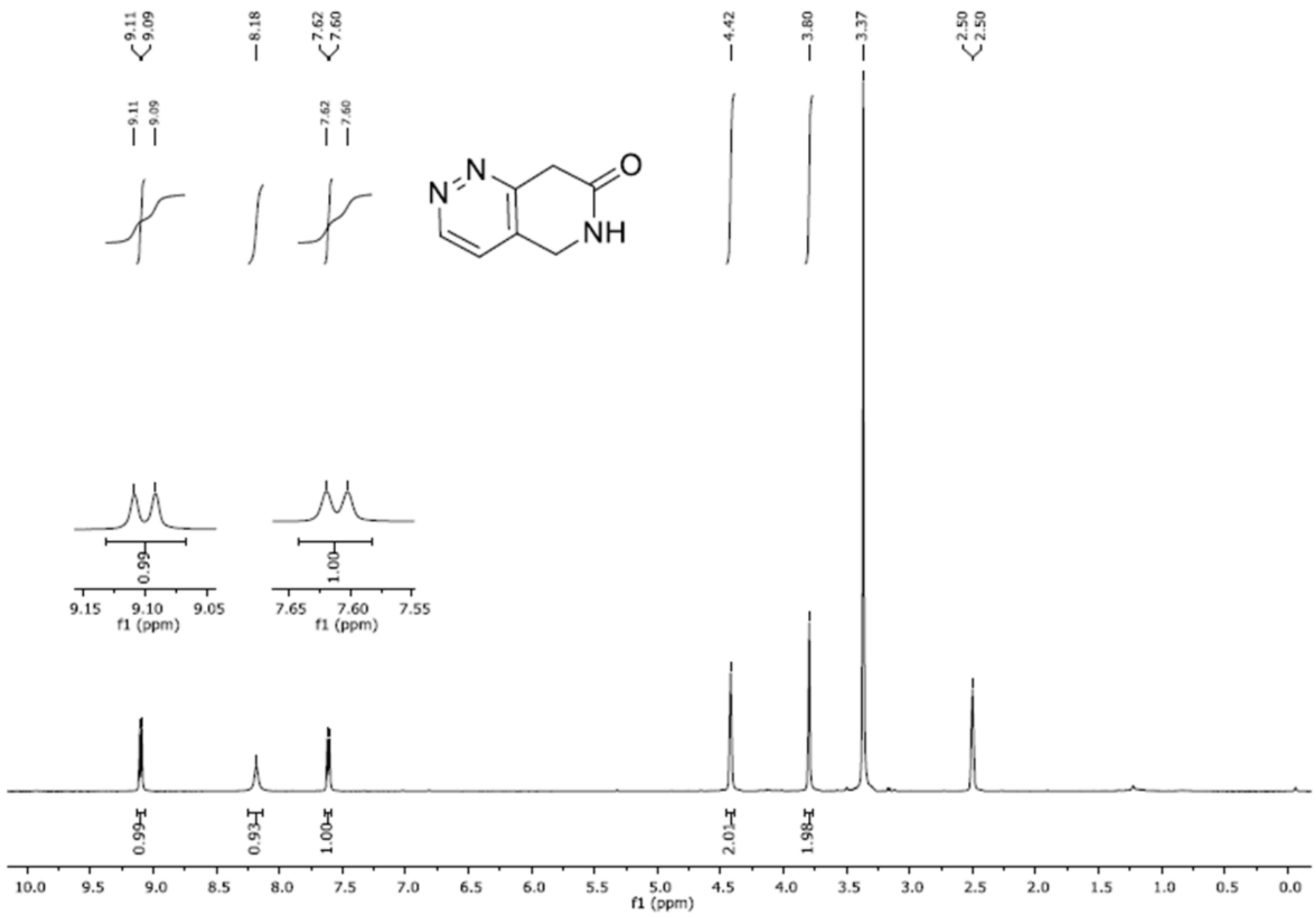

^{13}C NMR (75 MHz, DMSO-d6) spectrum of 5,8-dihydropyrido[4,3-c] pyridazin-7(6H)-one (4)

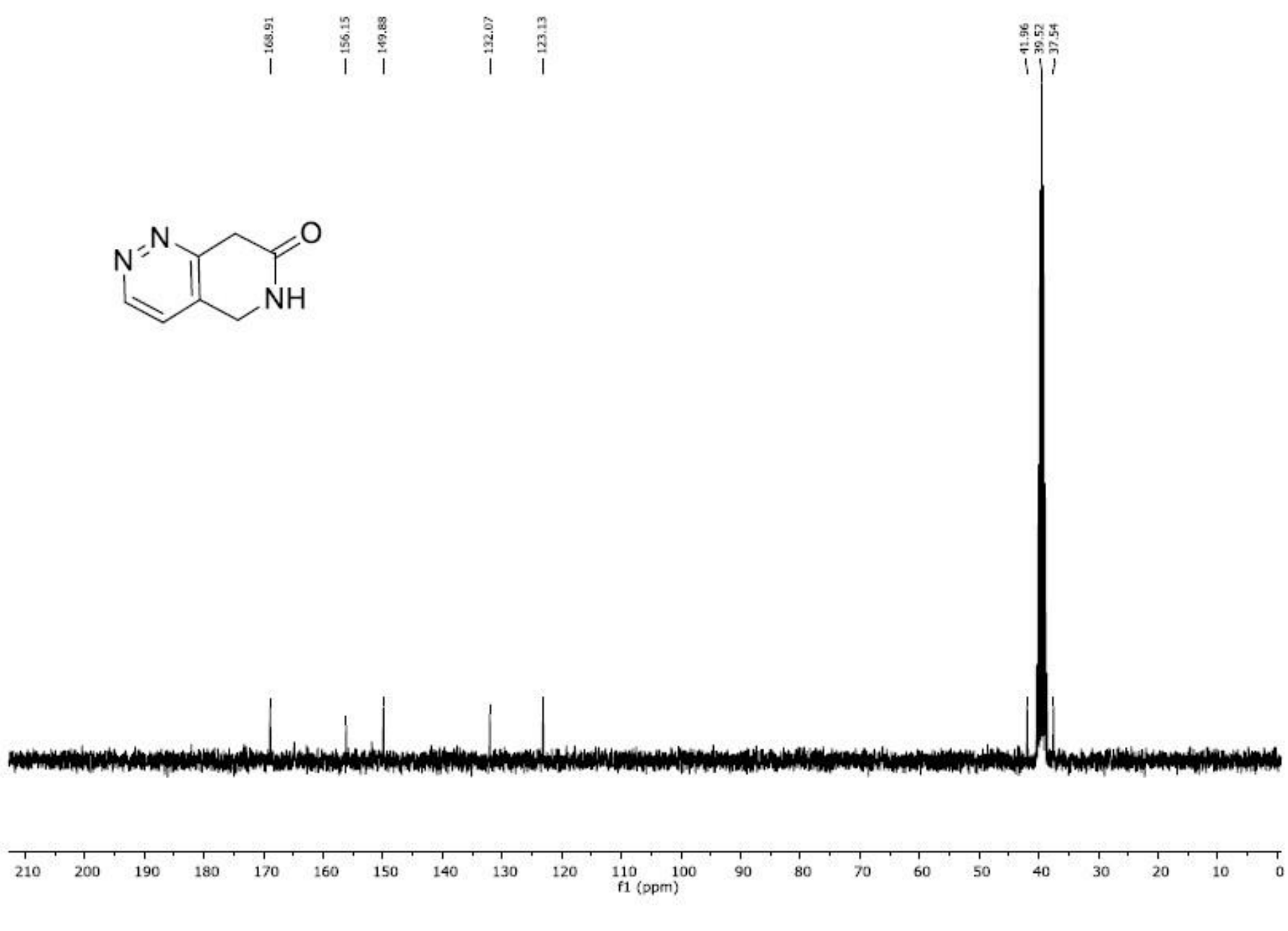

^{1}H NMR (300 MHz, (DMSO) spectrum of 3,4-dimethyl-5,8-dihydropyrido[4,3-c]pyridazin-7(6H)-one (5)

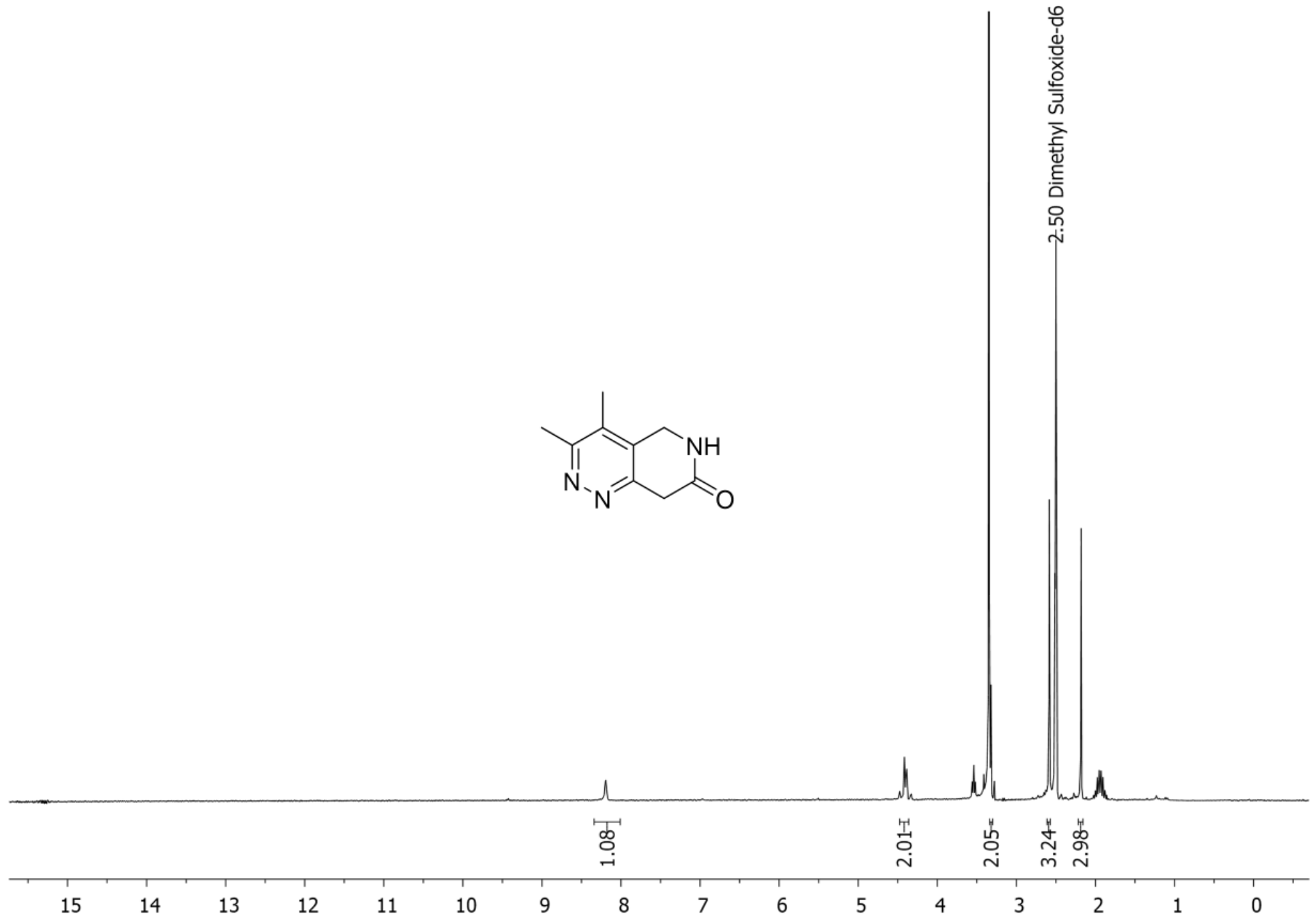

^{13}C NMR (75 MHz, (DMSO) spectrum of 3,4-dimethyl-5,8-dihydropyrido[4,3-c]pyridazin-7(6H)-one (5)

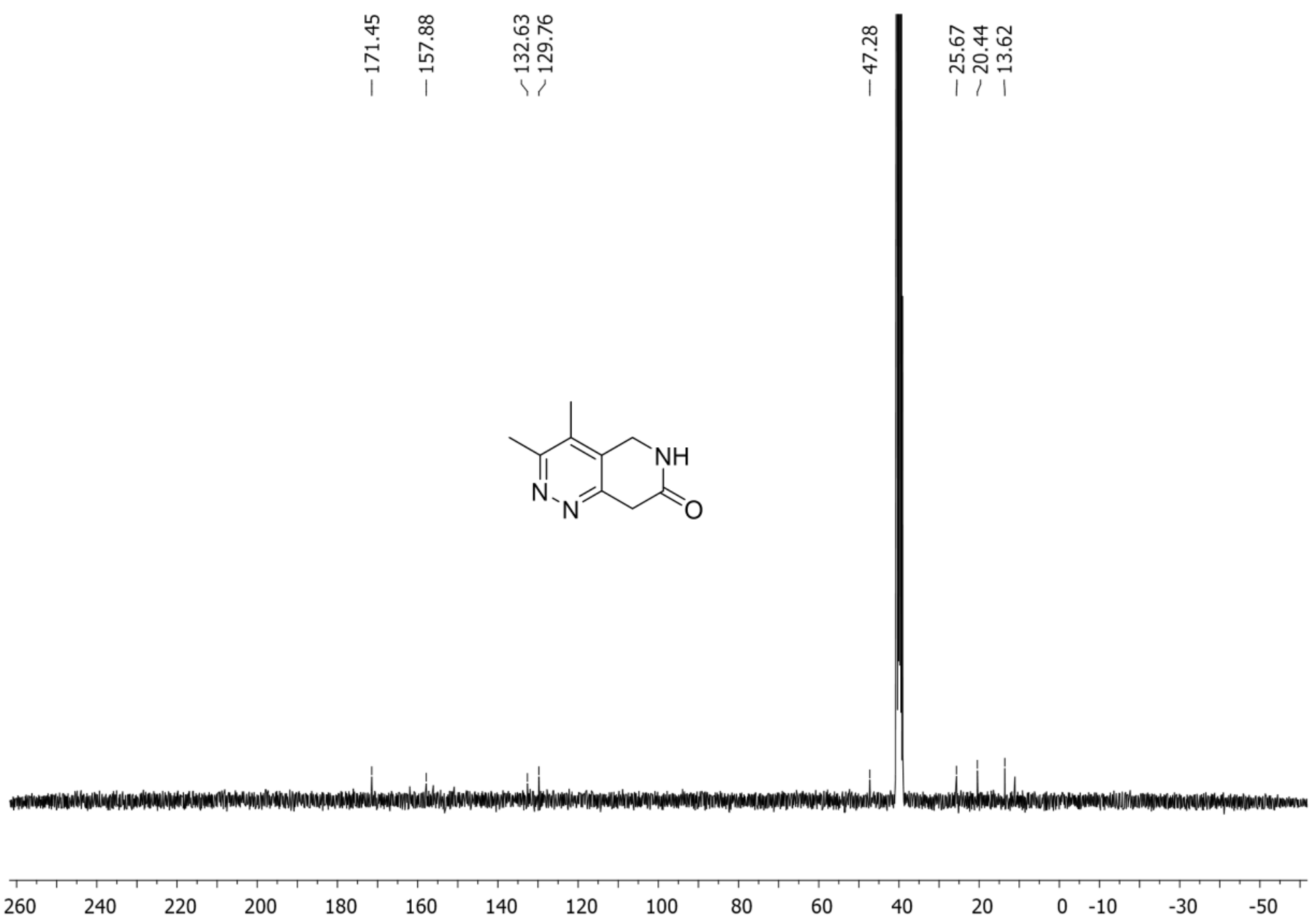

^{1}H NMR (300 MHz, $(CD_3)_2CO$) spectrum of 5,8-dihydro-1,6-naphthyridin-7(6H)-one (6)

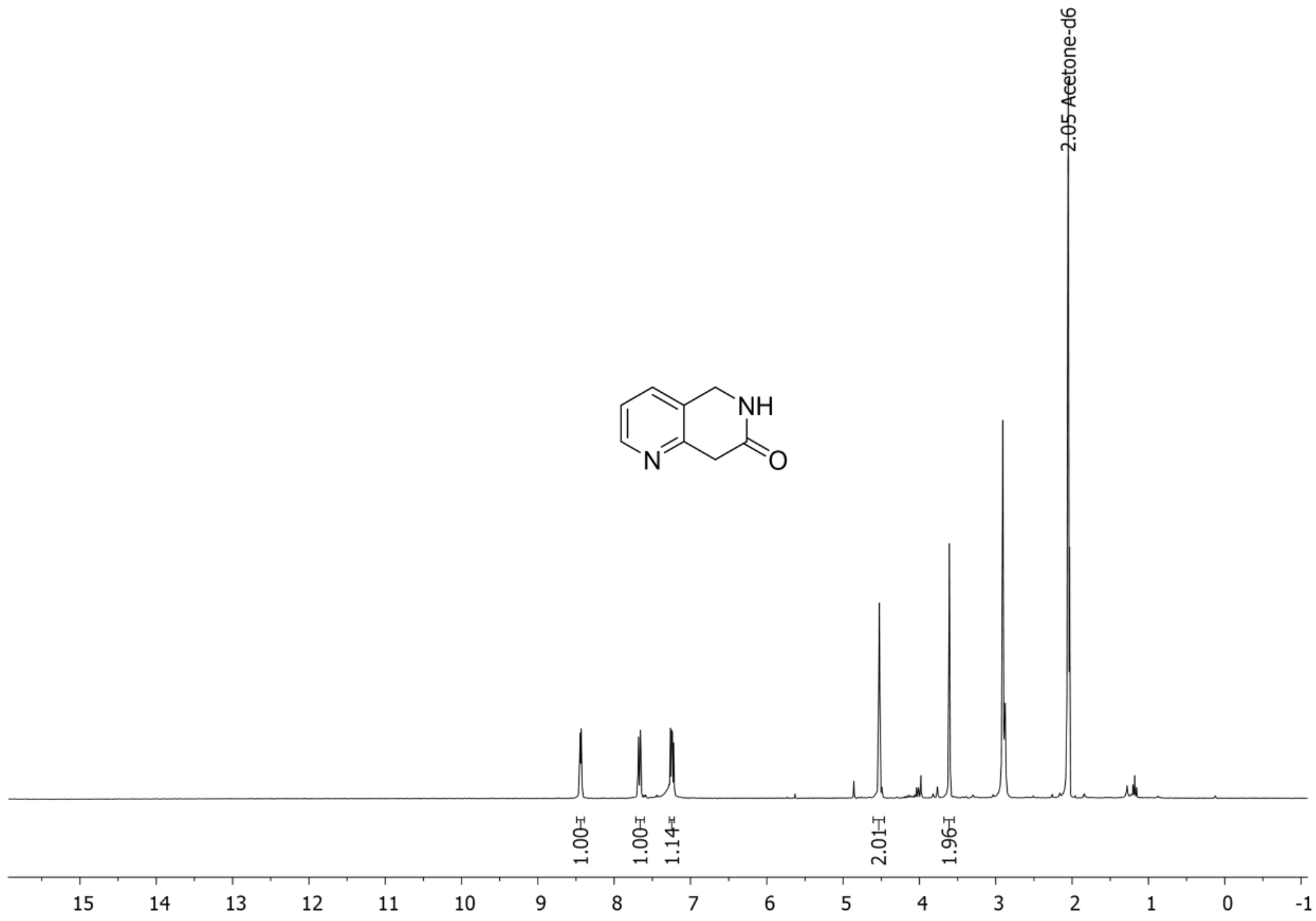

^{13}C NMR (75 MHz, $(CD_3)_2CO$) spectrum of 5,8-dihydro-1,6-naphthyridin-7(6H)-one (6)

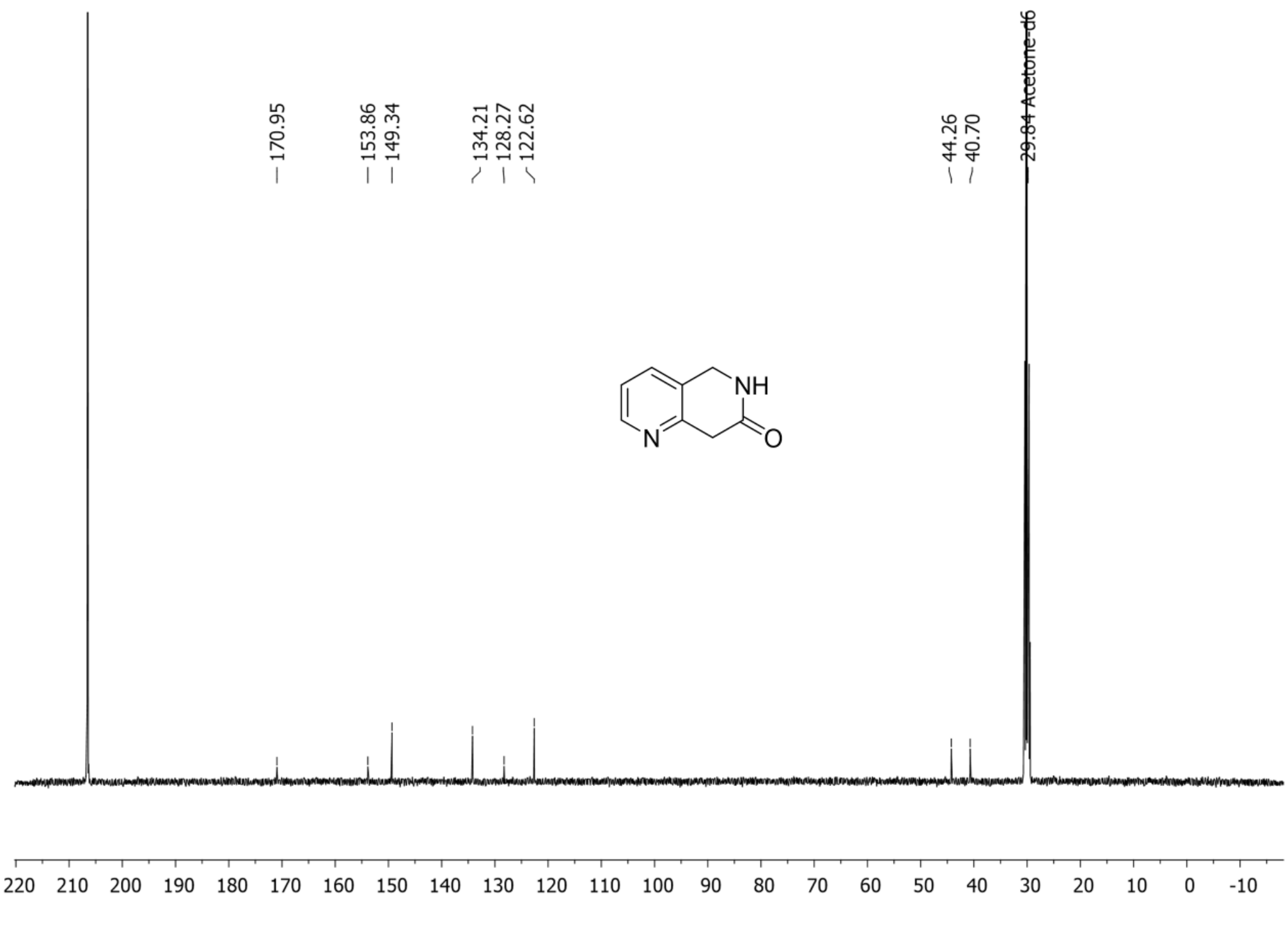

^{1}H NMR (300 MHz, $(CD_3)_2CO$) spectrum of 1,4-dihydro-2,6-naphthyridin-3(2H)-one (1)

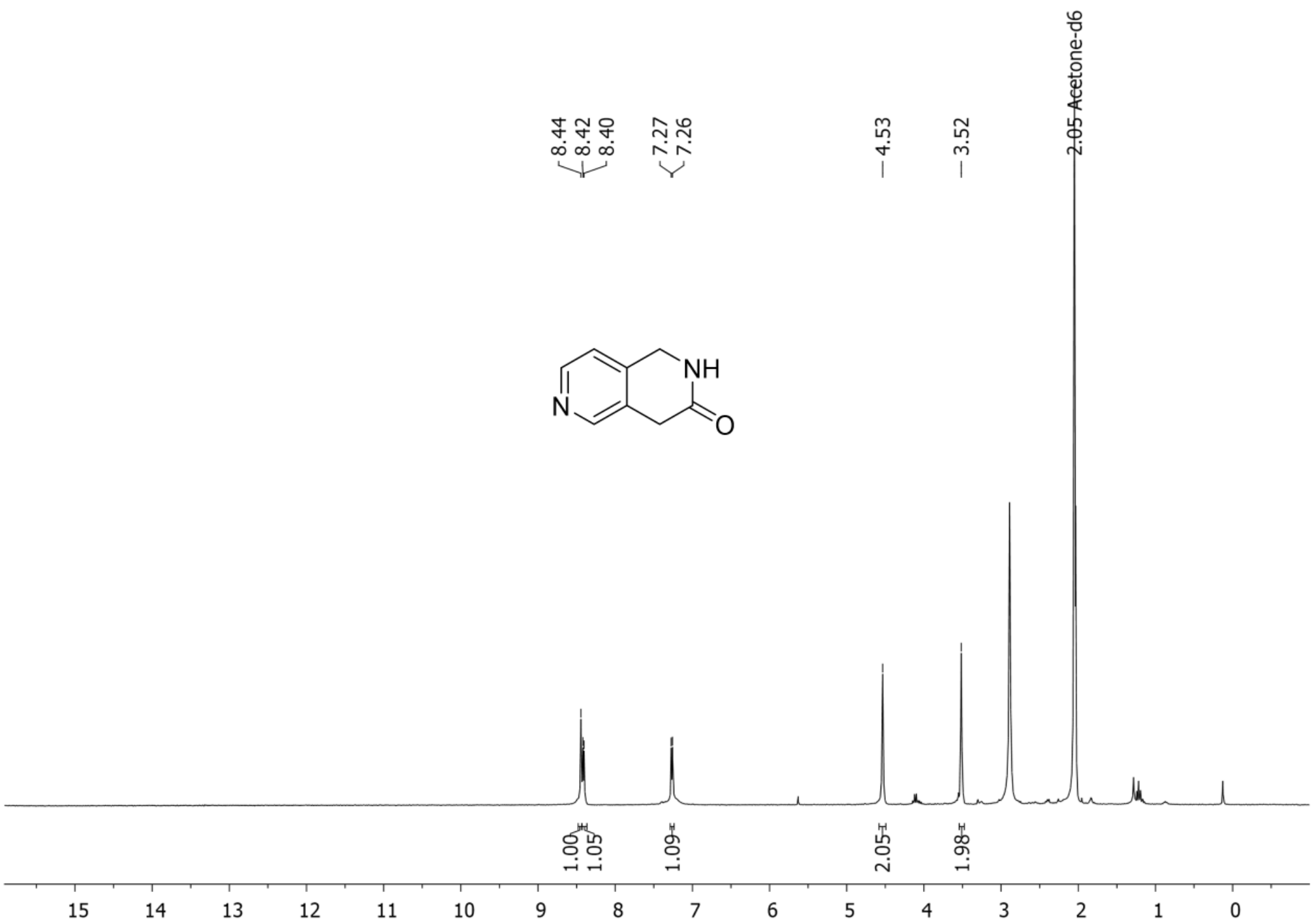

^{1}H NMR (75 MHz, $(CD_3)_2CO$) spectrum of 1,4-dihydro-2,6-naphthyridin-3(2H)-one (1)

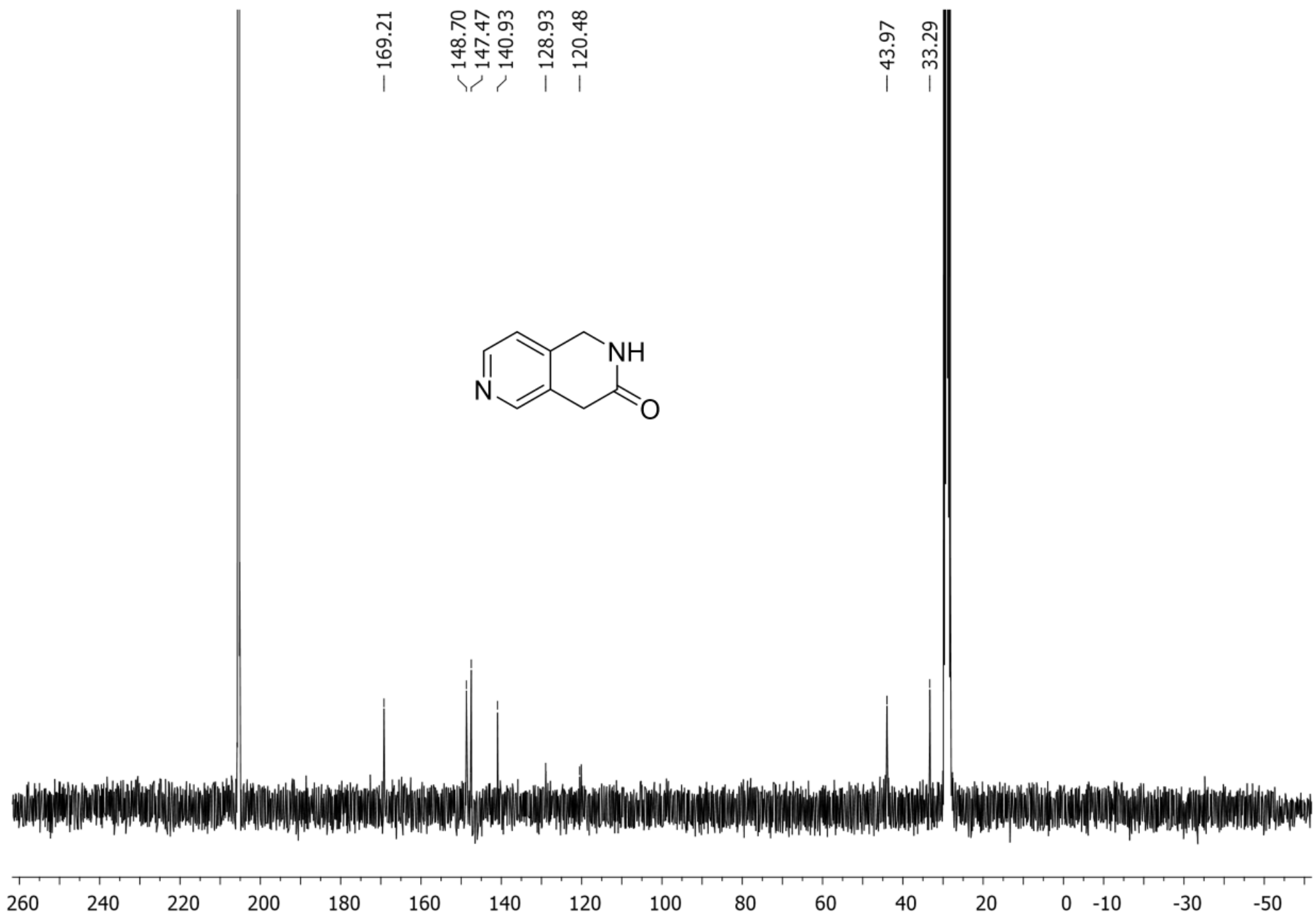

^{1}H NMR (300 MHz, $(CD_3)_2CO$) spectrum of 1,4-dihydro-2,7-naphthyridin-3(2H)-one (8)

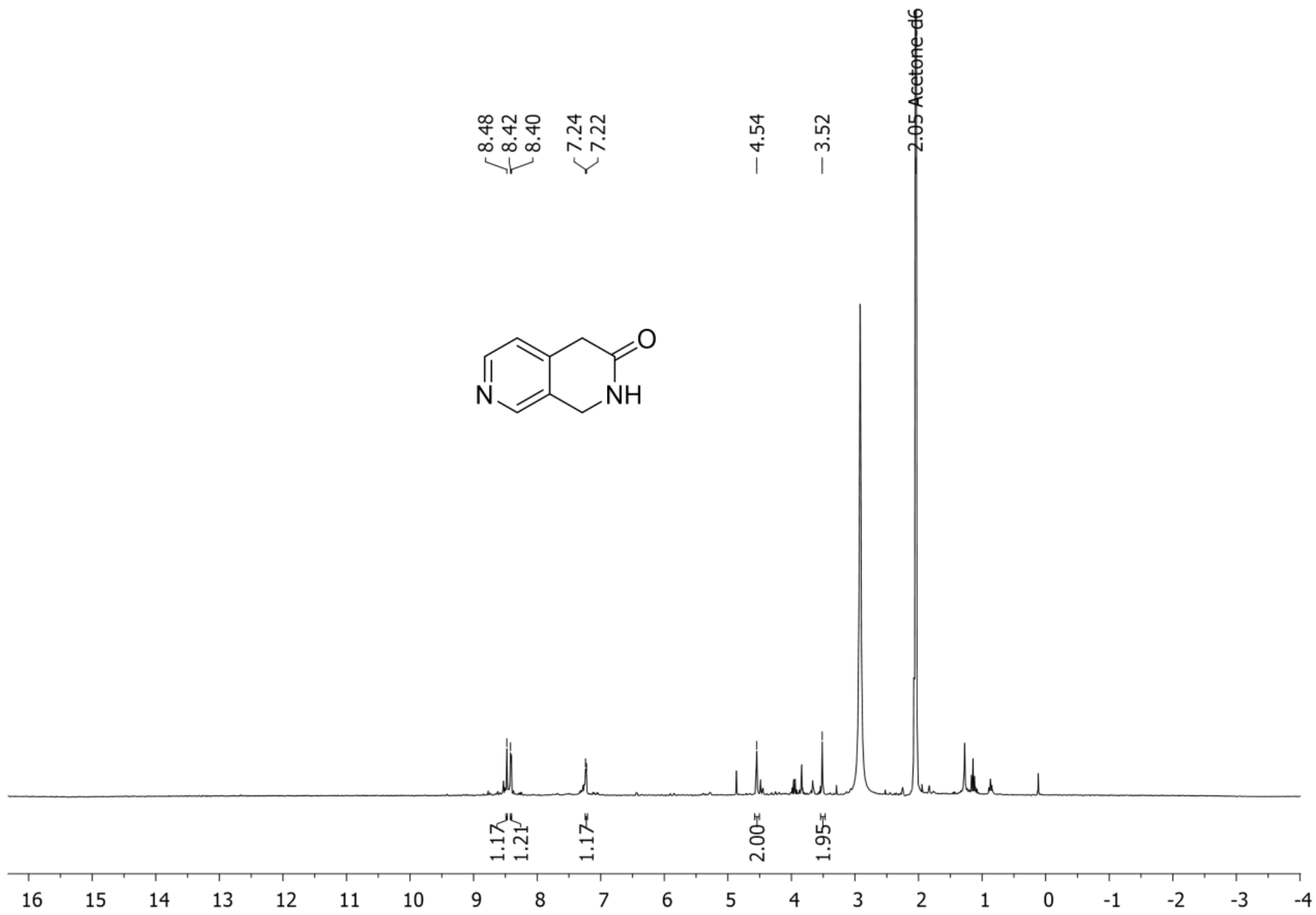

^{1}H NMR (300 MHz, $(CD_3)_2CO$) spectrum of 5,8-dihydropyrido[3,4-b]pyrazin-7(6H)-one(9)

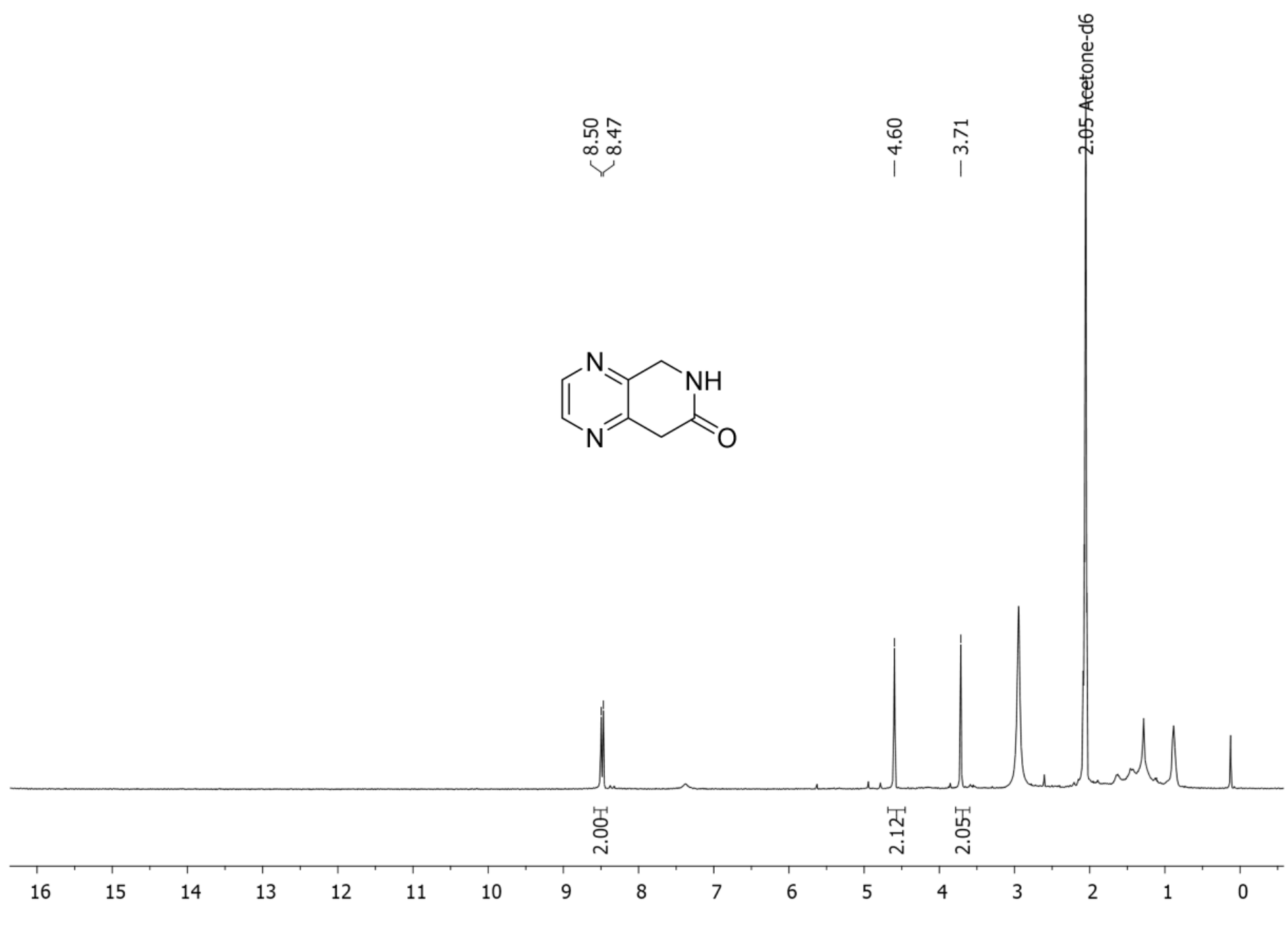

^{13}C NMR (75 MHz, (CD $_3$)$_2$ CO) spectrum of 5,8-dihydropyrido[3,4-b]pyrazin-7(6H)-one(9)

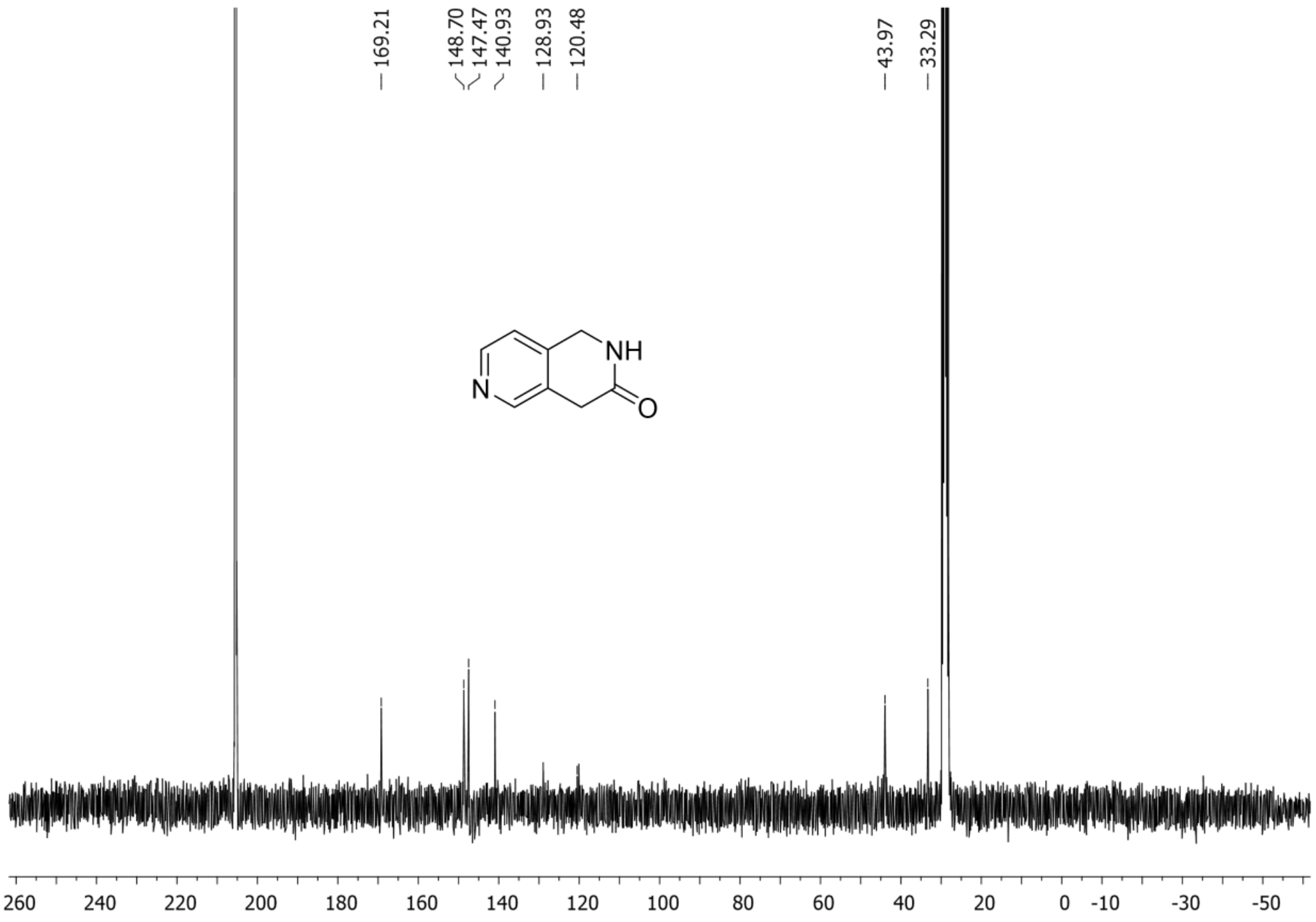

^{1}H NMR (300 MHz, (CD $_3$)$_2$ CO) spectrum of ethyl 7-oxo-5,6,7,8-tetrahydro-1,6-naphthyridine-8-carboxylate (31)

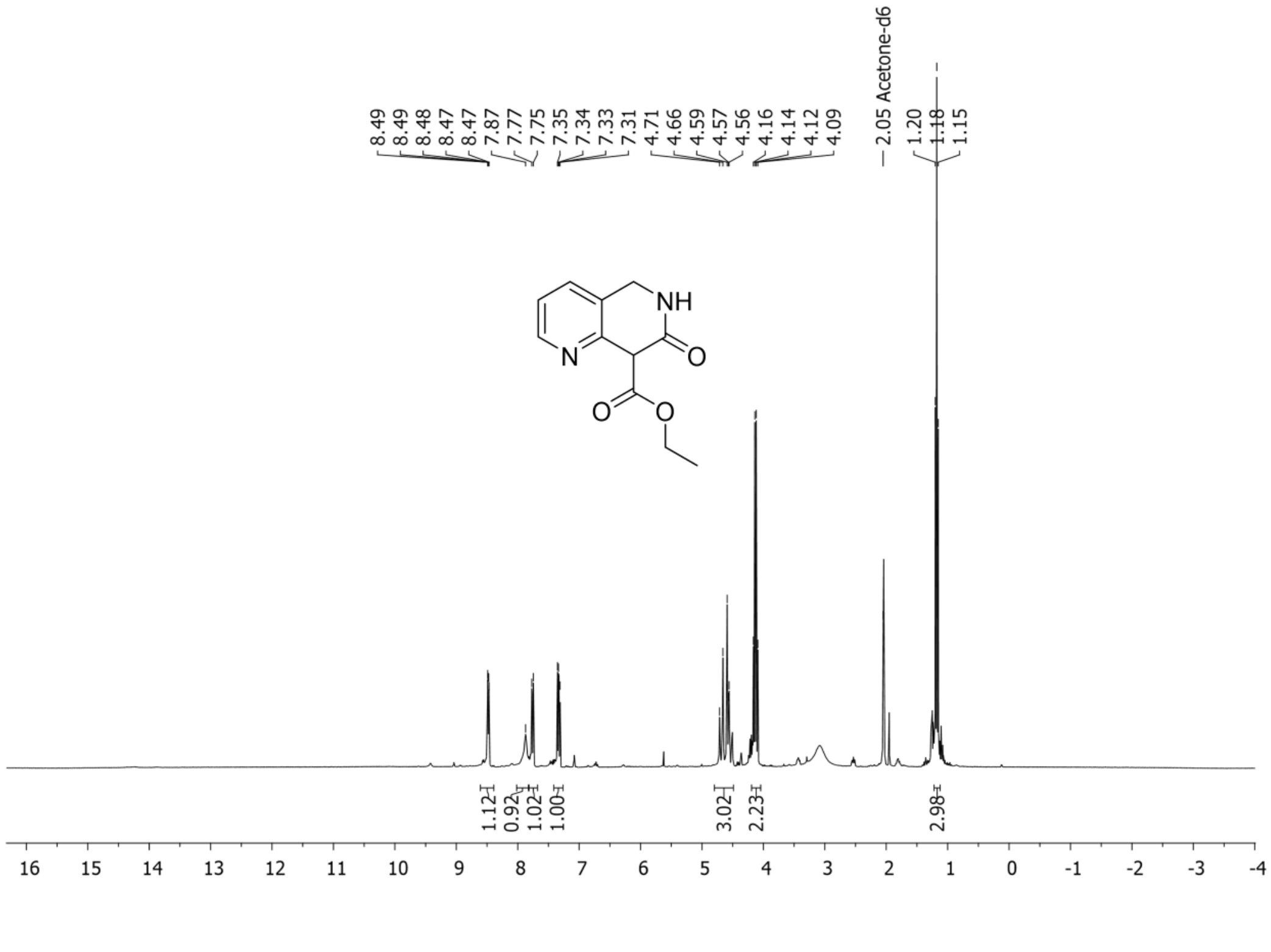

^{13}C NMR (75 MHz, $(CD_3)_2CO$) spectrum of ethyl 7-oxo-5,6,7,8-tetrahydro-1,6-naphthyridine-8-carboxylate (31)

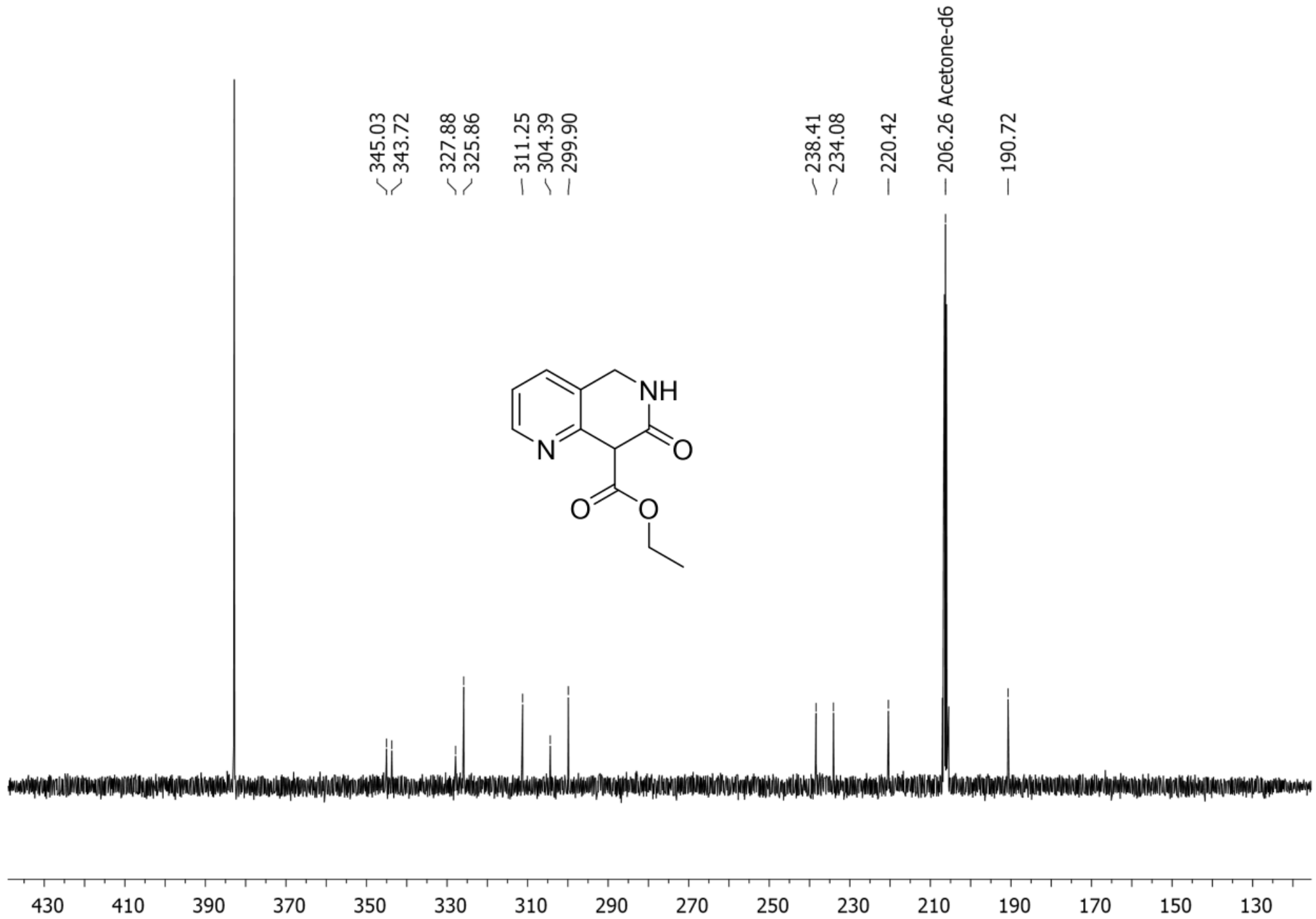

^{1}H NMR (300 MHz, $(CD_3)_2CO$) spectrum of ethyl 3-oxo-1,2,3,4-tetrahydro-2,6-naphthyridine-4-carboxylate (32)

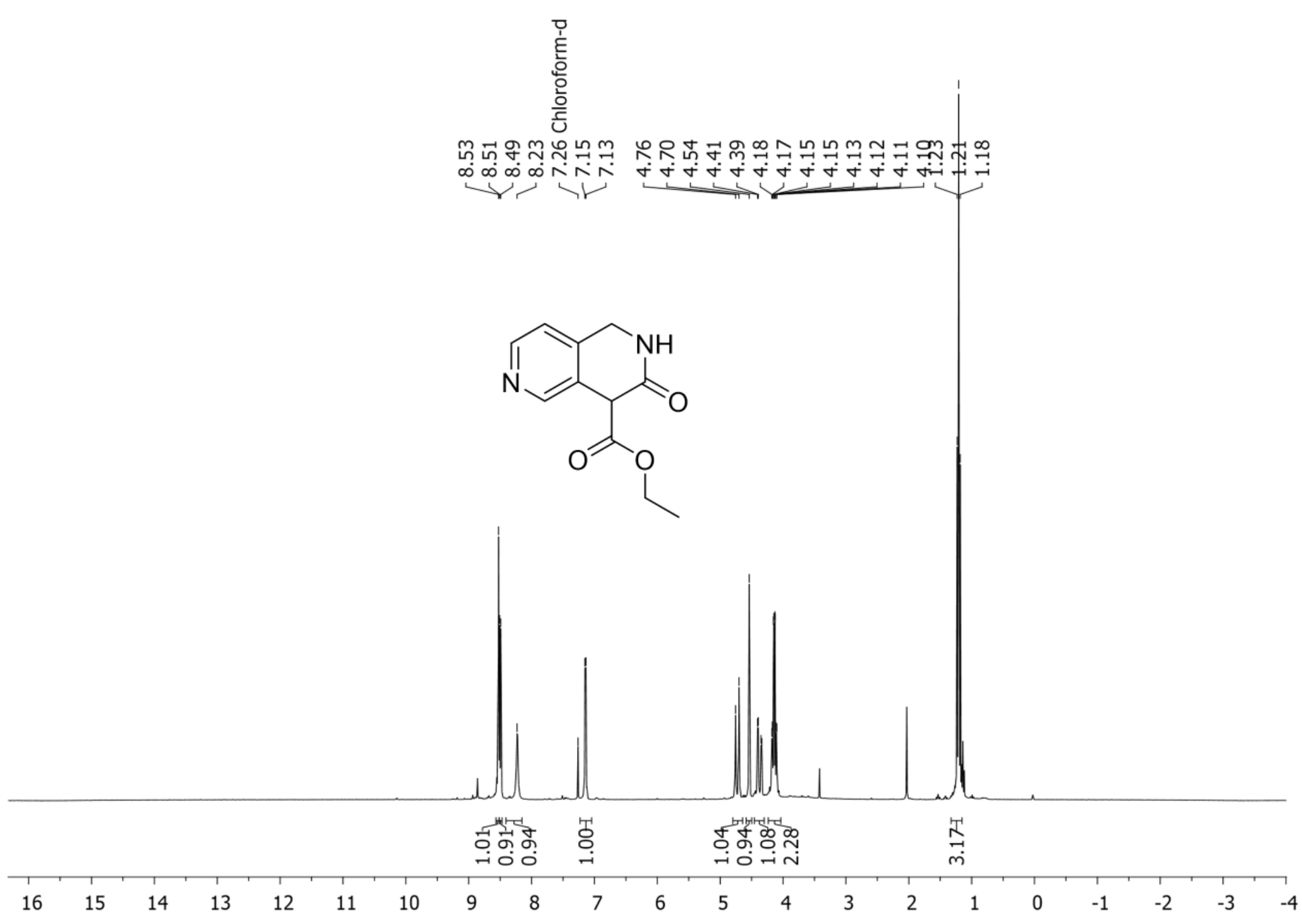

^{13}C NMR (75 MHz, $(CD_3)_2CO$) spectrum of ethyl 3-oxo-1,2,3,4-tetrahydro-2,6-naphthyridine-4-carboxylate (32)

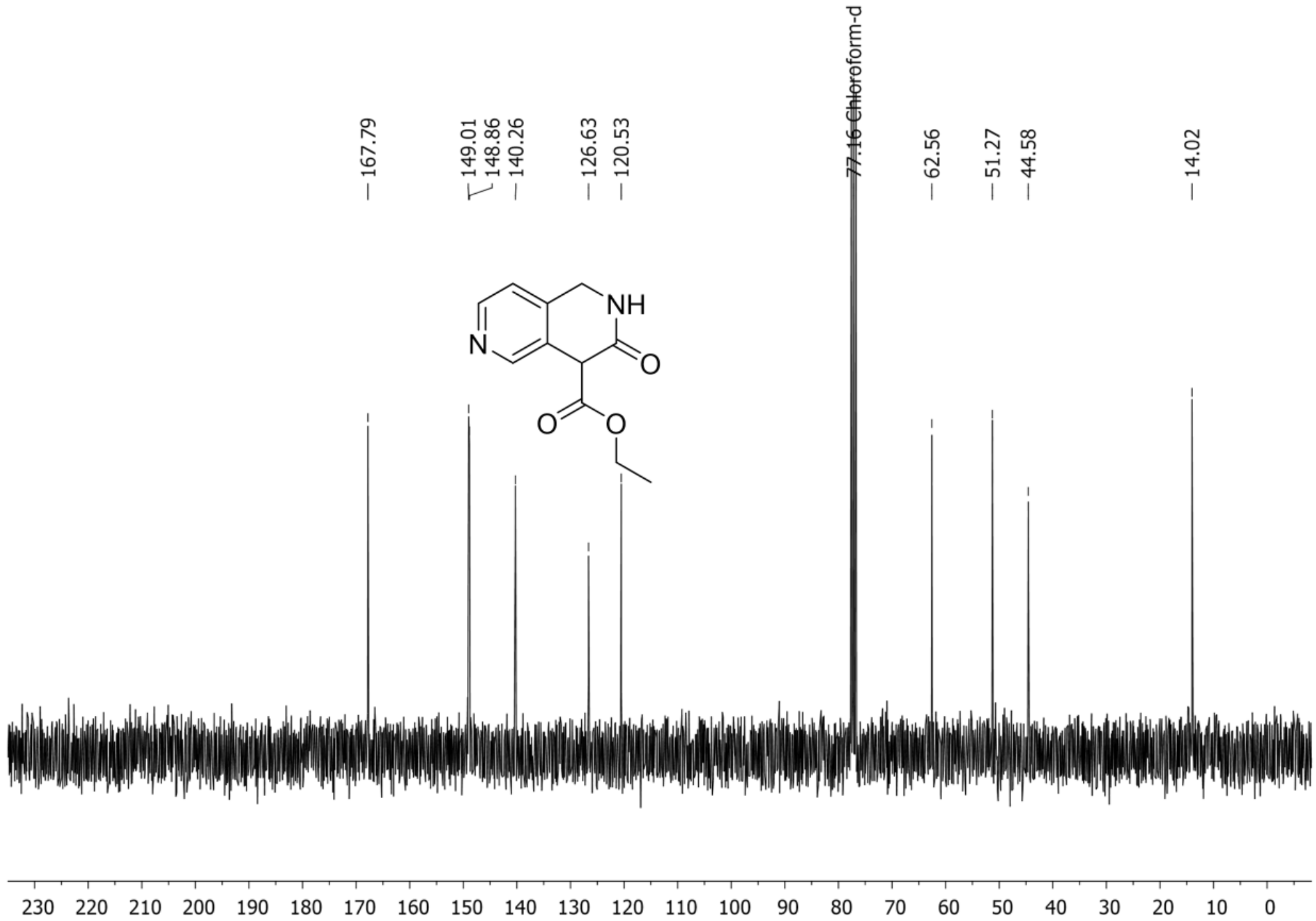

^{1}H NMR (75 MHz, (CD 3)2 CO) spectrum of ethyl 3-oxo-1,2,3,4-tetrahydro-2,7-naphthyridine-4-carboxylate 33

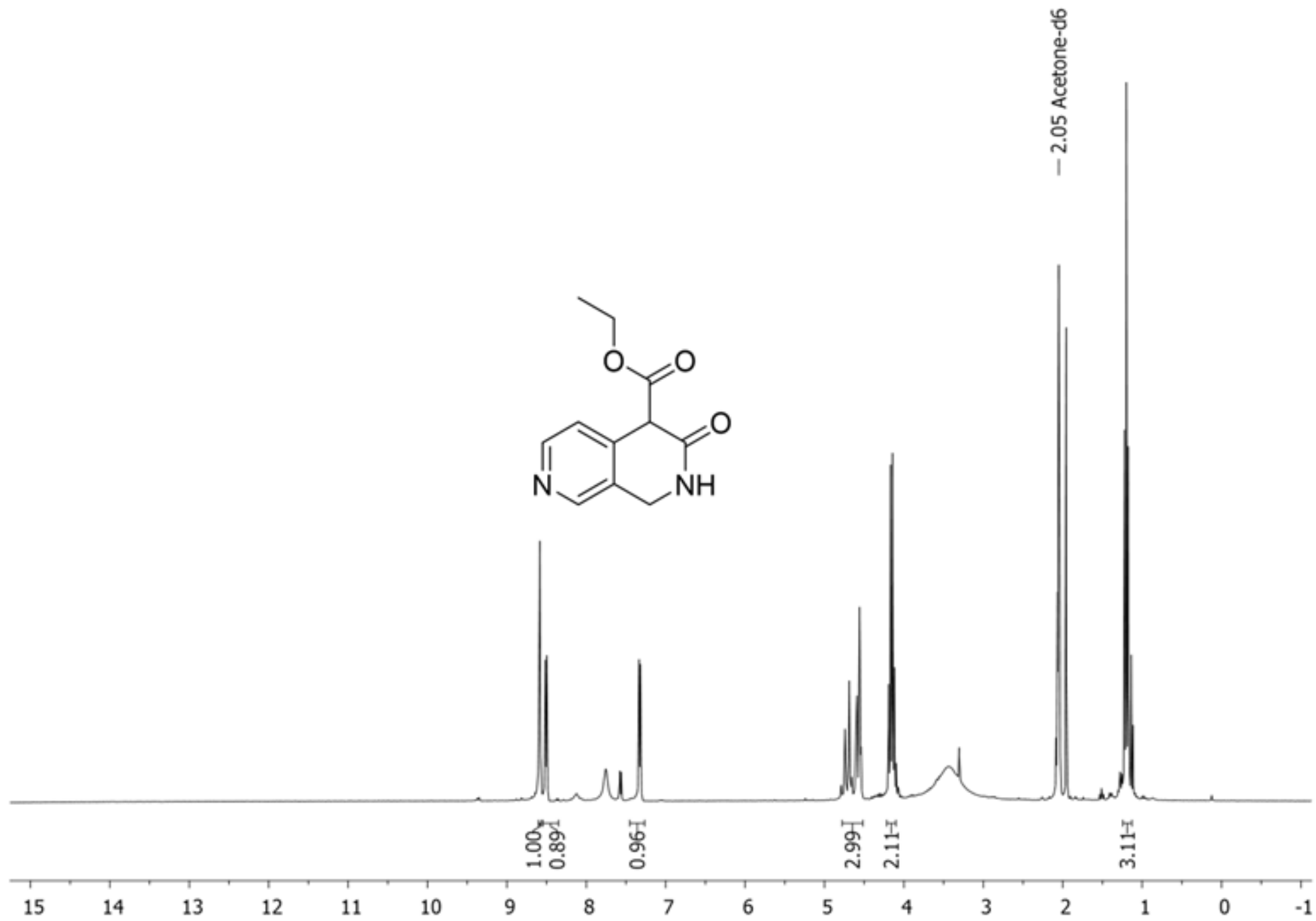

^{13}C *NMR (75 MHz, $(CD_3)_2CO$) spectrum of ethyl 3-oxo-1,2,3,4-tetrahydro-2,7-naphthyridine-4-carboxylate (33)*

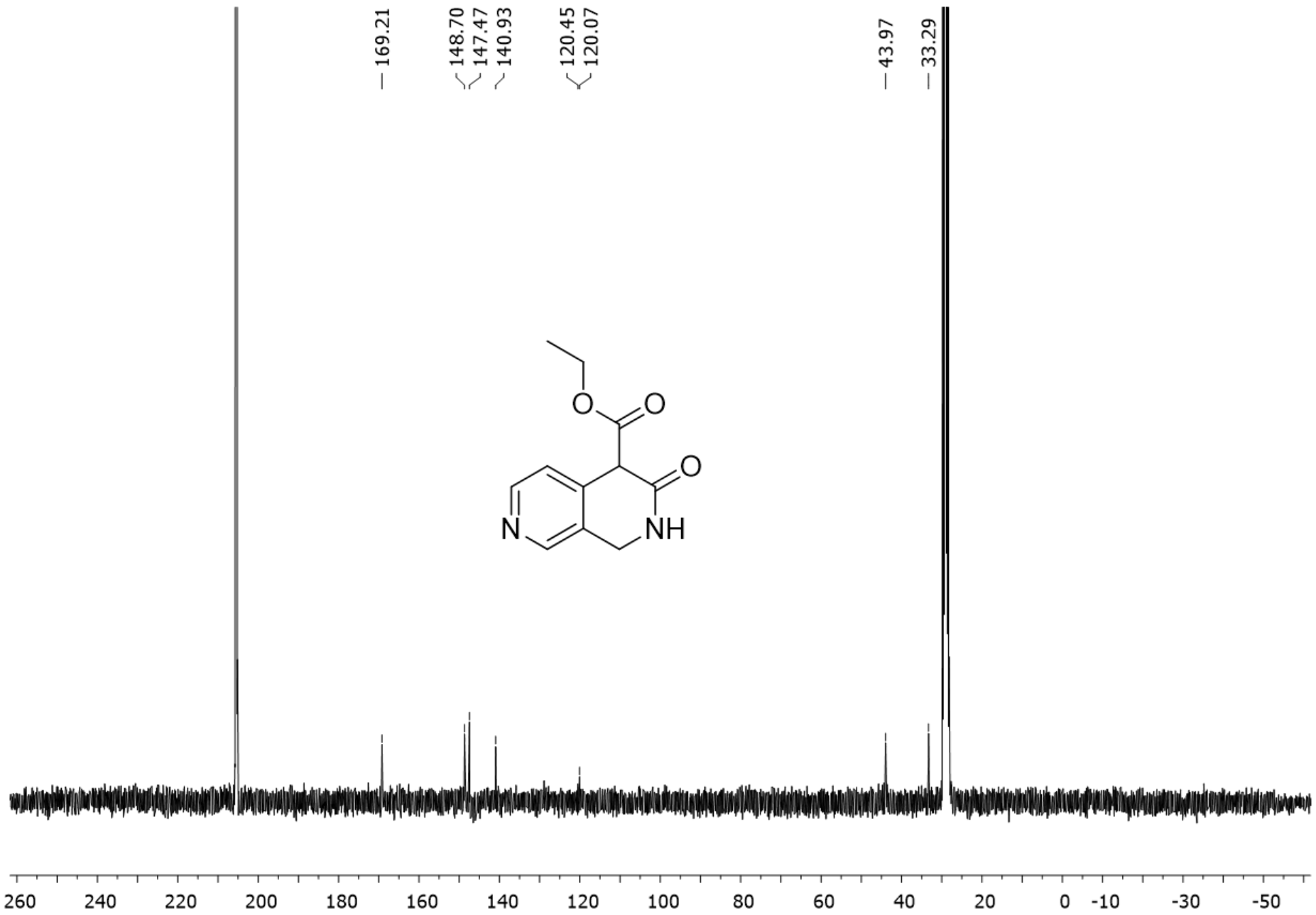

^{1}H *NMR (300 MHz, $(CD_3)_2CO$) spectrum of Ethyl-7-oxo-5,6,7,8-tetrahydropyrido[3,4-b]pyrazine-8-carboxylate (34)*

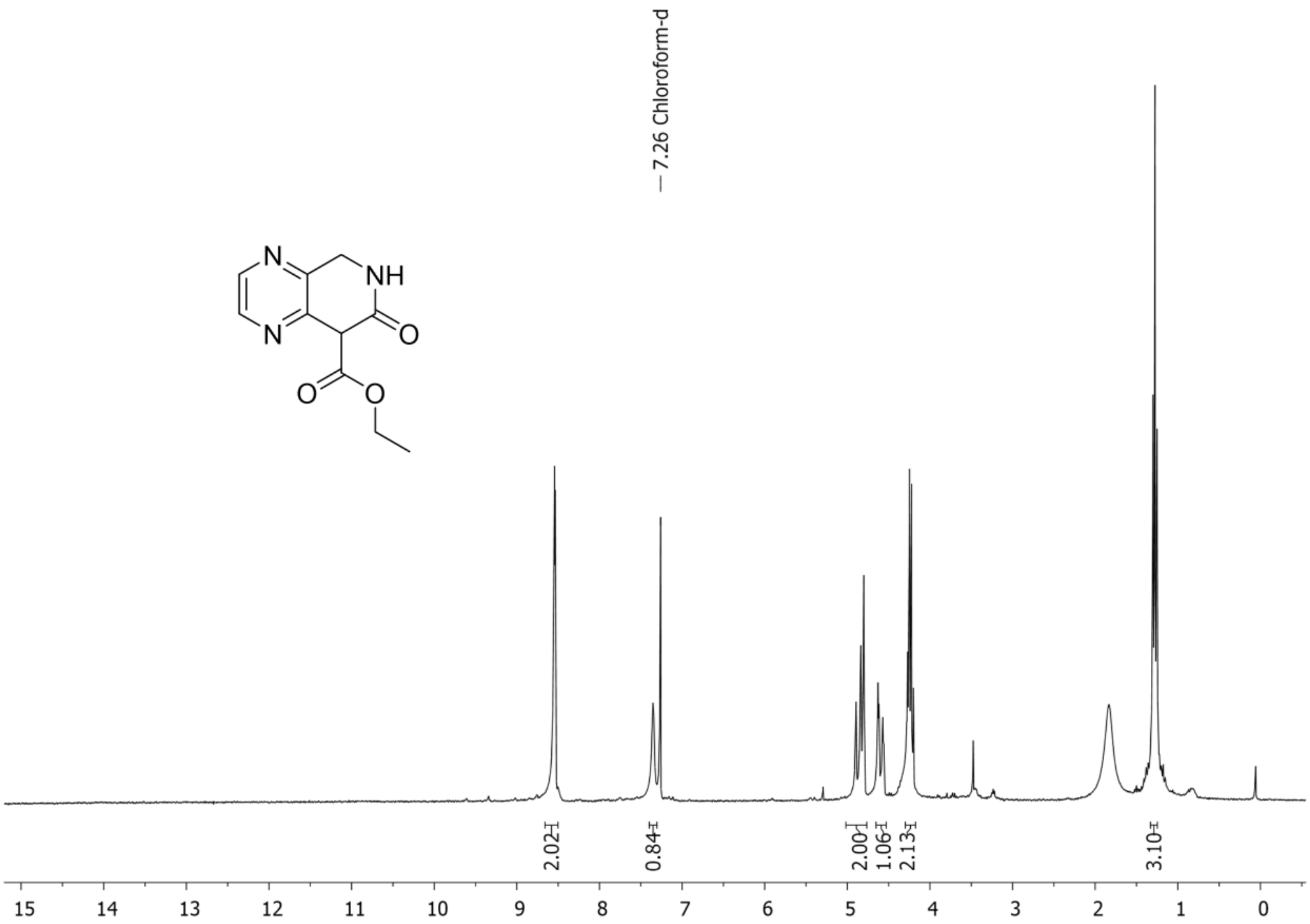

^{13}C NMR (75 MHz, (CD $_3$)$_2$ CO) spectrum of Ethyl-7-oxo-5,6,7,8-tetrahydropyrido[3,4-b]pyrazine-8-carboxylate (34)

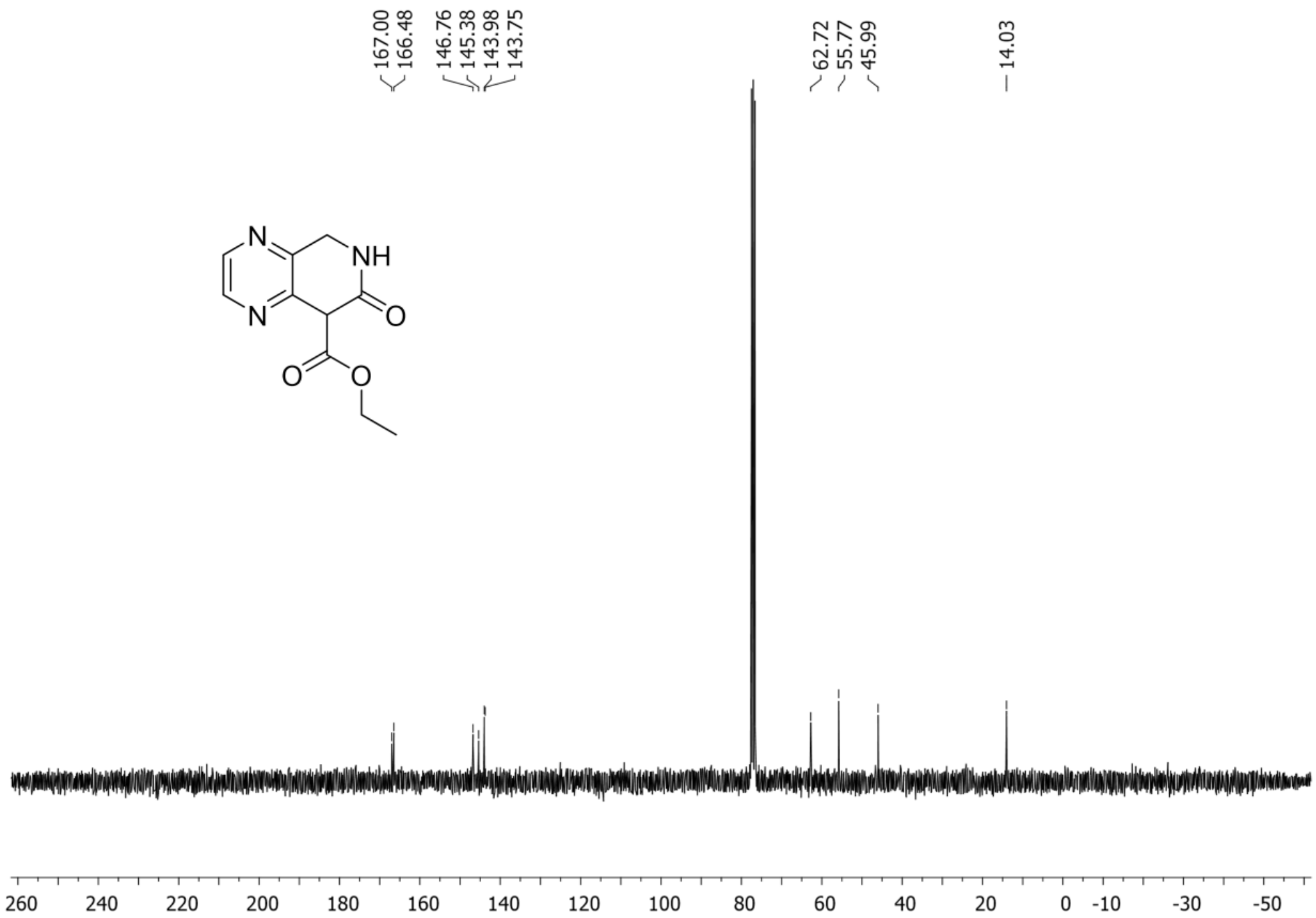

^{1}H NMR (300 MHz, DMSO) spectrum of 1,6-naphthyridin-7-ol (35)

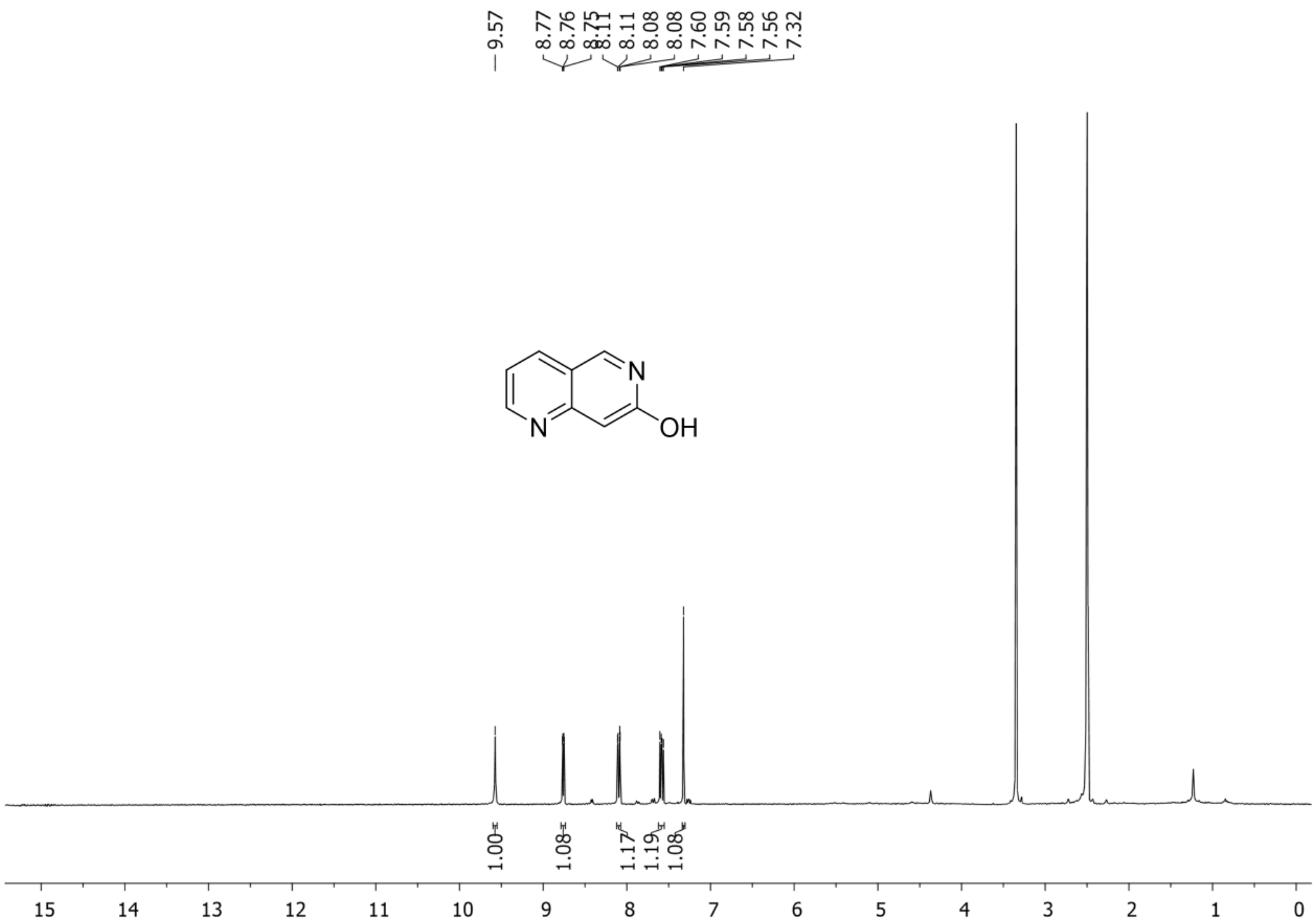

^{13}C NMR (75 MHz, DMSO) spectrum of 1,6-naphthyridin-7-ol

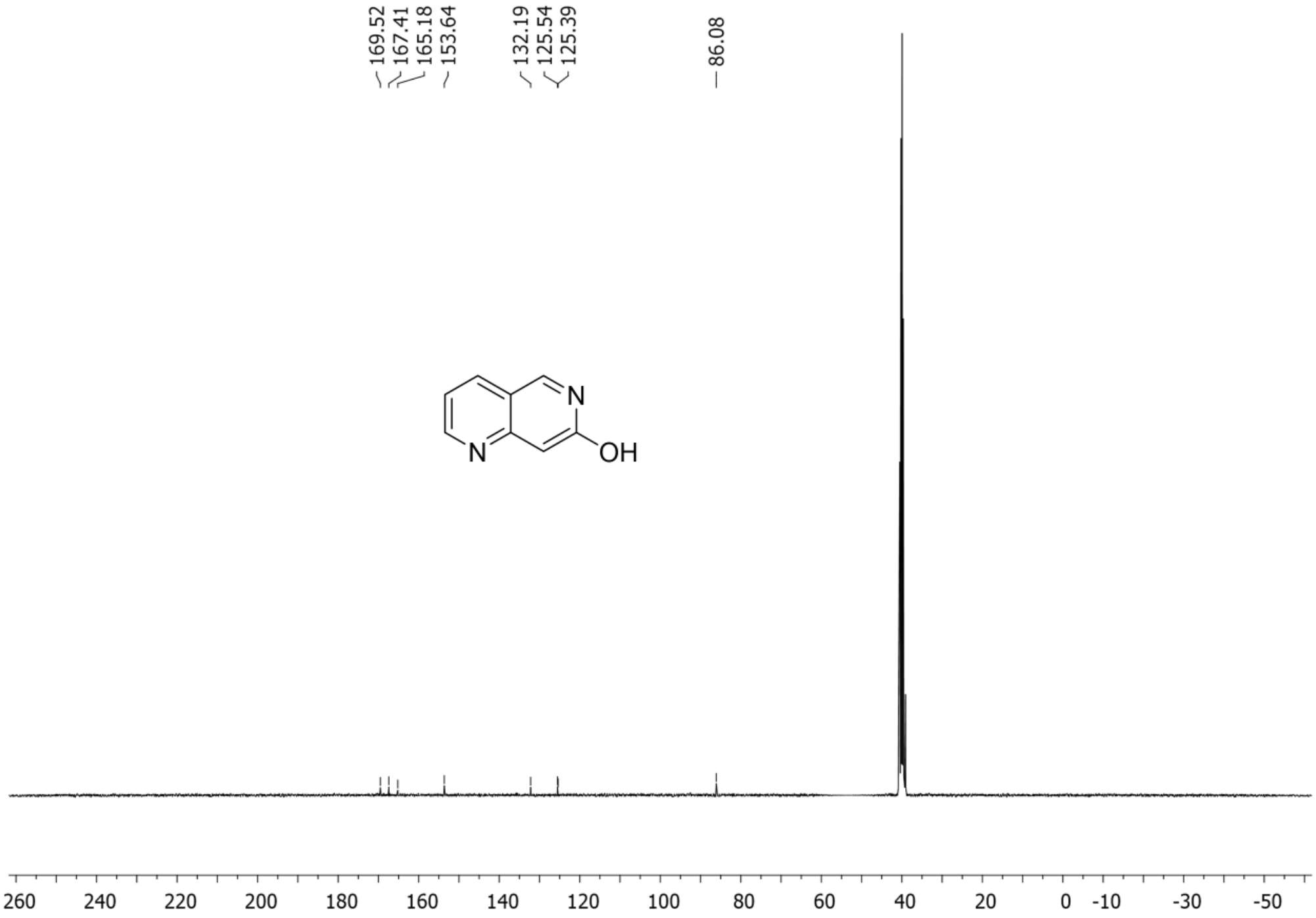

^{1}H-NMR – 400 MHz in DMSO-d6: 2,6-naphthyridin-3-ol (2)

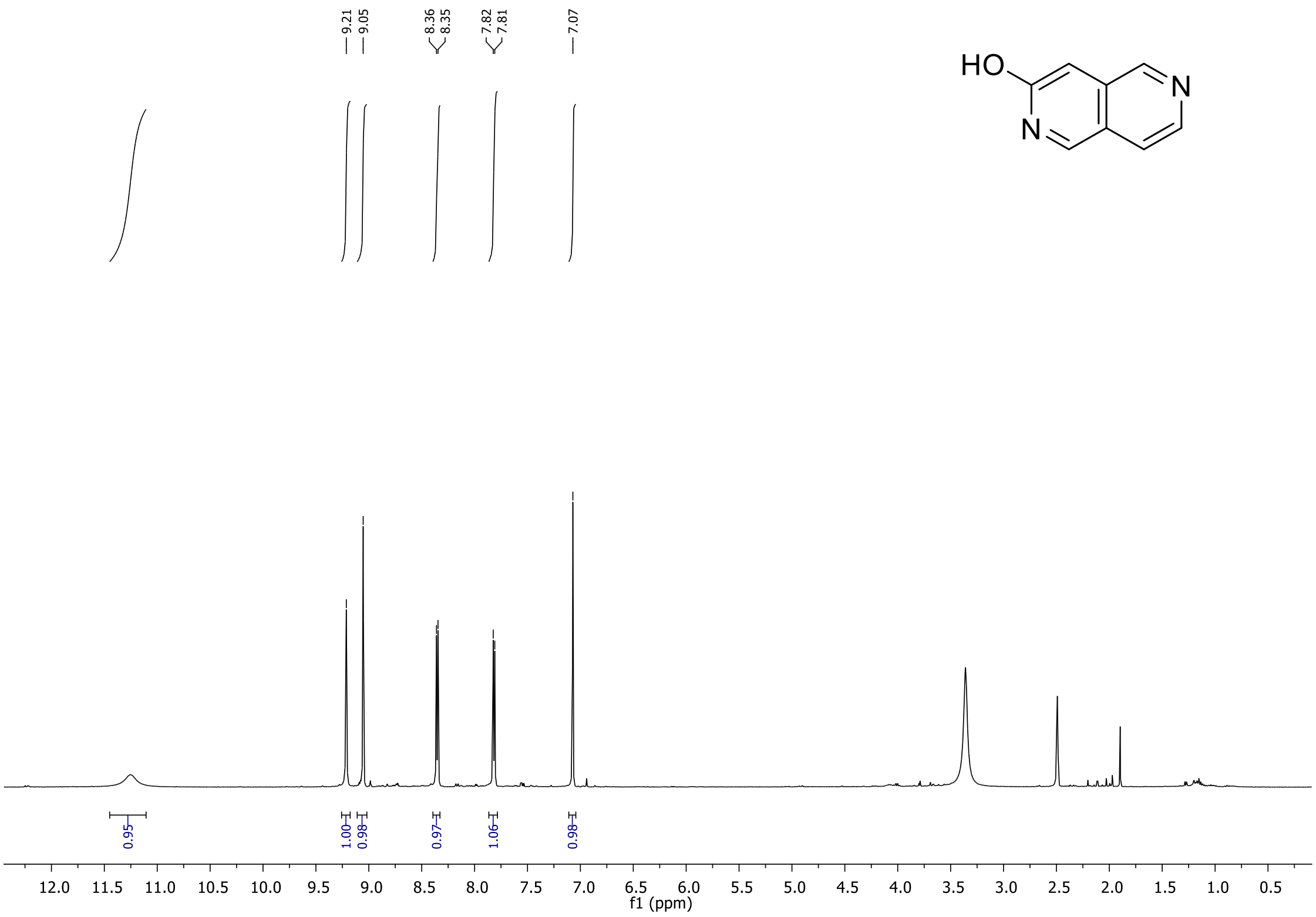

^{13}C NMR - 101 MHz in DMSO-d6: 2,6-naphthyridin-3-ol (2)

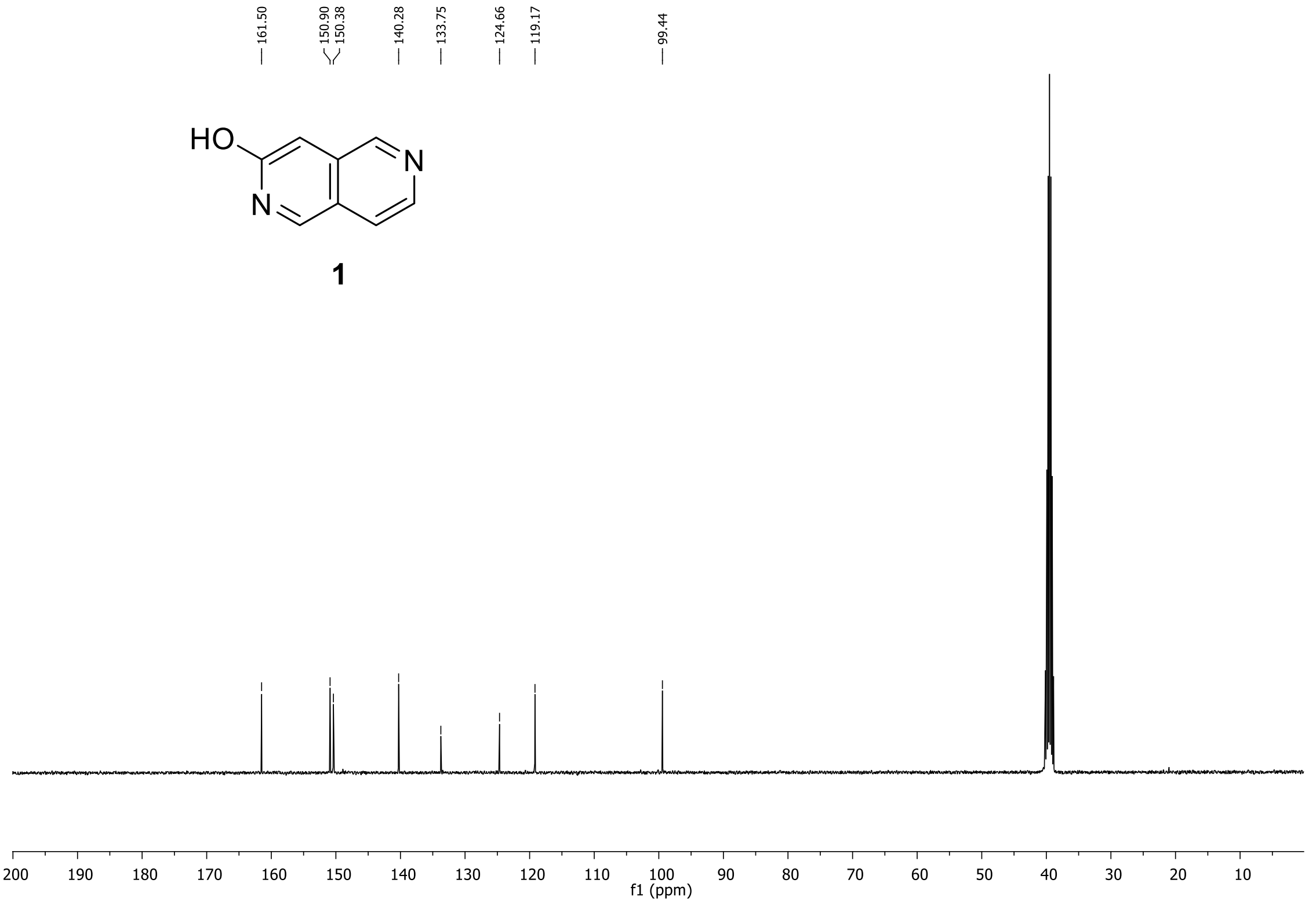

^{1}H-NMR – 300 MHz in $CDCl_3$: 3-(4-bromobutoxy)-2,6-naphthyridine (38)

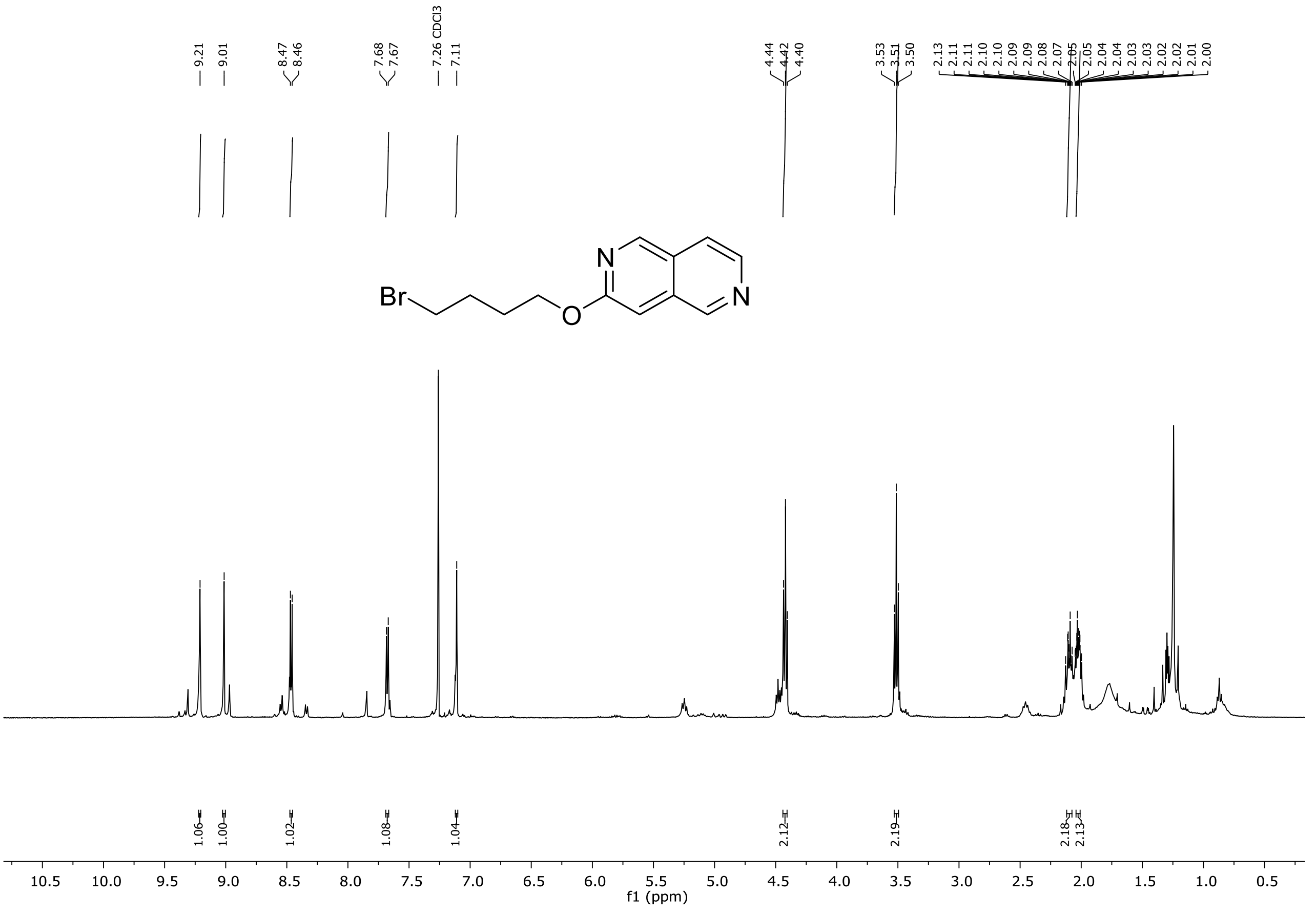

^{13}C-NMR – 75 MHz in $CDCl_3$: 3-(4-bromobutoxy)-2,6-naphthyridine (38)

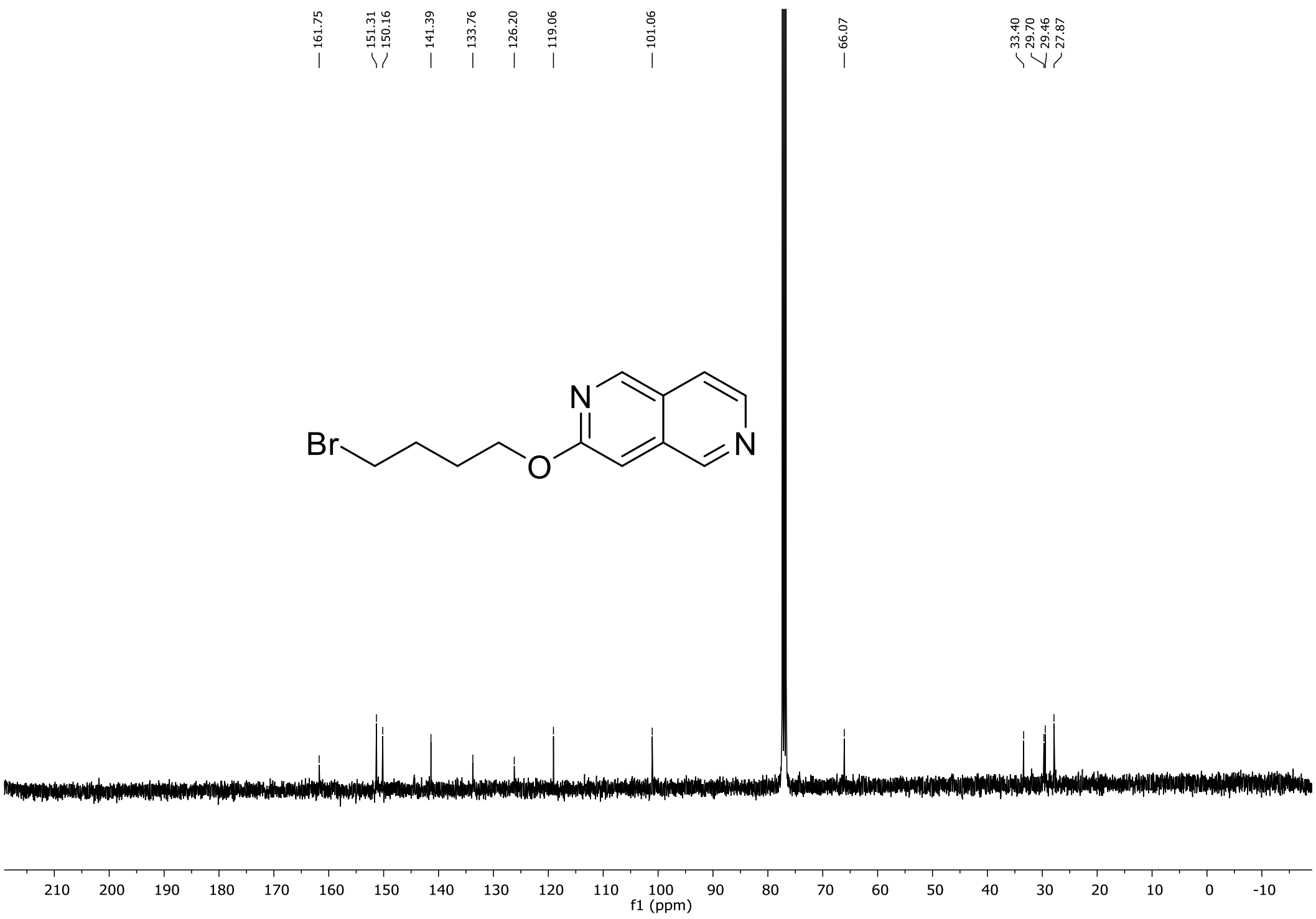

^{1}H NMR – 300 MHz in $CDCl_3$: 3-(4-(4-(2,3-dichlorophenyl)piperazin-1-yl)butoxy)-2,6-naphthyridine (40)

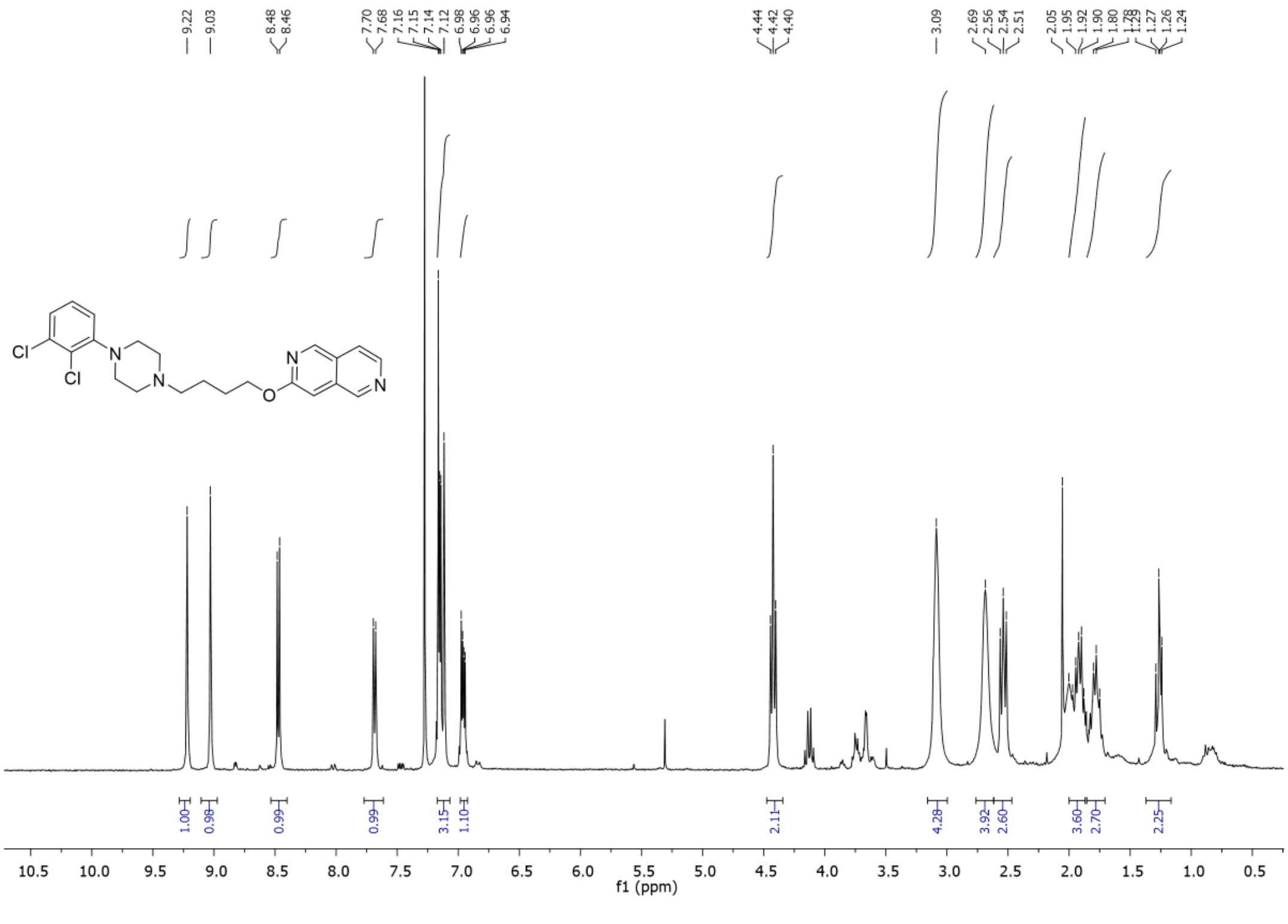

^{13}C NMR – 75 MHz in $CDCl_3$: 3-(4-(4-(2,3-dichlorophenyl)piperazin-1-yl)butoxy)-2,6-naphthyridine (40)

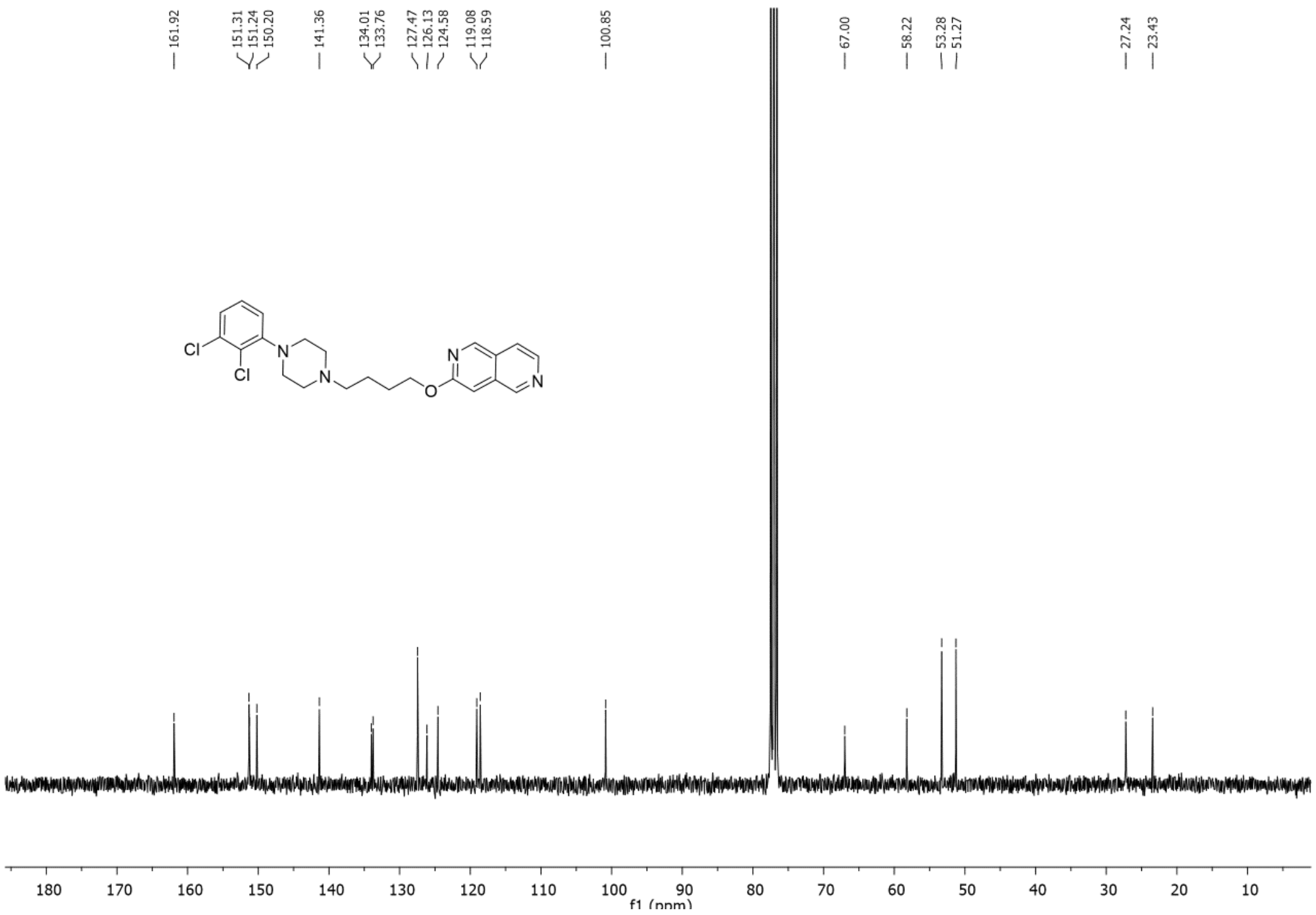

Purity of Most Active compounds

Method development for the characterization of compounds was carried out with a HPLC system. The detector was set at 254 nm. The system was controlled, and data analyses were performed using the LC solutions software. The solvents were filtered through a 0.45 µm Merck-Millipore filter before use and degassed in an ultrasonic bath. Volumes of 10 µL (analytical) and 1000 µL (semi-preparative) were injected. Quantification was carried out at 254 nm and the chromatographic run time varied according to each sample. Compound **40** was judged to be more than 95% pure by HPLC (UV at 254 nm).

Equipment: UFLC Shimadzu Prominence equipment; UV detector: SPD-M20A; auto sampler: SIL-20A; pump: LC-20AD.

solvent: A: None

B: CH_3CN

guard column: Shim-pack GIST C18 Guard Column
column: Shim-pack GIST C18 Capillary Column – 250 mm × 4.6 mm × 5 µm, Endcapped, pore size 10 nm or 100 Å; surface area 350 m²/g,
pH range: 1.0 to 10.0, carbon loading: 10%.
temperature: 35 °C
gradient:

time [min]	solvent A [%]	solvent B [%]
6.5	0.0	100.0

flow rate: 0.5 mL/min
injection: 10.0 uL
detection: wavelength: 254 nm
stop time: 6.5 min
calculation: integration: manual
calculation method: area %

HPLC of 3-(4-(4-(2,3-dichlorophenyl)piperazin-1-yl)butoxy)-2,6-naphthyridine

Sample Name	:		
Sample ID	:		
Data Filename	: QHM 159 FINAL.lcd		
Method Filename	:		
Batch Filename	:		
Vial #	:	Sample Type	: Unknown
Injection Volume	: 0 uL		
Date Acquired	: 31/12/1969 21:00:00	Acquired by	:
Date Processed	: 18/08/2026 16:20:23	Processed by	: QHETEM

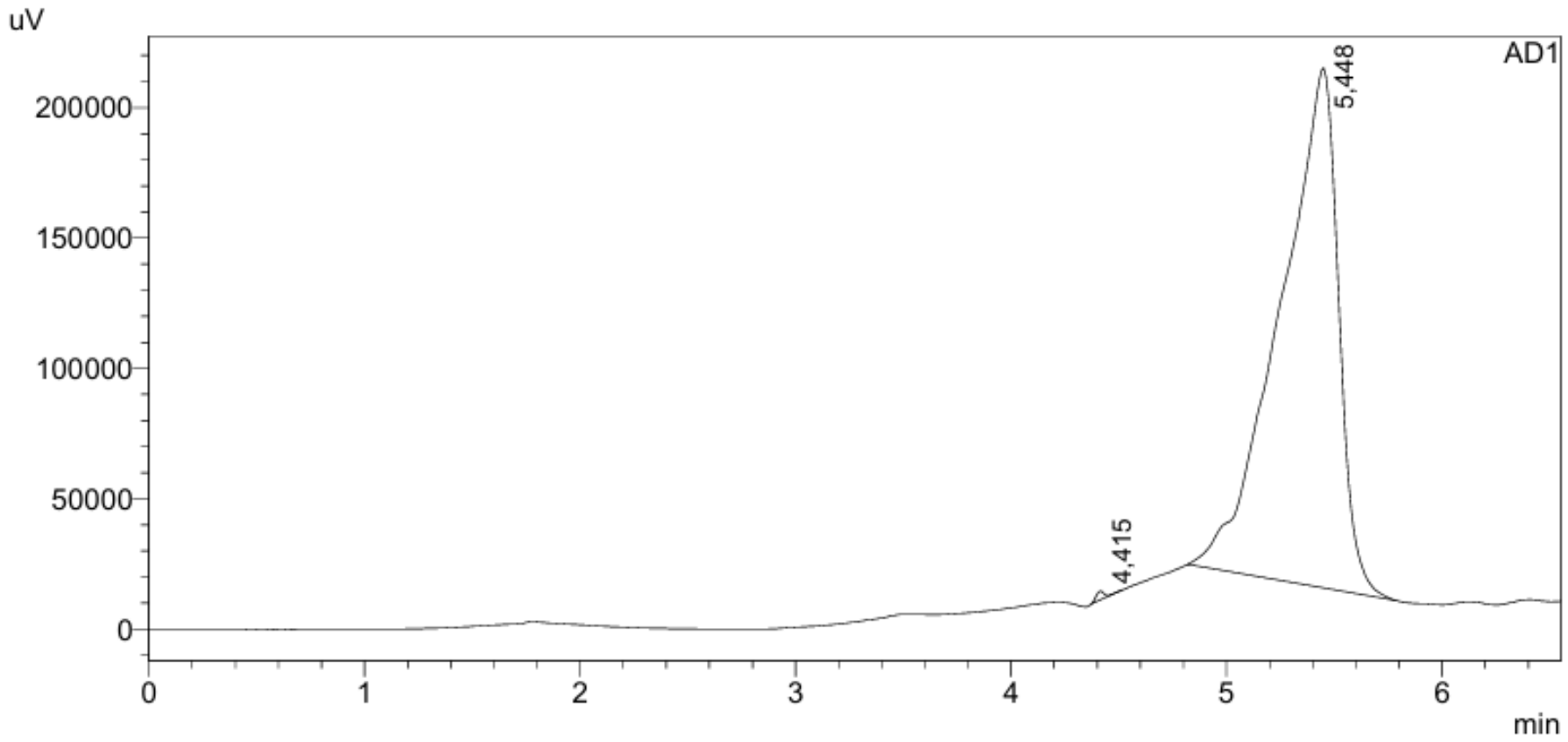

AD1

Peak#	Ret. Time	Area	Height	Area%	Peak Purity Index
1	4,415	10712	3375	0,3	
2	5,448	3747460	199289	99,7	
Total		3758172	202664	100,0	
